# Clathrin adapters AP-1 and GGA2 support expression of epidermal growth factor receptor for cell growth

**DOI:** 10.1038/s41389-021-00367-2

**Published:** 2021-11-19

**Authors:** Takefumi Uemura, Takehiro Suzuki, Naoshi Dohmae, Satoshi Waguri

**Affiliations:** 1 Department of Anatomy and Histology, Fukushima Medical University School of Medicine, 1 Hikarigaoka, Fukushima, Fukushima 960-1295 Japan; 2 Biomolecular Characterization Unit, RIKEN Center for Sustainable Resource Science, 2-1 Hirosawa, Wako, Saitama 351-0198 Japan

**Keywords:** Autophagy, Membrane trafficking

## Abstract

The role of Golgi/endosome-localized clathrin adapters in the maintenance of steady-state cell surface epidermal growth factor receptor (EGFR) is not well known. Here, we show that EGFR associates preferentially with both AP-1 and GGA2 in vitro. AP-1 depletion caused a reduction in the EGFR protein by promoting its lysosomal degradation. Triple immunofluorescence microscopy and proximity ligation assays demonstrated that the interaction of EGFR with AP-1 or GGA2 occurred more frequently in Rab11-positive recycling endosomes than in Rab5-positive early endosomes. Biochemical recycling assay revealed that the depletion of AP-1 or GGA2 significantly suppressed EGFR recycling to the plasma membrane regardless of the EGF stimulation. Depletion of AP-1 or GGA2 also reduced cell contents of other tyrosine kinases, MET and ErbB4, and therefore, suppressed the growth of H1975 cancer cells in culture and xenograft model. Moreover, AP-1 was expressed in endosomes at higher levels in some cancer tissues. Collectively, these results suggest that AP-1 and GGA2 function in recycling endosomes to retrieve endocytosed EGFR, thereby sustaining its cell surface expression and, consequently, cancer cell growth.

## Introduction

The EGFR plays key roles not only in normal cellular functions but also in the development of several types of cancer cells [1, 2], and therefore, it is the therapeutic targets of several anti-cancer drugs. The fate of EGFR after ligand-binding has been intensively studied; activated EGFRs are internalized and transported into endosomes, where the receptors are ubiquitinated by CBL and translocated into internal vesicles by the action of ESCRT complexes, and finally transported into lysosomes for degradation. In contrast to this mechanism for downregulation, a fraction of EGFR that evades ubiquitination and degradation can recycle back to the plasma membrane (PM), which contributes to retaining the cell surface fraction of this receptor. Cell surface EGFR is also constitutively internalized and recycled back to the PM without ligand stimulation; this balance must be precisely regulated for the maintenance of the cell surface-dominant localization of the receptor [3, 4]. However, the molecular mechanisms of this steady-state recycling of EGFR are poorly understood.

Clathrin adapter molecules link specific transmembrane proteins with a clathrin scaffold, thereby regulating the efficient sorting and transport of proteins as cargo. These adapter molecules are classified into monomeric and heterotetrameric clathrin adapters; the former include three Golgi-localized, γ-adaptin ear-containing, ADP ribosylation factor-binding proteins 1, 2, and 3 (GGA1, 2, 3), and the latter contain five adapter protein complexes (AP-1, -2, -3, -4, -5). Each adapter is involved in distinct transport pathways of post-Golgi trafficking, such as AP-2 for the formation of clathrin-coated vesicles at the PM for endocytosis, and AP-1 and GGA1–3 for vesicular transport between the trans-Golgi network (TGN) and endosomes. The AP-1 complex is composed of four subunits, γ-, β1-, σ1-, and μ1-adaptin, and each monomeric GGA protein contains four distinct domains, VHS, GAT, hinge, and GAE. These subunits and domains can mediate binding to the Golgi membrane, specific cargo, clathrin, and various accessory proteins that modulate the adapters’ function. μ1-adaptin is characteristic of two types of AP-1; ubiquitously expressed AP-1A has a μ1A-adaptin subunit and epithelial-specific AP-1B contains μ1B (for more details refer to reviews, [5–7]).

The role of AP-1 together with GGAs in the transport of mannose 6-phosphate receptors (MPRs) between the TGN and endosomes, which mediates the efficient sorting of lysosomal hydrolases, has been well described [8, 9]. It is also accepted that AP-1 and GGAs are involved in the transport of other cargo, including TfR, LDLR, EGFR, sortilin, SorLA/LR11, LRP-3/9/12, and BACE1 [9–11]. It has been shown that GGA3 can sort ubiquitinated EGFR into late endosomes for degradation [12], and the basolateral sorting of EGFR is supported by AP-1B [13, 14]. Moreover, GGA2 reportedly maintains the cell surface expression of EGFR necessary for robust cell growth [15], and is recognized as a cooperative driver of EGFR-mediated lung adenocarcinoma [16], suggesting a new role of GGA2 in supporting cancer cell growth. However, to our knowledge, no study has explored the function of APs in the context of EGFR stability. Here, we present evidence that AP-1 together with GGA2 controls EGFR recycling from endosomes, maintaining its cell surface expression and cell growth.

## Results

### AP-1-depletion causes a reduction of EGFR protein

**Fig. 1:**
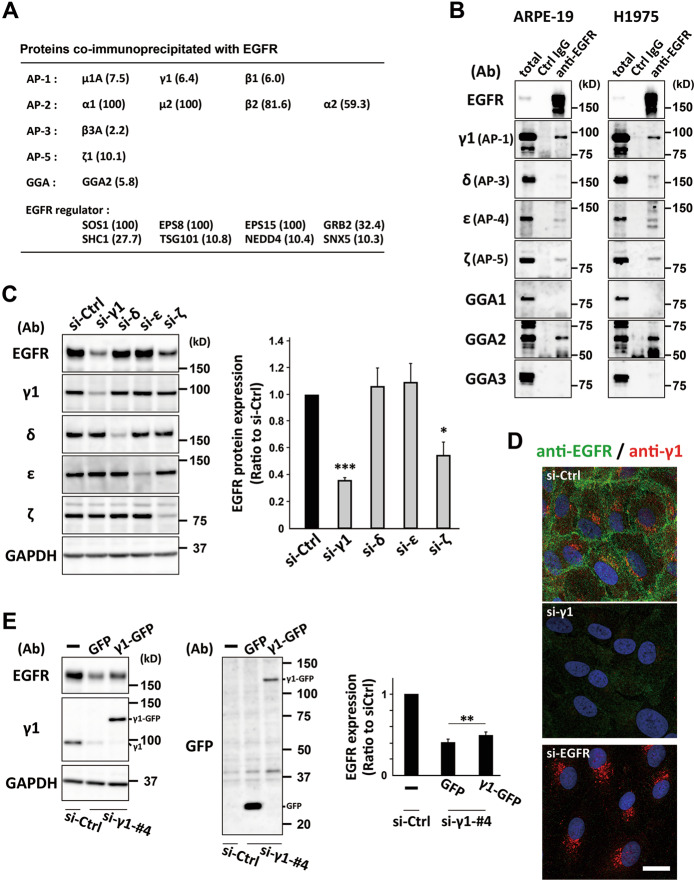
AP-1-depletion causes a reduction in EGFR protein. **A** A list of proteins co-immunoprecipitated with EGFR is shown. Clathrin adapter molecules and other EGFR regulators are extracted from Supplementary Table S1 and shown with their corresponding abundance ratio (anti-EGFR IgG/normal IgG) in parenthesis. **B** Immunoprecipitation of ARPE-19 and H1975 cell lysates with control (Ctrl) IgG or anti-EGFR antibodies. Approximately 0.8% of the input (total) and bound proteins were analyzed by western blotting with the indicated antibodies (Ab). **C** Western blotting of lysates of ARPE-19 cells transfected with siRNA for control (si-Ctrl), γ1-adaptin (si-γ1), δ-adaptin (si-δ), ε-adaptin (si-ε), or ζ-adaptin (si-ζ); the indicated antibodies (Ab) were used. The ratio of each siRNA to si-Ctrl was plotted on the right as mean ± SD (of three experiments). Statistical differences between each siRNA and si-Ctrl were analyzed using Student’s *t*-test (*P < 0.05, ***P < 0.001). **D** Double-immunofluorescence microscopy of cells treated with si-Ctrl, si-γ1, or siRNA for EGFR (si-EGFR) and incubated with anti-EGFR (green) and anti-γ1-adaptin (red) antibodies. Nuclei were stained with Hoechst 33342 (blue). Bar, 20 μm. **E** ARPE-19 cells treated with si-γ1-#4 that targets 3′UTR of the γ1 gene, were transfected with a plasmid encoding GFP or γ1-GFP devoid of the 3′UTR, or without plasmid (–), and then analyzed by western blotting with the indicated antibodies (Ab). The ratio of each group to si-Ctrl was plotted on the right as mean ± SD (of four experiments). The statistical difference between GFP and γ1-GFP was analyzed using Student’s *t*-test (**P < 0.01).

To identify the clathrin adapters involved in EGFR trafficking under normal culture conditions containing 10% fetal bovine serum, which is defined as “steady-state” in this study, we first obtained EGFR-binding proteins by immunoprecipitation coupled with mass spectrometry using lysates of a normal epithelial cell line ARPE-19. We found that, in addition to well-known interactors such as SOS-1 and AP-2, several Golgi/endosome-localized adapters, AP-1, 3, and 5 and GGA2, were able to bind to EGFR (Fig. 1A and Supplementary Table S1). The immunoprecipitates were then examined by western blotting, together with those from the H1975 cell line that is derived from non-small-cell lung carcinoma (NSCLC) and has an L858R/T790M double mutation in the EGFR gene [17]. These assays revealed a strong EGFR interaction with AP-1 and GGA2 and a weak interaction with AP-4 and AP-5, and little interaction with AP-3, GGA1, or GGA3 in both cell-types (Fig. 1B). GGA2 has previously been shown to associate with EGFR and stabilize receptor expression [15, 16], and as such we examined the role of AP-1, -3, -4, and -5 in EGFR stabilization. When ARPE-19 cells were treated with siRNAs against γ1 (AP-1)-, δ (AP-3)-, ε (AP-4)-, and ζ (AP-5)-adaptin, γ1- and ζ-adaptin depletions led to marked decrease of EGFR (Fig. 1C). We hereafter focused on γ1-adaptin, as it appeared to affect protein expression of EGFR more than ζ-adaptin. The reduction of EGFR induced by γ1-adaptin depletion was confirmed by immunofluorescence microscopy (Fig. 1D). Similar results were observed following independent application of three additional siRNAs for γ1-adaptin in both western blotting and immunofluorescence microscopy (Supplementary Fig. S1A, B). Moreover, the depletion of γ1-adaptin significantly decreased other AP-1 subunits, β1-, μ1A-, and σ1A-adaptins; while depletion of γ2-adaptin which is functionally distinct from γ1-adaptin [18–20] did not affect their expression (Fig. S1C). Importantly, depletion of other subunits, µ1 or β1-adaptin also reduced EGFR (Supplementary Fig. S1D), and reexpression of γ1-adaptin slightly restored EGFR in Western blot analysis (Fig. 1E) and immunofluorescence microscopy (Supplementary Fig. S1E), excluding a possibility of off-target effects. These results indicate that the depletion of AP-1 causes a reduction of EGFR protein in ARPE-19 cells.

### AP-1-depletion accelerates lysosomal degradation of EGFR protein

**Fig. 2:**
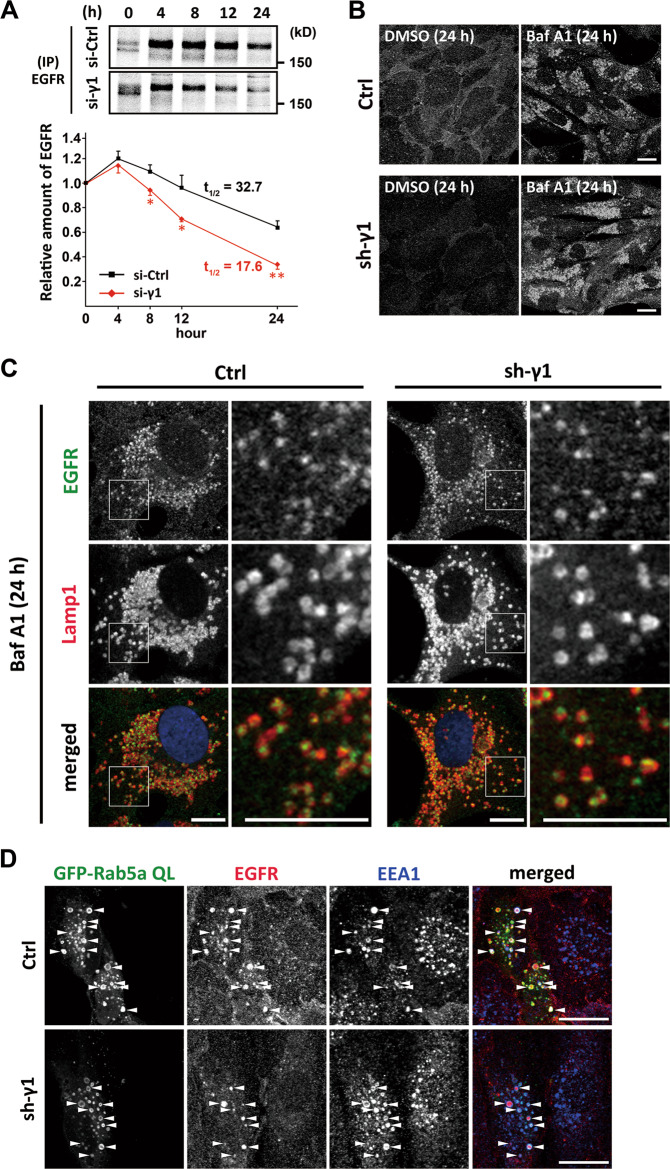
AP-1-depletion accelerates lysosomal degradation of EGFR protein. **A** ARPE-19 cells transfected with si-Ctrl or si-γ1 were pulse-labeled with [^35^S]-methionine/cysteine for 2 h and chased for the indicated periods. The cell lysates were immunoprecipitated (IP) with anti-EGFR antibodies, followed by SDS-PAGE. The lower band of the doublet at 0 h corresponds to non-glycosylated precursor form of EGFR. The value of each time point was normalized to that at 0 h chase, and plotted on the graph as the mean ± SD (of three experiments). Statistical differences between si-Ctrl and si-γ1 at each time point were analyzed using Student’s *t*-test (*P < 0.05 and **P < 0.01). **B** Control (Ctrl) and AP-1-depleted (sh-γ1) ARPE-19 cells were treated with 100 nM bafilomycin A1 (Baf A1) or DMSO for 24 h, and fixed for immunofluorescence microscopy using anti-EGFR antibodies. Bars, 20 μm. **C** Ctrl and sh-γ1 cells were treated with 100 nM Baf A1 for 24 h, and fixed for double immunofluorescence microscopy using anti-EGFR (green) and anti-Lamp1 (red) antibodies. Nuclei were stained with Hoechst 33342 (blue). Bars, 10 μm. **D** Ctrl and sh-γ1 cells transfected with GFP-Rab5a QL (green) were fixed for double immunofluorescence microscopy with anti-EGFR (red) and anti-EEA1 (blue). Arrowheads indicate colocalization of three signals. Bars, 20 μm.

Pulse-chase experiments with [^35^S]-methionine/cysteine followed by immunoprecipitation of EGFR indicated a significantly increased turnover rate of EGFR in AP-1 depleted cells (*P* < 0.01–0.05; Fig. 2A). The quantity of mRNA and newly translated EGFR protein was unchanged after AP-1 depletion (Supplementary Fig. S2). Cells were constitutively depleted of AP-1 by shRNA-γ1-adaptin (sh-γ1), and subsequent treatment with the lysosomal inhibitor bafilomycin A1 resulted in a slight accumulation of cellular EGFR similar to the control cells by western blotting (Supplementary Fig. S2C). Since the modest increase might be due to the release of EGFR-containing exosomes by bafilomycin A1 [21–24], we then examined intracellular localization. Immunofluorescence microscopy showed a remarkable accumulation of EGFR in numerous punctate structures (Fig. 2B), which colocalized with a lysosome marker Lamp1 in both control and sh-γ1 cells (Fig. 2C). Moreover, when GFP-Rab5a Q79L that is known to cause an enlargement of early endosomes because of their transport dysfunction was overexpressed, EGFR was trapped in GFP-positive structures in most (>96%) of both sh-γ1 and control cells (Supplementary Fig. S2D), and the aberrant structures were labeled with an early endosome marker EEA1 or a lysosomal marker cathepsin D (Fig. 2D and Supplementary Fig. S2E). These results indicate that, not only in control cells but also in AP-1 depleted cells, EGFR is transported through endosomal compartments into lysosomes. Together with the increased turnover rate of EGFR by AP-1 knockdown (Fig. 2A), it is suggested that AP-1 depletion does not cause a drastic disturbance in the early endocytic pathway of EGFR, but enhances its transport from the endocytic pathway into lysosomes for degradation.

### Depletion of AP-1 or GGA2 downregulates cell surface expression of EGFR

**Fig. 3:**
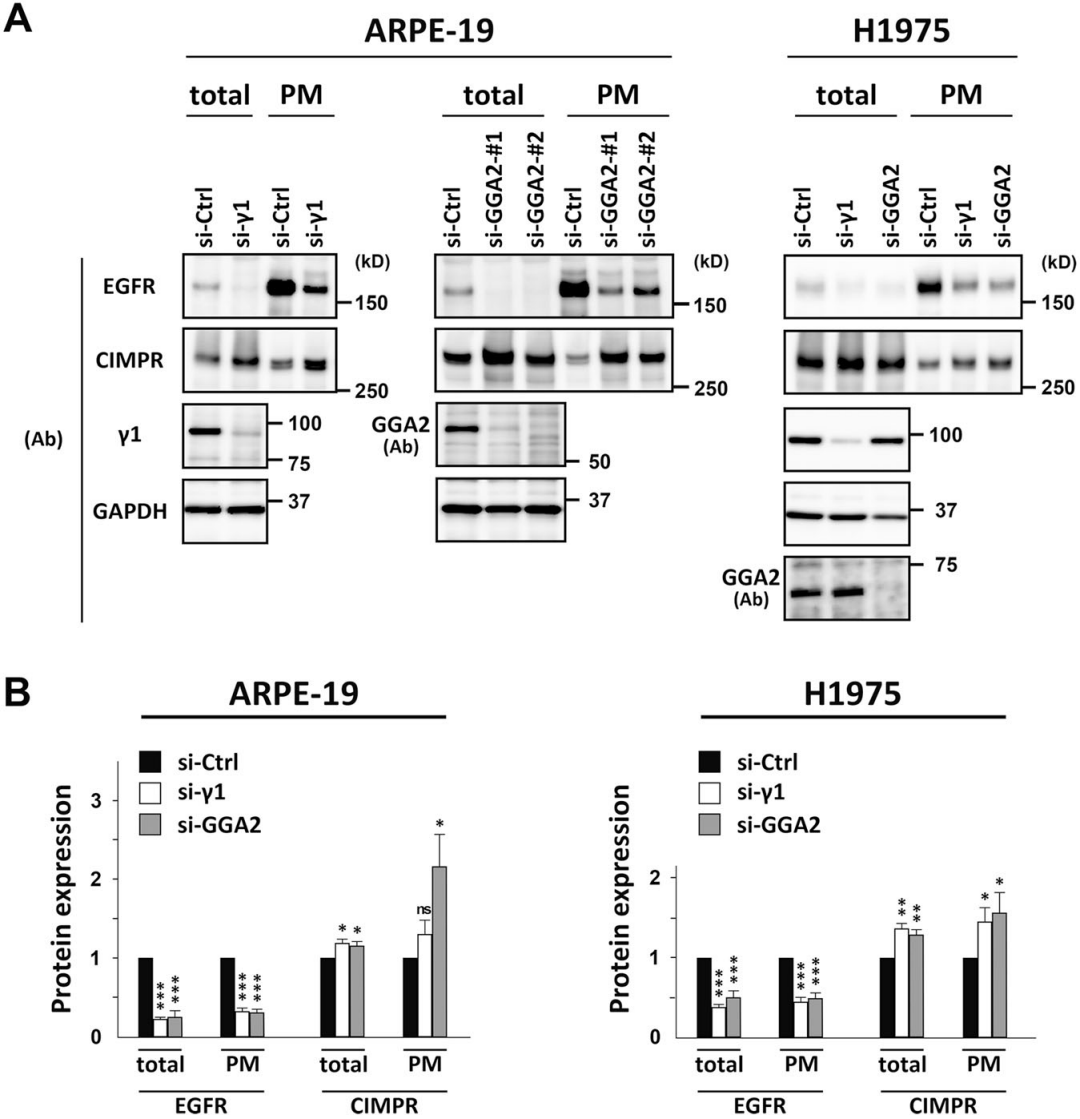
Depletion of AP-1 or GGA2 downregulates cell surface expression of EGFR. **A** Western blotting of ARPE-19 and H1975 cells transfected with si-Ctrl, si-γ1, or siRNA for GGA2 (si-GGA2-#1 or -#2). After biotinylation of surface proteins, lysates of the transfectants (total) and the PM fraction (PM) were analyzed using the antibodies (Ab) against EGFR, CIMPR, γ1-adaptin (γ1), GGA2, and GAPDH. Ten percent of the input was applied in the total lane. **B** The ratio of si-γ1 or si-GGA2#2 to si-Ctrl was plotted as the mean ± SD (of three and four experiments for ARPE-19 and H1975, respectively). Statistical differences between each siRNA and si-Ctrl were analyzed using Student’s *t*-test (*P < 0.05, **P < 0.01, and ***P < 0.001, ns: not significant).

The above results are similar to what we observed previously for GGA2 [15]. Therefore, we examined the extent to which the depletion of AP-1 or GGA2 could reduce cell surface EGFR by using a surface-biotinylation experiment. As shown in Fig. 3, EGFR in the cell surface was significantly decreased (*P* < 0.001) by depletion of either AP-1 or GGA2 in both ARPE-19 and H1975 cells, which is comparable with the decrease in the total protein. One of well-known cargoes of AP-1/GGA2, cation independent (CI) MPR was rather increased by the depletion of AP-1/GGA2 in both total and cell surface fraction, which is consistent with previous data [25–27]. Together, this assay indicates that the depletion of AP-1 or GGA2 reduces the cell surface amounts as well as the total amounts of EGFR protein.

### AP-1 and GGA2 interact with EGFR at the recycling endosomes

**Fig. 4:**
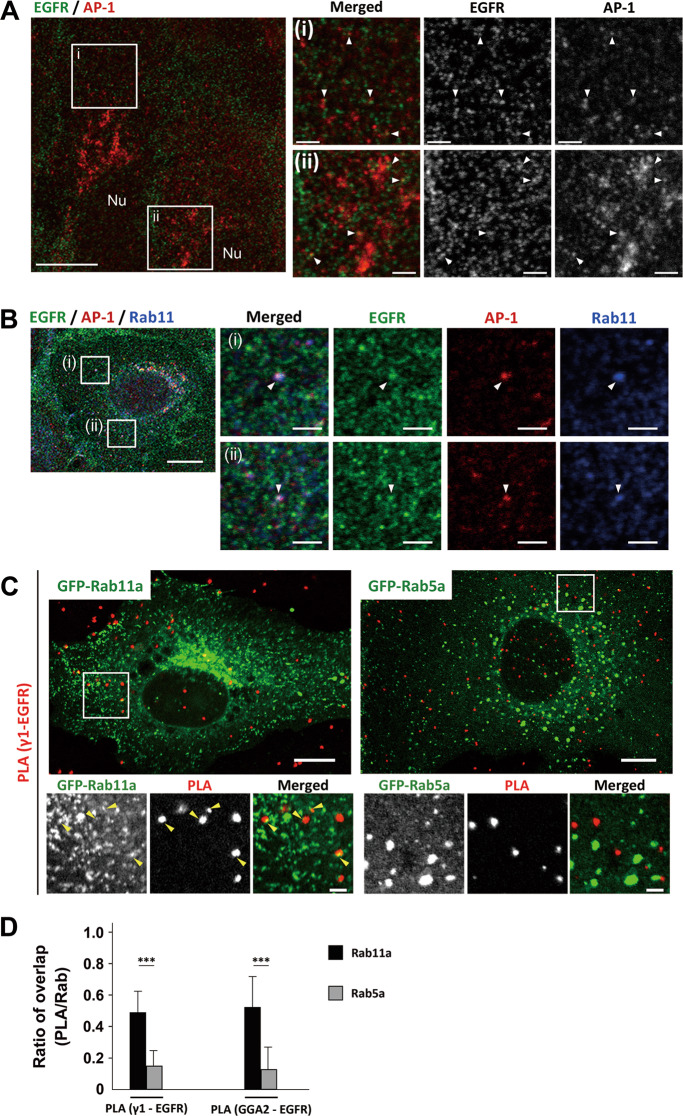
AP-1 and GGA2 interact with EGFR at the recycling endosomes. **A** Double immunofluorescence microscopy of ARPE-19 cells immunostained with anti-EGFR (green) and anti-γ1 (red) antibodies. Boxed regions (i) and (ii) are magnified and shown in the right. Arrowheads indicate colocalization of both signals. Nu: nucleus, bars: 10 μm (left) and 2 μm (magnified images). **B** Triple immunofluorescence microscopy of ARPE-19 cells immunostained with anti-EGFR (green), anti-γ1 (red), and anti-Rab11 (blue) antibodies. Boxed regions (i) and (ii) are magnified and shown in the right. Arrowheads indicate colocalization of three signals. Nu: nucleus, Bars: 10 μm (left) and 2 μm (magnified images). **C** PLA of ARPE-19 cells transfected with GFP-Rab11a or GFP-Rab5a (green). Transfectants were processed for PLA with a combination of anti-γ1-adaptin and anti-EGFR (γ1–EGFR). In this experiment, cells with very low expressions of the GFP-fusion proteins were selected, and thus the laser beam power was set at 2–5-folds of conventional observations. Boxed regions are magnified and shown below. Yellow arrowheads indicate the overlap of both signals. Bars: 10 μm (top) and 2 μm (bottom). **D** PLA signal that overlapped with GFP-Rab11a or GFP-Rab5a was quantified and plotted as the fraction of total PLA signal (mean ± SD; *n* = 35 [γ1-EGFR/Rab11a and GGA2/Rab5a], 34 [GGA2/Rab11a], and 38 [γ1-EGFR/Rab5a]). Statistical differences were analyzed using Student’s *t*-test (***P < 0.001). PLA for GGA2–EGFR interaction is shown in Supplementary Fig. S4E.

We next investigated intracellular localization of AP-1, GGA2, and EGFR by immuno-labeling methods. When cytoplasmic EGFR was carefully analyzed by double immunofluorescence microscopy, a small population of EGFR-positive puncta was labeled with AP-1 (Fig. 4A) or GGA2 (Supplementary Fig. S3A). Since AP-1 has been shown to localize to Rab11-positive recycling endosomes [28], and ligand-stimulated EGFR is recycled back to the PM through Rab11-positive endosomes [29, 30], we examined if three molecules, EGFR, AP-1, and Rab11, could show colocalization in the cytoplasm. When endogenous AP-1 and Rab11 was first examined, AP-1 preferentially colocalized with Rab11 in ARPE-19 cells using an antibody that specifically detects Rab11a/b, while the colocalization with a marker of early endosome, Rab5, was not so evident (Supplementary Fig. S3B, C). Also, a small population of EGFR was colocalized with Rab11 in these cells (Supplementary Fig. S3C). Finally, triple immunofluorescence microscopy confirmed that both AP-1 and Rab11 colocalized on the EGFR-positive puncta though with low frequencies (Fig. 4B). Similarly, GGA2 also colocalized with a small population of EGFR together with Rab11 (Supplementary Fig. S3D).

To obtain more solid information of in vivo interactions between AP-1/GGA2 and EGFR, we applied proximity ligation assay (PLA). Punctate signals for the AP-1–EGFR interaction were scattered throughout the cytoplasm of ARPE-19 and H1975 cells, and were diminished by knockdown of EGFR or AP-1 (Supplementary Fig. S4A, B). In contrast, those for AP-1–CIMPR interaction were primarily found in the juxtanuclear Golgi regions (Supplementary Fig. S4A). As an additional negative control, PLA signal for the interaction between AP-1 and COPI (β-COP) or COPII (Sec24B) was significantly lower than that between AP-1 and EGFR (Supplementary Fig. S4B). These results indicate that the PLA specifically detects AP-1–EGFR interaction. Interestingly, the PLA signal did not significantly alter after EGF treatment (Supplementary Fig. S4C), suggesting the interaction occurs independently of the EGF signaling. We then examined if the AP-1–EGFR interaction is preferentially observed in Rab11-positive recycling endosomes. Since PLA is difficult to combine with immunolabeling methods, GFP-Rab11a or GFP-Rab5a was introduced to the cells. We confirmed that the two GFP-fusion proteins showed similar distribution pattern of endogenous proteins (Supplementary Figs. S3C and S4D), and that AP-1 in the peripheral region was preferentially colocalized with GFP-Rab11a compared with GFP-Rab5a (Supplementary Fig. S4D). As shown in Fig. 4C, the PLA-positive puncta for AP-1–EGFR interactions were strikingly well colocalized with structures positive for Rab11a, but only apposed or not associated with those for Rab5a. Of further interest, PLA-positive puncta for GGA2–EGFR interactions demonstrated the same colocalization pattern (Supplementary Fig. S4E). Quantification revealed that about 49–52% of the signal for AP-1/GGA2–EGFR interaction overlapped with Rab11-positive structures, while it was 13–15% with Rab5-positive ones (Fig. 4D), indicating that the interactions between AP-1/GGA2 and EGFR occur preferentially in the recycling endosomes.

### AP-1 and GGA2 support recycling of EGFR back to the PM

**Fig. 5:**
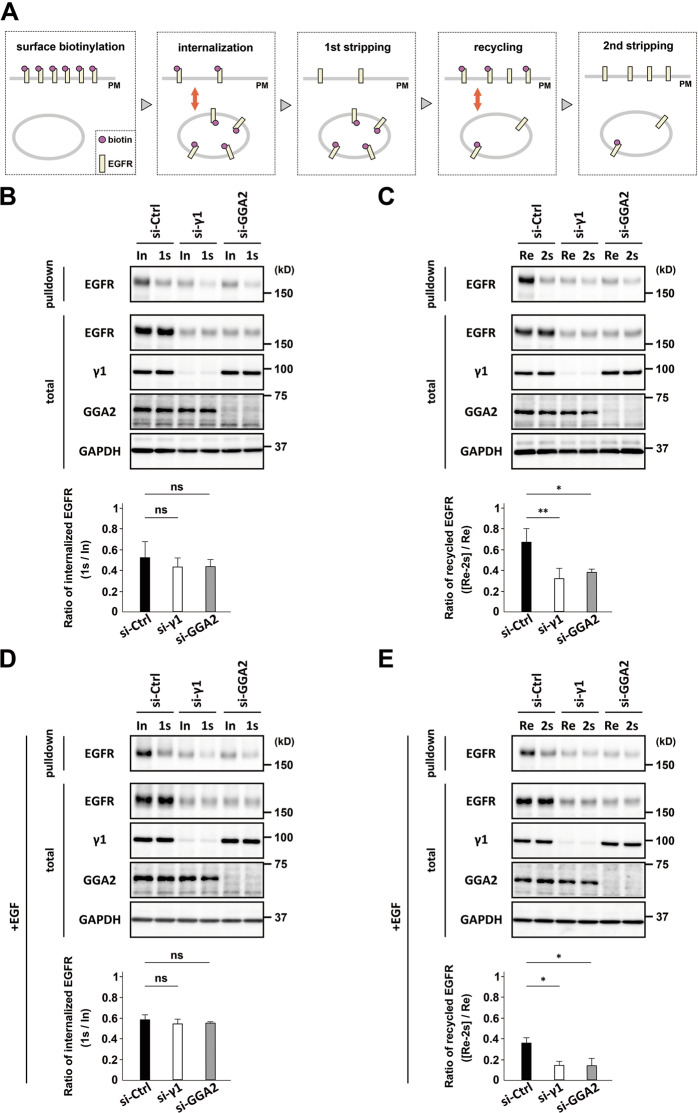
AP-1 and GGA2 support EGFR recycling back to the PM. **A** Schematic description of the EGFR recycling assay. Serum starved cells were labeled with Sulfo-NHS-SS-biotin (pink circle, surface biotinylation), and incubated with DMEM for 30 min at 37 °C (**B** and **C**), or DMEM containing 10 nM EGF for 10 min at 37 °C (**D** and **E**) for internalization. Afterward, cells were treated with a reducing reagent to remove biotin from EGFR (yellow bar) on the PM (1st stripping). Cells were re-incubated with DMEM at 37 °C for 30 min to allow recycling of internalized biotinylated EGFR, followed by the reducing reagent (2nd stripping). **B**–**E** ARPE-19 cells transfected with si-Ctrl, si-γ1, or si-GGA2 were treated as above. After “internalization (In)”, “1st stripping (1s)”, “recycling (Re)”, and “2nd stripping (2s), cell lysates were prepared (total), and biotinylated proteins were collected using avidin-agarose (pulldown), which were examined by western blotting using antibodies as indicated. For internalization assay, the ratio of the biotinylated EGFR (pulldown) for “1s” to that for “In” was calculated, and plotted as the mean ± SD of three experiments in the graphs (**B** and **D**). For recycling assay, the value obtained by subtracting “2s” from “Re” was considered as the EGFR that recycled back to the PM. The ratio of this value to “Re” was calculated and plotted as the mean ± SD of four and three experiments in the graphs of (**C**) and (**E**), respectively. Statistical differences between the si-Ctrl and si-γ1 or si-GGA2 were analyzed using Student’s *t*-test (*P < 0.05, **P < 0.01, ns: not significant).

**Fig. 6:**
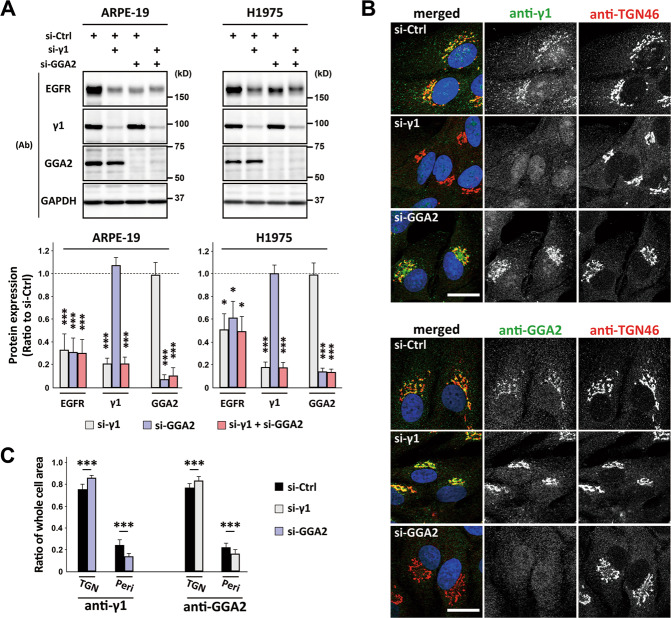
Cellular distribution of either AP-1 or GGA2 depends on the other’s expression. **A** Western blot of ARPE-19 and H1975 cells transfected with si-Ctrl, si-γ1, or si-GGA2 alone, or a mix of si-γ1 and si-GGA2; the indicated antibodies (Ab) were used. The ratio of each siRNA to si-Ctrl is plotted as the mean ± SD (of six and three experiments for ARPE-19 and H1975, respectively). Statistical differences between each siRNA and si-Ctrl were analyzed using Student’s *t*-test (*P < 0.05 and ***P < 0.001). **B** ARPE-19 cells transfected with si-Ctrl, si-γ1, or si-GGA2 were fixed for double immunofluorescence microscopy using antibodies against TGN-46 (red) and γ1-adaptin (green) or GGA2 (green), as indicated. Nuclei were stained with Hoechst 33342 (blue). Bars, 20 μm. **C** Fluorescence intensities for γ1-adaptin (anti-γ1) or GGA2 (anti-GGA2) in both TGN and peripheral (Peri.) regions were quantified in 40 cells for each siRNA experiment. Statistical differences between each siRNA and si-Ctrl were analyzed using Student’s *t*-test (***P < 0.001).

The above result prompted us to measure the recycling rate of EGFR under serum-starved and EGF-treated condition in ARPE-19 cells (Fig. 5A). We first confirmed that the internalization rate was not significantly affected by the depletion of AP-1 or GGA2 (Fig. 5B). This also suggests that 60–70% of the EGFR reduction (Figs. 1C and 6A) by the AP-1/GGA2 knockdown does not cause an experimental bias in this assay. On the other hand, the recycling fraction of EGFR 30 min after the endocytosis was significantly lower in AP-1- or GGA2-depleted cells than in control cells (Fig. 5C; *P* < 0.05 or 0.01). Another RTK, Eph2, that also undergoes endocytosis and recycling to the PM [31], did not show significant alterations by the depletion of AP-1 or GGA2 (Supplementary Fig. S5A, B). Moreover, when this assay was performed at an EGF-stimulated condition, EGFR recycling was also significantly reduced in AP-1- or GGA2-depleted cells (Fig. 5D, E). These results together with the PLA data under EGF stimulation as described above (Supplementary Fig. S4C) suggest that AP-1 and GGA2 support the recycling of EGFR regardless of the EGF stimulation.

### Cellular distribution of either AP-1 or GGA2 depends on the other’s expression

Since GGA2 depletion causes accelerated EGFR degradation [15], we examined the relationship between AP-1 and GGA2. Depletion of either AP-1 or GGA2 did not affect expression levels of the other (Fig. 6A), as reported previously [32, 33], nor did simultaneous depletion of both adapters enhance the reduction of EGFR. However, immunofluorescence microscopy revealed that GGA2 depletion caused AP-1 distribution to shift from the peripheral to the TGN46-positive TGN region, and AP-1 depletion led to a slight accumulation of GGA2 in the TGN region (Fig. 6B, C). Together, these results indicate that the cellular distribution of either AP-1 or GGA2 depends on the other’s expression.

### Depletion of AP-1 or GGA2 causes downregulation of other receptor tyrosine kinase (RTK)

**Fig. 7:**
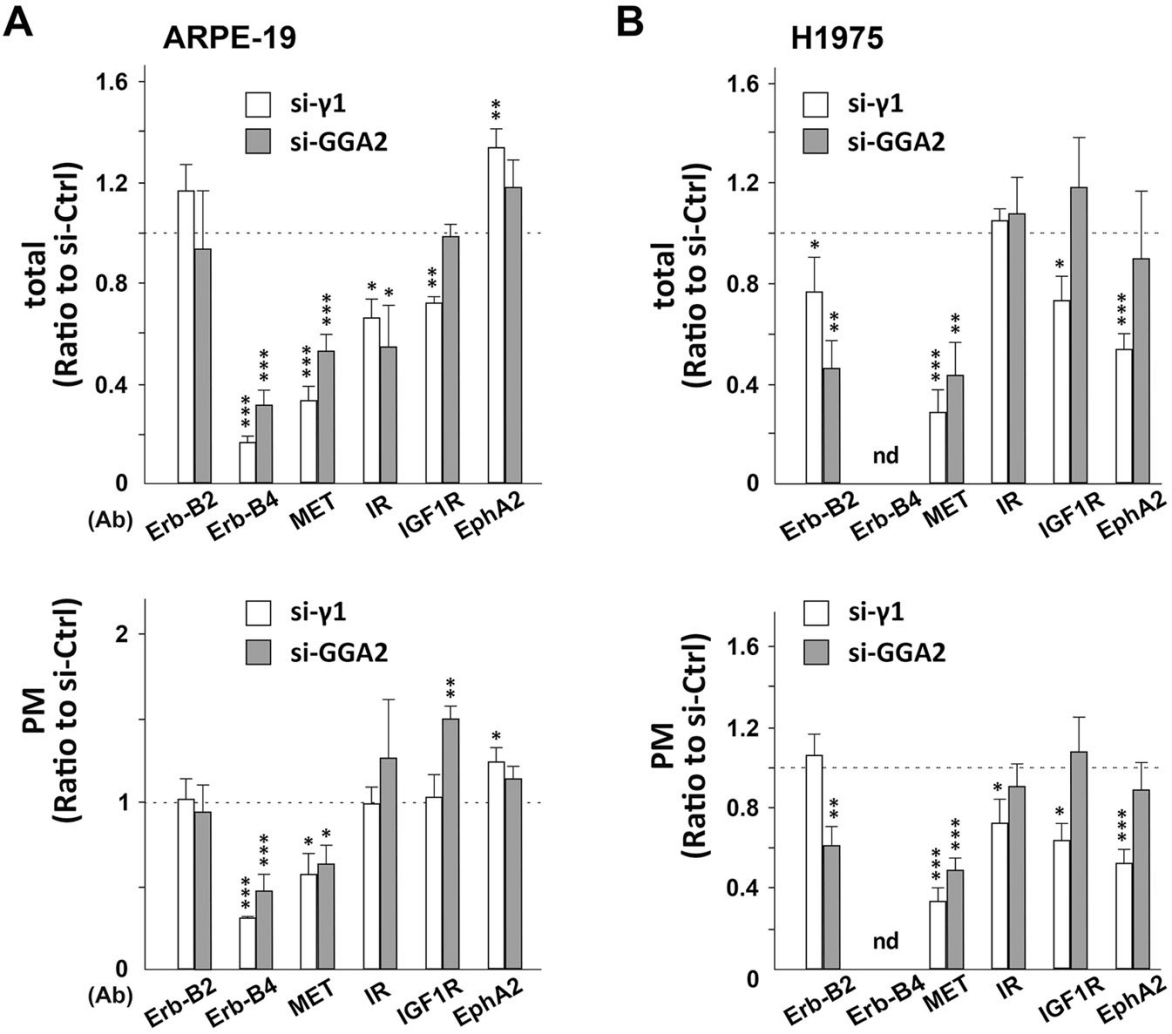
Depletion of AP-1 or GGA2 causes downregulation of other RTK proteins. **A**, **B** Quantitative data of western blotting of ARPE-19 (**A**) and H1975 (**B**) cells transfected with siRNA for control (si-Ctrl), γ1-adaptin (si-γ1), or GGA2 (si-GGA2). See the blot images in Supplementary Fig. S6A. After biotinylation of surface proteins, lysates of the transfectants (total) and the PM fraction (PM) were analyzed using antibodies against RTKs (Erb-B2, Erb-B4, MET, IR, IGF1R, and EphA2). The ratio of si-γ1 or si-GGA2 (corresponding to si-GGA2-#2 in Supplementary Fig. S6A) to si-Ctrl (indicated by the broken line) was plotted as the mean ± SD (of three and four experiments for ARPE-19 and H1975, respectively). Statistical differences between each siRNA and si-Ctrl were analyzed using Student’s *t*-test (*P < 0.05, **P < 0.01, and ***P < 0.001).

We next explored if the transport of other RTKs may be regulated by AP-1 or GGA2. Thus, both total and cell surface levels of other 6 and 5 RTKs were evaluated in ARPE-19 and H1975 cell lines, respectively, as described above (Fig. 3). We found that Erb-B4 and MET were also decreased significantly (*P* < 0.05 or 0.001) by depletion of either AP-1 or GGA2 in ARPE-19 cells (Fig. 7A, and Supplementary Fig. S6A). In H1975 cells that have minimal expression of Erb-B4 (Supplementary Fig. S6B), MET were significantly decreased by AP-1/GGA2 depletion (*P* < 0.01 or 0.001; Fig. 7B and Supplementary Fig. S6A). Alterations of IGF1R and EphA2 were more sensitive to AP-1 depletion, while decrease of Erb-B2 was more sensitive to GGA2-depletion in H1975 cells (Fig. 7A, B), suggesting different sorting functions of AP-1 and GGA2. Together, these assays clearly indicate that, although their preferences are different, both AP-1 and GGA2 regulate cellular contents and cell surface expression of a subset of RTKs in a cell-dependent manner.

### AP-1 supports cell growth in vitro and in vivo

**Fig. 8:**
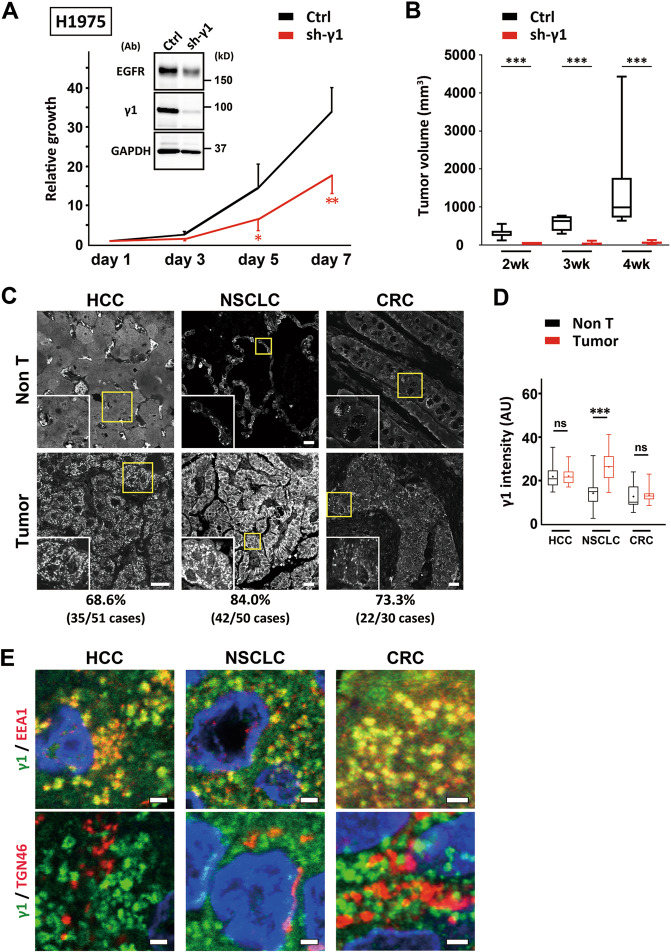
AP-1 supports cell growth and is expressed at high levels in endosomes of some human cancer tissues. **A** ARPE-19 and H1975 cells stably expressing sh-γ1 or vector (Ctrl) were subjected to cell proliferation assays. Depletion of γ1-adaptin and EGFR was confirmed by western blotting using the indicated antibodies (Ab). Data are plotted as the means ± SD of three experiments. Statistical differences between Ctrl and sh-γ1 at each time point were analyzed using Student’s *t*-test (*P < 0.05 and **P < 0.01). **B** Tumor volumes in xenograft experiments were measured at the indicated time points and displayed as box-and-whisker plots. The horizontal line inside each box represents the median value. The lowest point and highest point are the minimum and maximum of the data set, respectively. Statistical analyses were performed using Mann–Whitney *U*-test　(***P < 0.001). Images of tumors are shown in Supplementary Fig. S7C. **C** Immunostaining of tissue arrays with paraffin tumor sections (Tumor) and adjacent non-tumor regions (Non T) from HCC (grade 1), NSCLC (grade 1), and CRC (grade 2), and using anti-γ1-adaptin Ab. Boxed regions are magnified and shown in insets. Bars, 20 μm. The percentage of cases with more intense granular signal for AP-1 in tumors than those in non-tumor regions are indicated at the bottom with the total number of cases in parentheses. Detailed information is listed in Supplementary Table S2. **D** γ1 signal in normal (black) and tumor (red) tissue was quantified, and displayed as box-and-whisker plots. The horizontal line inside each box and ‘+’ represents the median value and mean, respectively. The lowest point and highest point are the minimum and maximum of the data set, respectively. Statistical differences between each normal and tumor tissue were analyzed using Mann–Whitney *U*-test (***P < 0.001, ns: not significant). **E** Double-immunostaining of tissue arrays of HCC (grade 1–2), NSCLC (grade 1–2), and CRC (grade 2) using combinations of anti-γ1-adaptin, and anti-EEA1 or anti-TGN46 Ab. See Supplementary Fig. S7E for lower magnification images. Bars, 2 μm.

We next examined the relationship between AP-1 and cell proliferation. Phosphorylation of MAPK, a downstream signaling of EGFR, was significantly reduced in AP-1-depleted ARPE-19 cells (Supplementary Fig. S7A). In cell culture conditions, growth rates of ARPE-19 or H1975 cells depleted of γ1-adaptin were significantly lower than those of control cells (*P* < 0.05–0.001; Fig. 8A and Supplementary Fig. S7B). In xenoplantation experiments using H1975 cells, AP-1-depletion significantly suppressed growth rates of tumors (*P* < 0.001; Fig. 8B and Supplementary Fig. S7C). H1975 cells harbor activating EGFR mutations [17], and as such, these results suggest the involvement of AP-1 in EGFR-dependent cell growth.

### AP-1 is expressed at high levels in endosomes of some human cancer tissues

Finally, we investigated the expression of AP-1 in tissue arrays of hepatocellular carcinoma (HCC), NSCLC, and colorectal adenocarcinoma (CRC). The specificity of the antibody against γ1-adaptin in recognizing the endogenous protein in paraffin-embedded human tissues was confirmed by using cell pellets of AP-1-depleted H1975 cells (Supplementary Fig. S7D). Cytoplasmic granular structures positive for AP-1 were more evident in tumor regions compared to non-tumor regions in 68–84% of cancer tissues (Fig. 8C). When the mean signal intensity was quantified, it was significantly higher in tumor than in non-tumor cells in NSCLC, but not in HCC or CRC (Fig. 8D), suggesting alterations in intracellular distribution rather than higher expression levels of AP-1 are characteristic for tumor cells. Moreover, double immunofluorescence microscopy demonstrated that punctate signals for AP-1 overlapped well with EEA1-positive endosomes, but not at all or only partially with the TGN marker TGN46 in HCC, NSCLC, and CRC (Fig. 8E and Supplementary Fig. S7E). These observations suggest that the recruitment of AP-1 to endosomes may be more relevant than that to the TGN in these cancers.

## Discussion

In this study, we explored the EGFR-related function of Golgi/endosome-localized clathrin adapters by simple proteomics and siRNA-based analyses. We found that AP-1 and AP-5, as well as GGA2 support the steady-state cell surface expression of EGFR. Strikingly, both AP-1 and GGA2 interact with EGFR at Rab11-positive recycling endosomes, and support the receptor recycling back to the PM regardless of the EGF stimulation. In addition, AP-1 and GGA2 also support cell surface expression of other tyrosine kinases, MET and ErbB4. Accordingly, cell growth was suppressed by the depletion of AP-1 in a non-cancer cell line, ARPE-19, and a NSCLC-derived cancer cell line, H1975, which harbors activating EGFR mutations [17]. Together with recent findings that the depletion of GGA2 suppresses cell proliferation in ARPE-19 [15], and two NSCLC-derived cancer cell lines, PC9 and H1975 [16], it is most likely that both AP-1 and GGA2 have a fundamental influence on cell growth by sustaining the expression of EGFR.

It has been reported that the μ1-adaptin subunit of AP-1 can recognize the tyrosine-based motif, Y^974^RAL, and YXXΦ-independent interfaces in the cytoplasmic domains of EGFR [34, 35], and that GGA2 is able to interact with the juxta-membrane domain of EGFR, although this domain does not contain a typical ACLL motif [15]. We confirmed these associations both in vitro and in vivo, and further found that these associations specifically occur in Rab11a-positive recycling endosomes, and support the EGFR recycling. The endosomal recycling pathway can be regulated by specific Rabs; Rab4 and Rab35 mediate direct recycling from early endosomes to the PM, while Rab11 and Rab11-family interacting proteins support indirect recycling through perinuclear endocytic recycling compartments [4]. AP-1 has been shown to localize to Rab11-positive recycling endosomes, thereby promoting retrograde transport of the cholera toxin B-subunit from endosomes to the Golgi complex [28]. Previous studies have demonstrated that ligand-stimulated EGFR is recycled back to the PM through Rab11-positive endosomes, which is promoted by the short form of EPS15 [30] and a tyrosine-phosphorylated component, Odin [29, 30]. By contrast, steady-state cell surface expression of EGFR can be regulated by an F-actin promoting factor WASH (Wiskott-Aldrich syndrome protein and Scar homolog) at endosomes [36], but the involvement of Rab11-positive endosomes has not been shown. It is unlikely that Rab11 itself is directly involved in the AP-1/GGA2-mediated EGFR recycling, because Rab11 knockdown did not cause significant reduction of EGFR (Supplementary Fig. S3B), and Rab11 was not found in the list of EGFR interacting molecules in mass analysis (Supplementary Table S1). Therefore, the present study provides the first evidence that shows a molecular link between AP-1/GGA2 and EGFR in Rab11-positive recycling endosomes in both the steady-state and EGF-stimulated conditions. Given that the lysosomal degradation of EGFR is increased and its recycling to the PM is decreased by the knockdown of AP-1 (present study) and GGA2 (present study and [15]), AP-1 and GGA2 play an important role in the maintenance of cell surface EGFR by regulating receptor recycling pathway. It should be noted that GGA3 has been previously shown to promote the lysosomal degradation of EGFR [12] after stimulation of EGF. Probably, GGA3 mainly functions in ligand-stimulated endocytosis and/or ubiquitin-dependent degradation of EGFR, since it hardly associated with EGFR at steady-state in this study (Fig. 1A). On the other hand, AP-1 and GGA2 could act in the recycling pathway of EGFR that evades ubiquitination, because they have lower affinities to ubiquitin compared with GGA3 [12]. Whether or not AP-1/GGA2 can interact with liganded and/or non-liganded EGFR remains as a future issue.

Molecular relationship between AP-1 and GGA2 along the recycling route could be of great interest. Although GGA2 associates with the juxtamembrane domain of EGFR [15], it may also recognize EGFR indirectly through AP-1, because the hinge domain of GGA2 can directly bind the ear domain of AP-1 [37]. The possible implication of AP-1-GGA2 interaction in the EGFR trafficking is partly supported by in vivo data in the present study showing that the depletion of either AP-1 or GGA2 affected the distribution of GGA2 or AP-1, respectively (Fig. 6). Further investigations are required to clarify the detailed molecular mechanisms of this action.

We found by immunohistological analysis that AP-1 was changed in its cellular distribution in many cases of HCC, NSCLC, and CRC, and also upregulated in NSCLC. Moreover, AP-1 is primarily localized to EEA1-positive endosomes in HCC, NSCLC, and CRC, which is in line with the recycling endosome-localized AP-1 observed in cultured cells. Previous studies have reported cell growth-promoting functions of AP-1 in *Arabidopsis* [38] and of μ1A-adaptin (AP1M1) in hepatitis B virus-transfected HepG2 cells [39]. Moreover, μ1A-adaptin has been recently identified by proteomics analysis as a possible predictive marker of metastasis to the central nervous system in triple-negative breast cancer [40]. GGA2 gene has been identified as a cooperative driver of EGFR-mediated lung adenocarcinoma [16], and GGA2 protein has been reported to be upregulated in HCC and CRC [15]. Therefore, accumulating evidence including our data suggests the importance of AP-1/GGA2 in cancer growth. Moreover, since the growth of NSCLC-derived H1975 cells used in this study is known to depend on the EGFR, growth suppression by AP-1 depletion both in vitro and in vivo (Fig. 8A, B) would provide strong evidence that the effect was mediated by the EGFR depletion. However, predicted growth effects in cancer tissues may not be mediated solely by the upregulation of EGFR. For example, the minimum levels of activated EGFR may be enough to maintain the growth of some cancers, as has been demonstrated by observing HSC3 cells expressing endogenous levels of EGFR-GFP in mouse tumor xenografts [41]. The present study also suggests AP-1/GGA2 can support the expression of other RTKs, MET and ErbB4, which might have a significant impact on cancer growth in HCC, since MET is known to be overexpressed in this type of tumor [42]. It should also be mentioned that the AP-1 complex can function when it is recruited onto the membrane compartments, and this may be the reason why high endosomal contents of AP-1 was not simply reflected by its total amounts measured by histological quantifications (Fig. 8D). Thus, to establish the importance of AP-1/GGA2 in cancer progression the clathrin adapter-associated molecules, such as a small GTPase Arf1 that is necessary for membrane recruitment of AP-1, and several accessory proteins need to be investigated using human samples. Nevertheless, it should be emphasized that potential AP-1/GGA2 targeting as an anti-cancer strategy is not dependent on activation mutations but on the intracellular recycling of EGFR, and that AP-1/GGA2 targeting could arrest multiple cancer-related signaling pathways that originate from different RTKs.

## Materials and methods

### Cell culture and reagents

ARPE-19, H1975, and HEK293T cells were purchased from ATCC. They were grown in Dulbecco’s modified Eagle’s medium (DMEM; Nacalai Tesque, Kyoto, Japan) supplemented with 10% fetal bovine serum (FBS) at 37 °C in 5% CO_2_. Bafilomycin A1 was purchased from Merck (Kenilworth, NJ, USA). For treatment with EGF, cells were washed three times with PBS and cultured without serum for 22–24 h, then EGF (Cell Signaling Technology, Danvers, MA, USA) was added at 10 nM.

### Human samples

Tissue arrays of human carcinoma were purchased from Shanghai Outdo Biotech Co (Shanghai, China). Detailed information is provided in Supplementary Table S2. Studies on human samples were approved by the Institutional Review Board in Fukushima Medical University (approval number: 2943). For each human sample, informed consent was properly obtained as stated by the supplying companies (http://www.outdobiotech.com/strictsops.html).

### Immunoprecipitation

Cells were lysed with PBS containing 1% Triton X-100, a protease inhibitor cocktail (Roche, Basel, Switzerland), and phosphatase inhibitors (Roche). After centrifugation at 21,600 × *g* for 10 min, the supernatants were incubated with protein A sepharose for 0.5 h at 4 °C. After centrifugation, the supernatants were incubated with sepharose beads conjugated with antibodies against EGFR (Cell Signaling Technology) or control IgG (Cell Signaling Technology) for 20–24 h at 4 °C on a rotator. After six washes with the lysis buffer, the beads were collected, from which bound proteins were eluted with 1x SDS-PAGE sample buffer by boiling for 10 min at 90 °C.

### Immunoprecipitation mass spectrometry (IP-MS)

Eluted proteins as described above were applied to SDS-PAGE, and electrophoresis was stopped when the samples had traveled to the top of the separation gel. The gel was stained with Coomassie Brilliant Blue and the protein bands at the top of the separation gel were excised and digested in-gel with trypsin and applied to nLC-MS/MS using Q-Exactive HF-X mass spectrometer (ThermoFisher Scientific, Waltham, MA, USA) as described previously [43]. The abundance ratio (protein ratio) was calculated as the ratio of (anti-EGFR mouse)/(normal mouse IgG), and the maximum value was defined as 100 and the minimum as 0.01.

### Assays for EGFR internalization and recycling

The recycling assay was performed according to the method of Li et al. [44] with some modifications. Briefly, ARPE-19 cells were cultured for 22–24 h without serum. After rinsing three times with cold PBS, cells were incubated with 0.5 mM EZ-Link Sulfo-NHS-SS-biotin (ThermoFisher) in PBS on ice for 30 min, and with TBS on ice for 15 min, followed by incubation with DMEM for 30 min at 37 °C or DMEM containing 10 nM EGF for 10 min at 37 °C to induce internalization of biotinylated EGFR. Cells were incubated with a stripping solution (50 mM glutathione, 75 mM NaCl, 75 mM NaOH, 1% BSA, and 10 mM EDTA) on ice for 15 min to remove biotin remaining on cell surface. The cells were re-incubated at 37 °C in DMEM for 30 min to allow recycling of internalized EGFR, followed by a second treatment with or without the stripping solution. Preparation of cell lysates, pulldown using avidin-conjugated agarose, and western blotting were performed as previously described [15]. To calculate the internalization ratio, the amount of biotinylated EGFR after the 1st stripping was divided by that without the stripping reagent. To obtain the ratio of recycled EGFR to internalized EGFR, the biotinylated EGFR after the 2nd stripping was subtracted from that without the stripping reagent, and the value was further divided by that without the stripping. This experimental assay was confirmed to be carried out within a range, where the band intensity is linearly proportional to the protein concentration.

Other methods are described in Supplementary Materials and Methods.

## Supplementary information

Supplementary Materials and Methods, and Figures

Supplementary Table 1

Supplementary Table 2
