# Supplementary material for: Clathrin adapters AP-1 and GGA2 support expression of epidermal growth factor receptor for cell growth
Source: Oncogenesis. 2021 Nov 19;10(11):80. doi: 10.1038/s41389-021-00367-2 (PMC8604998; doi:10.1038/s41389-021-00367-2)
Supplement: Supplementary file 1 — Supplementary Materials and Methods, and Figures [file 41389_2021_367_MOESM1_ESM.pdf]

Supplementary information

Supplementary Materials and Methods

Antibodies

The antibodies and their sources used in this study were as follows: rabbit antibodies against EGFR (Cell Signaling Technology, Danvers, MA, USA), β-adaptin (Proteintech, Chicago, IL, USA), μ1A-adaptin (Proteintech), γ2-adaptin (Sigma, St. Louis, MO, USA), σ1A-adaptin (Sigma), ζ-adaptin (Sigma), EEA1 (Cell Signaling Technology; Abcam, Cambridge, UK), Erb-B2 (Cell Signaling Technology), Erb-B4 (Cell Signaling Technology), EphA2 (Cell Signaling Technology), Rab5 (Cell Signaling Technology), Rab11 (Cell Signaling Technology), MAPK (Cell Signaling Technology), pMAPK (Cell Signaling Technology), Sec24B (Cell Signaling Technology), Lamp1 (Cell Signaling Technology), GGA1 (Santa Cruz Biotechnology, Dallas, TX, USA), MET (Santa Cruz Biotechnology), IR (Santa Cruz Biotechnology), IGF1R (Santa Cruz Biotechnology), β-COP (ThermoFisher Scientific, Waltham, MA, USA), and CIMPR (Epitomics, Burlingame, CA, USA); mouse antibodies against EGFR (Millipore, Billerica, MA, USA), GAPDH (Santa Cruz Biotechnology), γ1-adaptin (BD Transduction Laboratories, San Jose, CA, USA), δ-adaptin (BD Transduction Laboratories), ε-adaptin (BD Transduction Laboratories), GGA2 (BD Transduction Laboratories), GGA3 (BD Transduction Laboratories); sheep antibody against TGN46 (Novus Biologicals, Littleton, CO, USA) and EGFR (MyBioSoure, San Diego, CA, USA).

DNA constructs and transfection

GFP-Rab5a (WT/Q79L) expression plasmid was a generous gift from Mitsunori Fukuda (Tohoku University, Japan). The cDNA for Rab11a (NCBI gene ID: 8766) was cloned from the cDNA library of ARPE-19, and inserted between EcoRV and XhoI of pcDNA3.1 vector carrying GFP between HindIII and EcoRI sites. For the expression experiments the cells were cultured overnight and transfected with the plasmids using Fugene HD (Promega, Madison, WI, USA) for 24 hours.

qPCR analysis

The TaqMan Gene Expression assay was performed using following probes: γ1-adaptin: Hs00964419_m1, σ1A-adaptin: Hs00611076_m1, EGFR: Hs01076078_m1, ACTB: Hs01060665_g1, Applied Biosystems, Foster City, CA, USA), and TaqMan Gene Expression Master Mix (Applied Biosystems).

<u>Knockdown by siRNA and shRNA</u>

Target sequences of siRNAs were as follows:
Human γ1-adaptin -#2 (5′-AGGAAGUUAUGUUCGUGAU-3′),
Human γ1-adaptin -#3 (5′-GCGAGUCCUAGCCAUAAAU-3′),
Human γ1-adaptin -#4 (5′-ACAGCCAGAAUCAACAUUC-3′),
Human GGA2-#1 (5′-GGUUUCCGGAAGACAUCAAGA-3′),
Human GGA2-#2 (5′-GGAGUUCUGCUGUACAAACAG-3′),

siRNAs against the following genes were obtained from Dharmacon (Lafayette, CO, USA): human γ1-adaptin-#1 (L-019183-00-0005), human γ2-adaptin (L-011552-01-0005), human β1-adaptin (L-011200-00-0005), human μ1-adaptin (L-013196-00-0005), human δ-adaptin (L-016014-00-0005), human ε-adaptin (L-021474-00-0005), human ζ-adaptin (L-025284-01-0005), human EGFR (L-003114-00-0005). siRNAs against Rab11 were obtained from Ambion (Austin, TX, USA); human Rab11a (s16703), human Rab11b (s17649).

Unless otherwise indicated, the knockdown experiments for γ1-adaptin and GGA2 were conducted by using γ1-adaptin-#1 and GGA2-#2 siRNAs, respectively. siRNAs were introduced as previously described [32]. For constitutive knockdown of γ1-adaptin, the pLKO.1 puro vector (Sigma) containing γ1-adaptin shRNA (the target sequence corresponding to the γ1-adaptin-#2 target sequence) were constructed according to the manufacturer's instruction. Production of stably knock-down cells using lentiviral vector were performed as previously described [15].

<u>Other biochemical analyses</u>

Pulse-chase experiment using a mixture of [^{35}S] methionine/cysteine (PerkinElmer, Waltham, MA, USA), western blot analysis, and surface-biotinylation were performed as previously described [15].

Analysis of cell growth *in vitro*

Cell growth assays was performed according to the methods described previously [15], except that cells were seeded in 96-well plates in hexaplicate at a density of 400 cells/well.

Proximity ligation assay (PLA)

PLA was conducted as previously reported [15]. For quantification of PLA signal in ARPE-19 cells expressing GFP-Rab11a or GFP-Rab5a, cells with very low expressions of the GFP-fusion proteins were selected, and thus when using laser scanning microscope the laser beam power was set at 2~5-folds of conventional observations.

Immunofluorescence microscopy

Immunofluorescence microscopy for cell lines and paraffin embedded tissues was performed as previously described [15, 32], except that a confocal microscope (FV1000, Olympus) equipped with a high sensitivity GaAsP detector unit (Olympus) was used for detection of cytoplasmic signals for EGFR, AP-1, GGA2, and Rab11 in double or triple immunofluorescense microscopy. To prepare paraffin-embedded cell pellets, H1975 cells treated with siRNAs for control or γ1-adaptin (#2) were fixed with 4% paraformaldehyde in 0.1 M PB, and then scraped and solidified using iPGell (Genostaff, Tokyo, Japan). Rescue experiments were conducted as previously described [15]. Briefly, two days after transfection with si-γ1-#4 that targets 3'UTR of the γ1 gene, cells were transfected with a plasmid encoding γ1-GFP devoid of the 3'UTR. They were fixed and immunostained with anti-EGFR antibody (Millipore) without permeabilization.

Xenograft experiments

Twenty female BalB/c nu/nu nude mice (5-6-week-old, obtained from CLEA Japan) were used for this study. H1975 cells were stably transfected with control or γ1 shRNA. Cell suspensions were adjusted to 6×10^7 cells/ml in PBS, and were subcutaneously injected into the right and left sides of the backs of mice (6×10^6 cells per injection site). Tumor sizes were measured using a digital caliper weekly for 4 weeks, and tumor volumes were calculated according to the following formula: $V = ab^2/2$, where "a" and "b" represent the

length and width of the xenograft tumor, respectively. This study was approved by the Ethics Committee for Animal Research of Fukushima Medical University (approval number: 2020113). All animal experiments were performed in accordance with the guidelines and regulations of Fukushima Medical University.

Quantification and Statistical analysis

In the experiments for western blotting, immunoprecipitation of radio-labeled proteins using culture cells, the intensity of each band was quantified using ImageJ and normalized to that of GAPDH. For colocalization analysis in PLA, PLA signal that overlapped with GFP-Rab11a or GFP-Rab5a was counted in 34-38 cells. For quantifications in the rescue experiment and the immunostaining for human samples, the mean intensity for EGFR or AP-1 signal per cell was quantified using ImageJ software. Statistical differences were analyzed using Student's *t*-test (*: $P < 0.05$, **: $P < 0.01$, and ***: $P < 0.001$).

Supplementary information

Supplementary Figure S1 (related to Fig. 1)

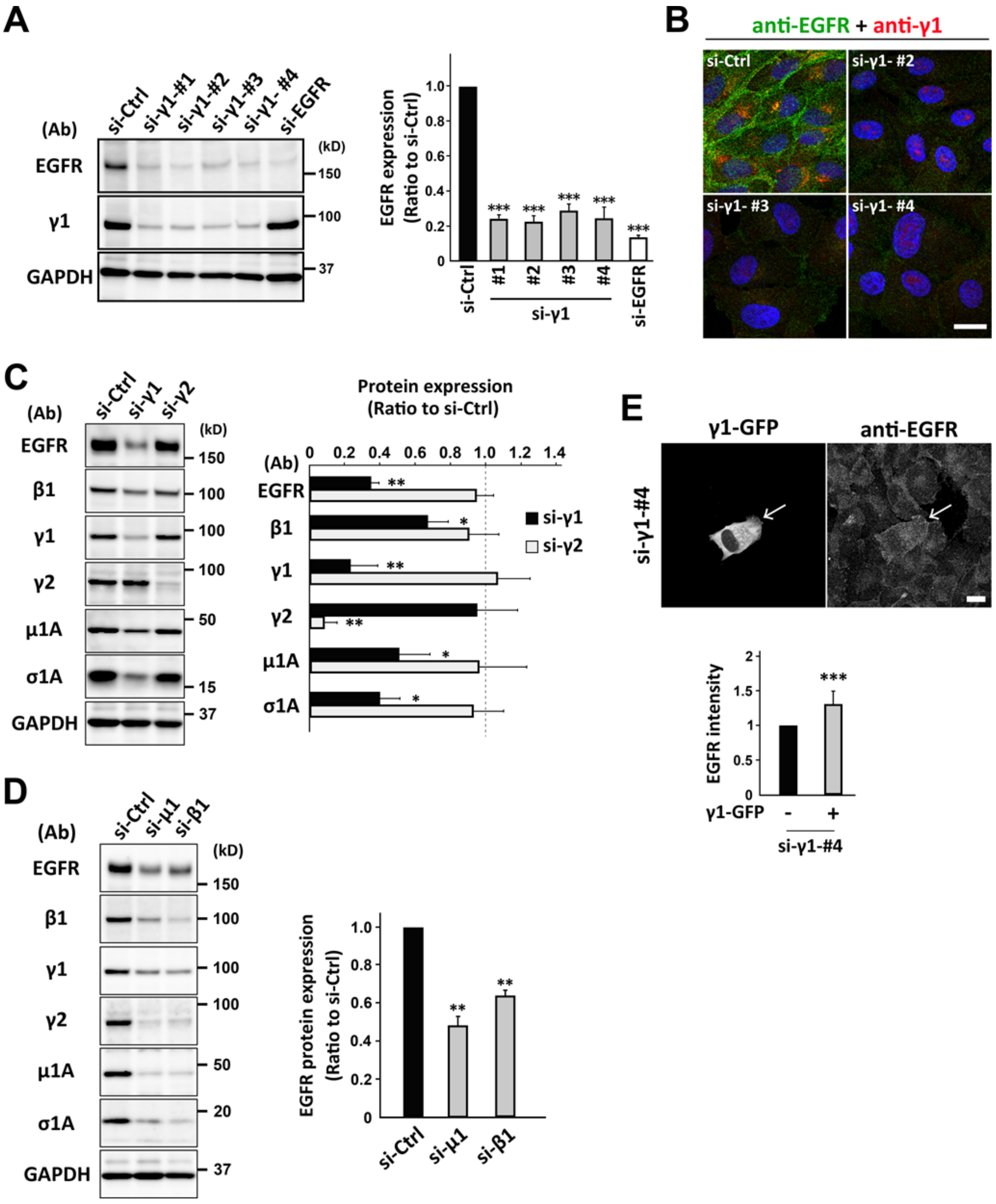

Figure S1 (related to Fig. 1) AP-1-depletion causes a reduction of EGFR protein

(A) Western blotting of ARPE-19 cells transfected with si-Ctrl, si-EGFR, or one of four independent siRNAs for γ-adaptin (si-γ1-#1－#4); the indicated antibodies (Ab) were used. The ratio of each siRNA to si-Ctrl was plotted on the right as the mean ± SD (of three experiments). (B) Immunofluorescence microscopy of cells that were transfected with si-Ctrl, or each of three siRNAs for γ1-adaptin (si-γ1-#2 to #4). Proteins were stained using anti-EGFR (green) and anti-γ1-adaptin (red) antibodies. Nuclei were stained with Hoechst 33342 (blue). Bar, 20 μm. (C) Western blotting of total lysates of ARPE-19 cells transfected with si-Ctrl, si-γ1, or siRNA for γ2-adaptin (si-γ2); indicated antibodies (Ab) were used. The ratio of si-γ1 or si-γ2 to si-Ctrl (indicated by broken line) was plotted on the right as mean ± SD from three experiments. (D) Western blotting of total lysates of ARPE-19 cells transfected with si-Ctrl, or siRNA for μ1-adaptin (si-μ1), or β1-adaptin (si-β1); indicated antibodies (Ab) were used. The ratio of si-μ1 or si-β1 to si-Ctrl was plotted on the right as mean ± SD from three experiments. In (A), (C) and (D), statistical differences between each siRNA and si-Ctrl were analyzed by Student's *t*-test. (*: $P < 0.05$, **: $P < 0.01$, and ***: $P < 0.001$). (E) ARPE-19 cells treated with si-γ1-#4 that targets 3'UTR of the γ1 gene, were transfected with a plasmid encoding γ1-GFP devoid of the 3'UTR, and then immunostained without permeabilization using anti-EGFR. Fluorescence signal was enhanced to detect reduced levels of EGFR signal. Cell surface signal of EGFR was quantified in γ1-GFP-positive ($n = 111$) and -negative cells ($n = 370$). Cells with modest levels of expression for γ1-GFP were chosen in this experiment. Statistical analysis was performed using Student's t-test (***$P < 0.001$). Bar, 20 μm.

Supplementary information

Supplementary Figure S2 (related to Fig. 2)

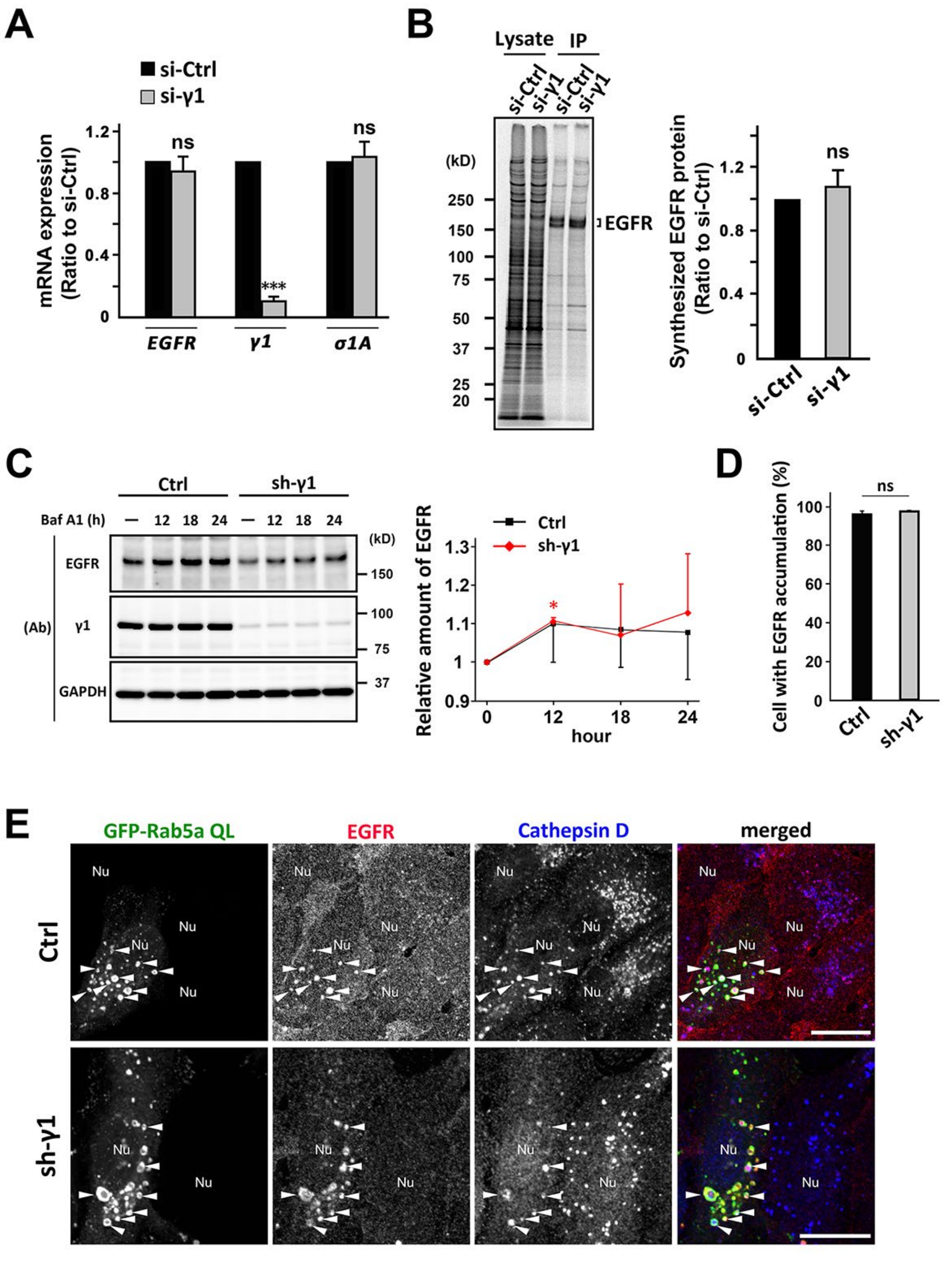

Figure S2 (related to Fig. 2) Synthesis of EGFR was unchanged after AP-1 depletion

(A) qRT-PCR for detection of mRNAs of EGFR, γ1-adaptin, and σ1A-adaptin. ARPE-19 cells transfected with si-Ctrl or si-γ1 for 3 days were used. For each mRNA, the ratio of si-γ1 to si-Ctrl was plotted as the mean ± SD (of three experiments). (B) Anti-EGFR antibody-mediated immunoprecipitation (IP) of ARPE-19 cells transfected with si-Ctrl or si-γ1 and incubated with [^{35}S] methionine/cysteine for 120 min. Approximately 0.5% of the input was applied to the gel (Lysate). The fraction of immunoprecipitated EGFR in the total labeled protein in si-γ1 treated cells was calculated, normalized to that in si-Ctrl treated cells, and then plotted on the right as the mean ± SD (of three experiments). Statistical differences were analyzed using Student's *t*-test (***: $P < 0.001$; ns: not significant). (C) Control (Ctrl) and AP-1-depleted (sh-γ1) ARPE-19 cells were treated with 100 nM bafilomycin A1 (Baf A1) for the indicated amount of time, and then lysed for Western blotting with indicated antibodies. The value of each time point was normalized to that at 0 h chase, and plotted as the mean ± SD (of three biological repeats). Statistical differences between value at each time point and that at 0 h chase were analyzed using Student's *t*-test (*: $P < 0.05$). (D) Control and AP-1-depleted (sh-γ1) ARPE-19 cells transfected with GFP-Rab5a QL were fixed for immunofluorescence microscopy with anti-EGFR antibody. Percentage of cells showing EGFR accumulation over those expressing GFP-Rab5a QL were plotted as the mean ± SD (of three biological repeats). Statistical differences between Ctrl and sh-γ1 were analyzed using Student's t-test (ns: not significant). (E) Double immunofluorescence microscopy of Ctrl and sh-γ1 cells transfected with GFP-Rab5a QL, and immunostained with anti-EGFR (red) and anti-Cathepsin D (blue) antibodies. Arrowheads indicate colocalization of three signals. Nu: nucleus, Bars, 20 μm.

Supplementary information

Supplementary Figure S3 (related to Fig. 4)

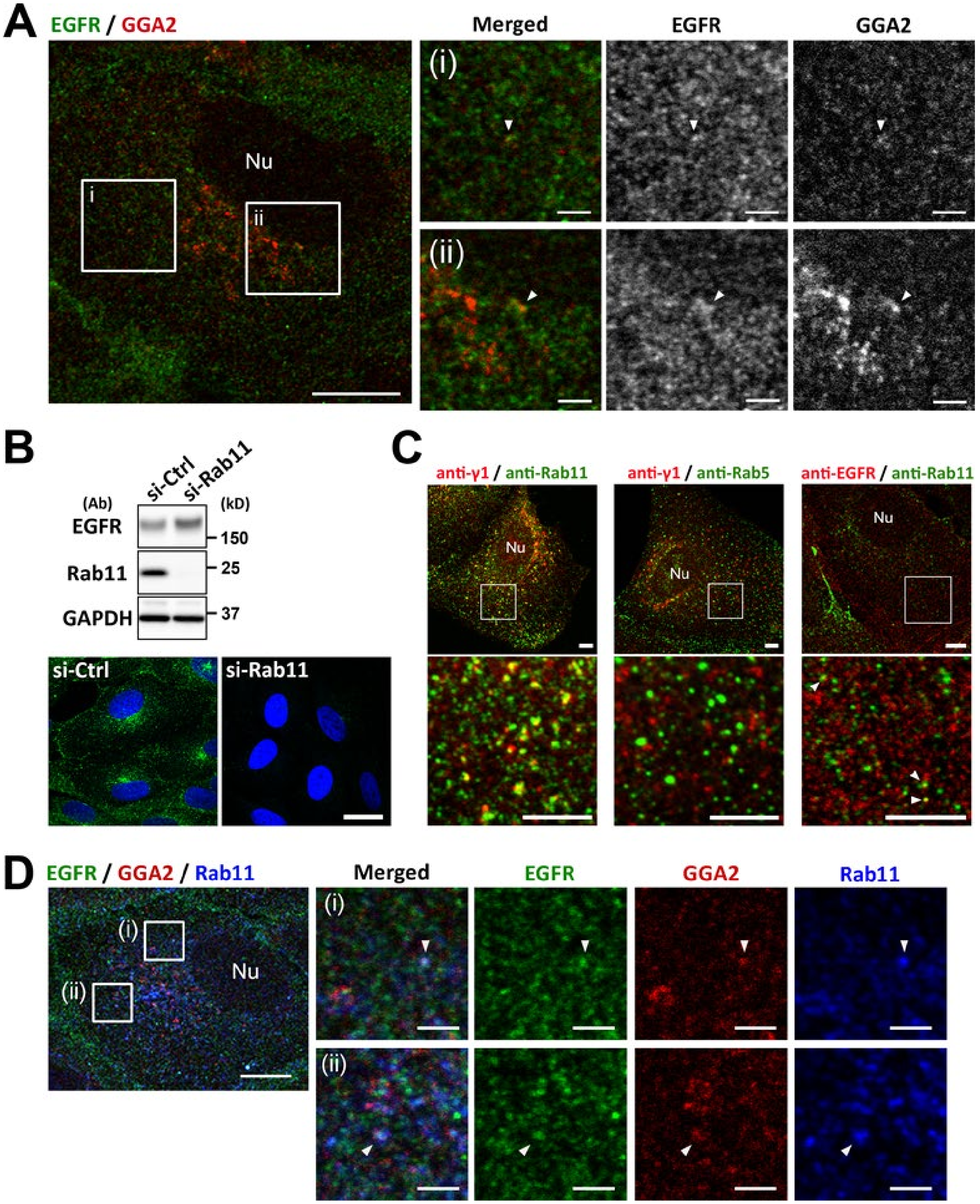

Figure S3 (related to Fig. 4) GGA2 colocalizes with EGFR at Rab-11-positive recycling endosomes

(A) Double immunofluorescence microscopy of ARPE-19 cells immunostained with anti-EGFR (green) and anti-GGA2 (red) antibodies. Boxed regions (i) and (ii) are magnified and shown in the right. Arrowheads indicate colocalization of both signals. Nu: nucleus, Bars: 10 μm (left) and 2 μm (magnified images). (B) Evaluation of anti-Rab11 antibody by Western blotting (upper panel) and immunostaining (lower panel) of ARPE-19 cells that were transfected with siRNA for control (si-Ctrl) or a mix of si-Rab11a and si-Rab11b. EGFR was also analyzed in this analysis. Nuclei were stained with Hoechst 33342 (blue). Bar, 20 μm. (C) ARPE-19 cells immunostained with a combination of anti-γ1-adaptin and anti-Rab11 antibodies, anti-γ1-adaptin and anti-Rab5 antibodies, or anti-EGFR and Rab11 antibodies. Boxed regions are magnified and shown in the bottom. Colocalization of both signals is indicated by arrowheads only for the combination of EGFR and Rab11. Nu: nucleus, Bars, 5 μm. (D) Triple immunofluorescence microscopy of ARPE-19 cells immunostained with anti-EGFR (green), anti-GGA2 (red), and anti-Rab11 (blue) antibodies. Arrowheads indicate colocalization of three signals. Nu: nucleus, Bars, 10 μm (left) and 2 μm (magnified images).

Supplementary information

Supplementary Figure S4 (related to Fig. 4)

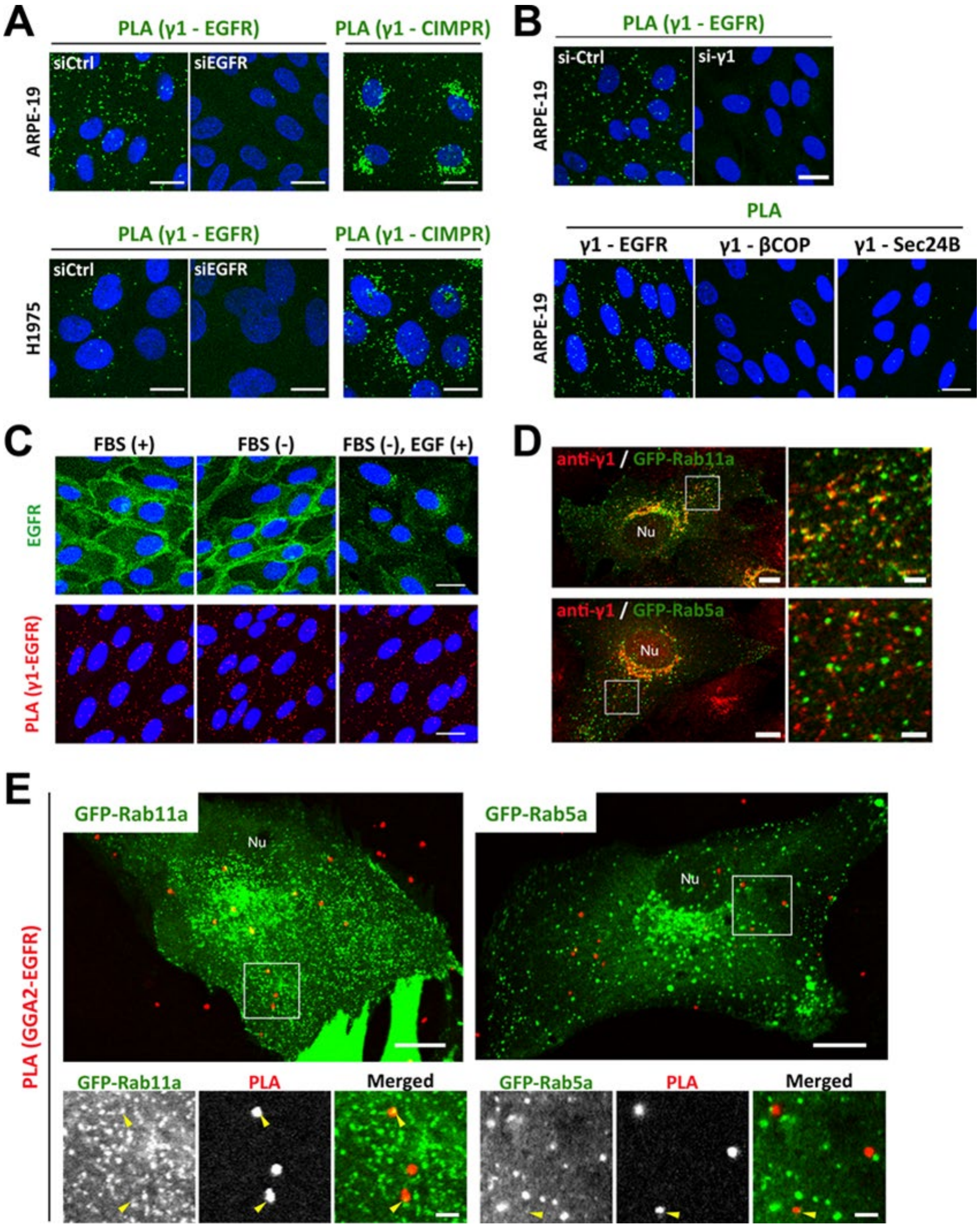

Figure S4 (related to Fig. 4) AP-1 and GGA2 interact with EGFR at the recycling endosomes

(A) PLA of ARPE-19 or H1975 cells transfected with si-Ctrl or si-EGFR. Transfectants were processed for PLA with a combination of anti-γ1-adaptin and anti-EGFR antibodies (γ1–EGFR), or anti-γ1-adaptin and anti-CIMPR (γ1–CIMPR) antibodies (green). Nuclei were stained with DAPI (blue). Bars, 20 µm. (B) Wild type (lower panel) or siRNA-transfected ARPE-19 cells (upper panel) were processed for PLA with a combination of anti-γ1-adaptin and anti-EGFR antibodies (γ1–EGFR), anti-γ1-adaptin and anti-βCOP antibodies (γ1–βCOP), or anti-γ1-adaptin and anti-Sec24B (γ1–Sec24B) antibodies (green). Nuclei were stained with DAPI (blue). Bars, 20 µm. (C) ARPE-19 cells were incubated with DMEM with (+) or without (-) 10 % FBS for 24 h, and treated with (+) or without 10 nM EGF for 10 min. They were fixed for immunostaining with anti-EGFR antibody (upper panel), or PLA using anti-γ1-adaptin and anti-EGFR antibodies (γ1–EGFR; lower panel). Bars, 20 µm. (D) ARPE-19 cells transfected with GFP-Rab11a or GFP-Rab5a (green) were processed for immunofluorescence microscopy using anti-γ1-adaptin antibodies (red). Boxed regions are magnified and shown on the right. Nu: nucleus, Bars, 10 µm (left) and 2 µm (right). (E) PLA of ARPE-19 cells transfected with GFP-Rab11a or GFP-Rab5a (green). Transfectants were processed for PLA with a combination of anti-GGA2 and anti-EGFR (GGA2–EGFR) antibodies (red). Boxed regions are magnified and shown below. Yellow arrowheads indicate the overlap of both signals. Nu: nucleus, Bars, 10 µm (Top) and 2 µm (bottom).

Supplementary information

Supplementary Figure S5 (related to Fig. 5)

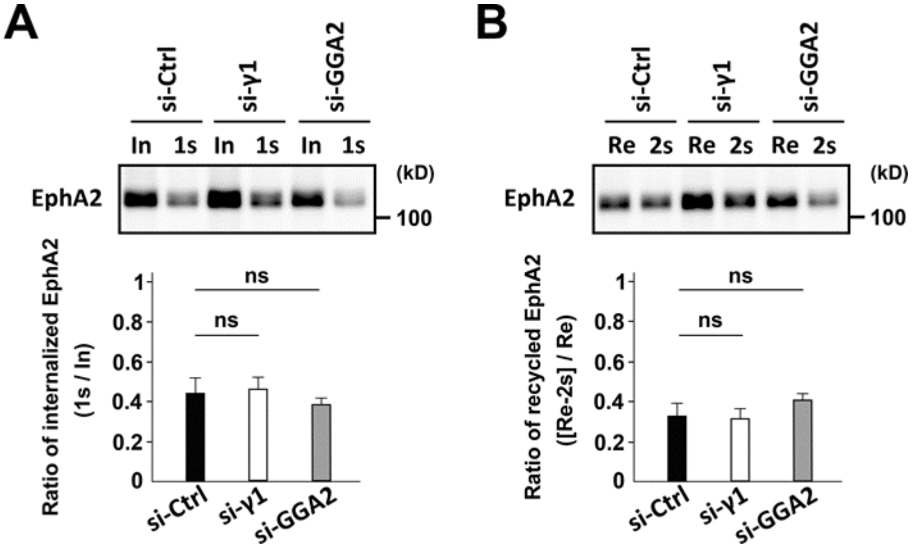

Figure S5 (related to Fig. 5) Depletion of AP-1 or GGA2 does not affect the endosomal recycling of EphA2

ARPE-19 cells transfected with si-Ctrl, si-γ1, or si-GGA2 were treated as described in the Materials and Methods or legend of main Fig 5. After "internalization (In)", "1st stripping (1s), "recycling (Re)", and "2nd stripping (2s), cell lysates were prepared, and biotinylated proteins were collected using avidin-agarose, which were examined by western blotting using anti-EphA2 antibody. For internalization assay, a ratio of the biotinylated EphA2 for "1s" to that for "In" was calculated, and plotted as the mean ± SD of three experiments (A). For recycling assay, the value obtained by subtracting "2s" from "Re" was considered as the EGFR that recycled back to the PM. The ratio of this value to "Re" was calculated and plotted as the mean ± SD of four experiments (B). Statistical differences between the si-Ctrl and si-γ1 or si-GGA2 were analyzed using Student's t-test (ns: not significant).

Supplementary information

Supplementary Figure S6 (related to Fig. 7)

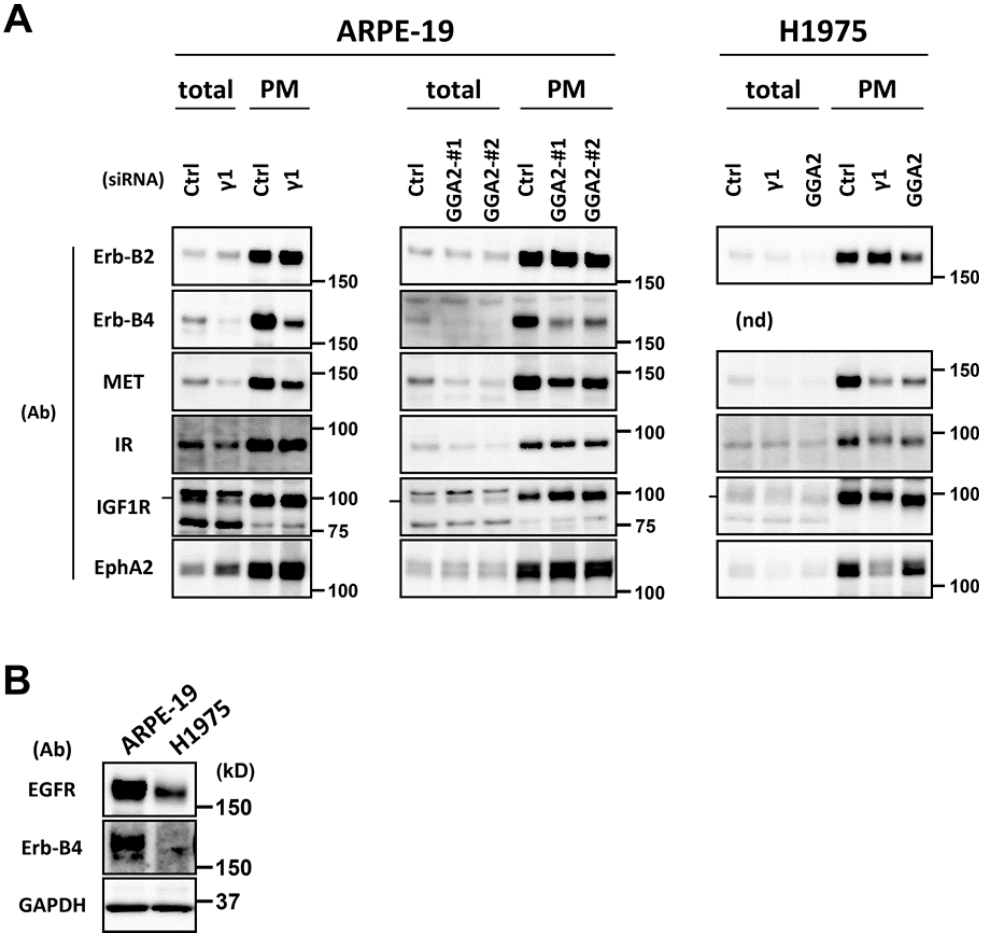

Figure S6 (related to Fig. 7) Depletion of AP-1 or GGA2 causes downregulation of other RTK proteins

(A) Western blotting of ARPE-19 and H1975 cells transfected with siRNA for control (Ctrl), γ1-adaptin (γ1), or GGA2 (GGA2-#1 or -#2). After biotinylation of surface proteins, lysates of the transfectants (total) and the PM fraction (PM) were analyzed using antibodies (Ab) against RTKs (Erb-B2, Erb-B4, MET, IR, IGF1R, and EphA2). Ten % of the input was applied in the total lane.

(B) Western blotting of ARPE-19 and H1975 cells using antibodies (Ab) against EGFR, Erb-B4, or GAPDH.

Supplementary information

Supplementary Figure S7 (related to Fig. 8)

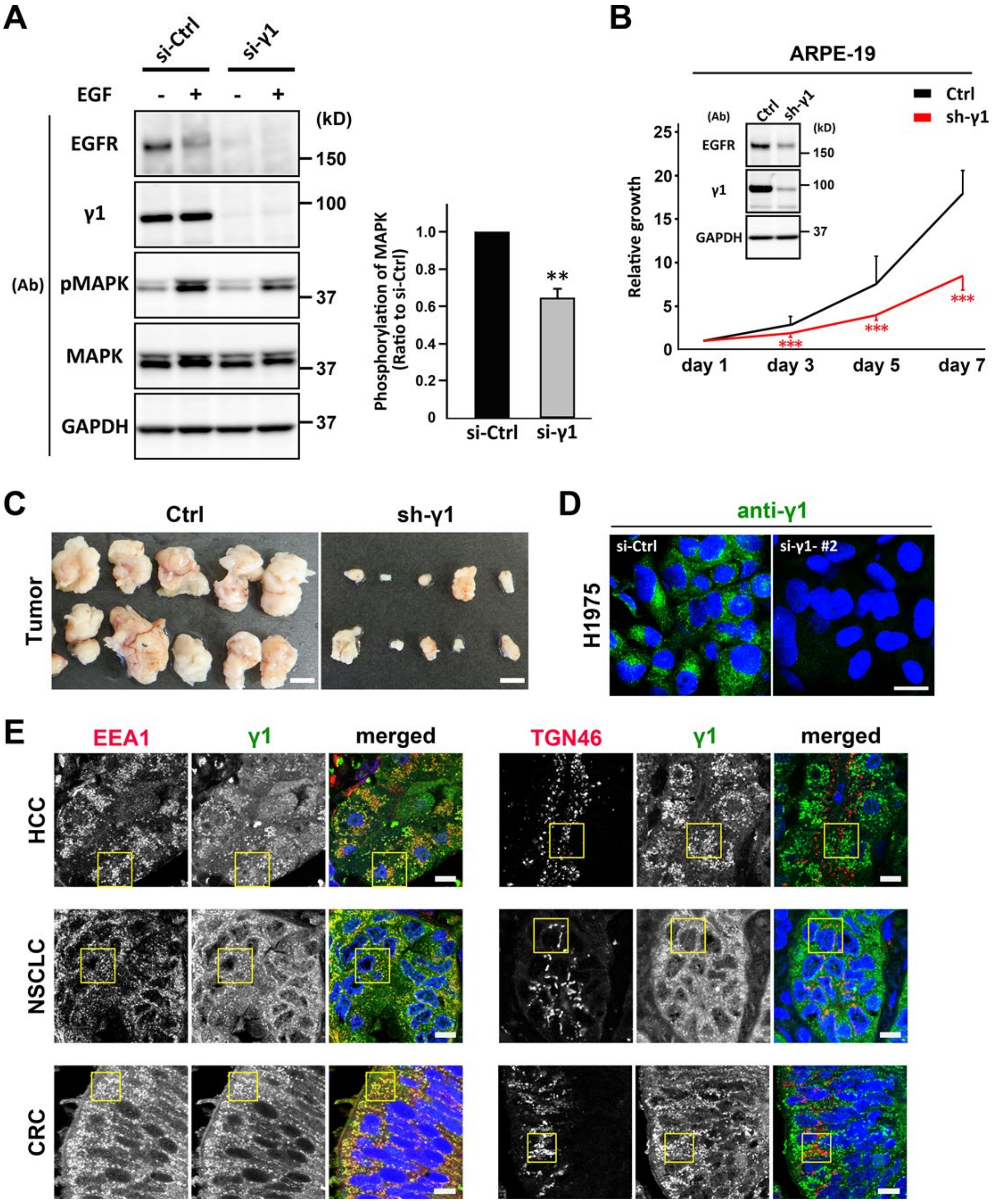

Figure S7 (related to Fig. 8)

AP-1 supports cell growth and is expressed at high levels in endosomes of some human cancer tissues

(A) Serum-starved control (si-Ctrl)- or γ1-adaptin-depleted (si-γ1) ARPE-19 cells were treated with (+) or without (−) 10 nM EGF for 10 min, and were then lysed for Western blot analyses with antibodies (Ab) against EGFR, γ1, phosphorylated MAPK (pMAPK), MAPK, or GAPDH. Increase in the ratio of pMAPK to MAPK after EGF stimulation was calculated and normalized to the value of si-Ctrl. Data were plotted as the mean ± SD of three experiments. Statistical difference between the si-Ctrl and si-γ1 was identified using Student's t-test (**$P < 0.01$). (B) ARPE-19 cells stably expressing sh-γ1 or vector (Ctrl) were subjected to cell proliferation assays. Depletion of γ1-adaptin and EGFR was confirmed by Western blotting using the indicated antibodies (Ab). Data are plotted as the mean ± SD of three experiments. Statistical differences between Ctrl and sh-γ1 at each time point were analyzed using Student's *t*-test (***: $P < 0.001$). (C) Control (Ctrl) or γ1-depleted (sh-γ1) H1975 cells were transplanted into nude mice. Tumors were excised at 4 weeks after transplantation. Data of tumor growth were shown in Fig. 8B. Bars 1cm. (D) Immunofluorescence microscopy in H1975 cell pellets. H1975 cells treated with siRNA for control (si-Ctrl) or γ1-adaptin (si-γ1-#2) were paraffin-embedded and immunostained using anti-γ1 antibodies (green). Nuclei were stained with Hoechst 33342 (blue). Bar, 20 µm. (E) Double-immunostaining of paraffin sections of tumors from hepatocellular carcinoma (HCC, grade 1-2), non-small-cell lung carcinoma (NSCLC, grade 1-2), and colorectal adenocarcinoma (CRC, grade 2); a combination of anti-γ1-adaptin (γ1) and either anti-EEA1 or -TGN46 antibodies was used. Boxed regions are magnified and shown in Fig. 8E. Bars, 10 µm.

Supplementary information

Supplementary Table S1 (related to Fig. 1A)

IP-MS data for EGFR-associated protein (highlighted proteins are listed in Fig. 1A)

Supplementary Table S2 (related to Materials and Methods, Fig. 8C-E, and Supplementary Fig. S7E)

Detailed information of human samples
