# Supplementary material for: Clathrin adapters AP-1 and GGA2 support expression of epidermal growth factor receptor for cell growth
Source: Oncogenesis. 2021 Nov 19;10(11):80. doi: 10.1038/s41389-021-00367-2 (PMC8604998; doi:10.1038/s41389-021-00367-2)
Supplement: Supplementary file 2 — Supplementary Table 1 [file 41389_2021_367_MOESM2_ESM.pdf]

Supplementary Table S1. IP-MS data for EGFR-associated protein (highlighted proteins are listed in Fig. 1A)

Protein FDR Confidence: Combined	Accession	Description	Number of Peptides	MW [kDa]	Gene Symbol	Abundance Ratio: (anti EGFR mouse) / (Normal mouse IgG)	Abundance Ratio Adj. P-Value: (anti EGFR mouse) / (Normal	Abundance（CNT)Normal mouse IgG	Abundance（CNT) anti EGFR mouse
High	NP_002464.1	myosin-9 [Homo sapiens]	190	226.4	MYH9	100	4.4E-17	86,871,914	10,642,589,845
High	NP_005219.2	epidermal growth factor receptor isoform a precursor [Homo sapiens]	83	134.2	EGFR	100	4.4E-17	730,300	2,305,740,885
High	NP_001123630.1	unconventional myosin-Ib isoform 1 [Homo sapiens]	67	131.9	MYO1B	100	4.4E-17	1,629,402	1,284,348,628
High	AAI11753.1	PPP1R12A protein [Homo sapiens]	60	115.2	PPP1R12A	100	4.4E-17	3,162,877	1,147,166,421
High	XP_005248779.1	PREDICTED: unconventional myosin-VI isoform X4 [Homo sapiens]	69	147.6	MYO6	100	4.4E-17	8,337,706	850,546,911
High	AAH65937.1	5'-nucleotidase, ecto (CD73) [Homo sapiens]	31	63.3	NT5E	100	4.4E-17	1,584,668	763,370,232
High	XP_011522182.1	PREDICTED: myosin-10 isoform X3 [Homo sapiens]	123	232.2	MYH10	100	4.4E-17	1,571,189	662,700,486
High	BAG58132.1	unnamed protein product [Homo sapiens]	47	118.5	SPECC1	100	4.4E-17	2,663,362	643,441,247
High	AAQ02673.1	long myosin light chain kinase [Homo sapiens]	63	210.6	MYLK	100	4.4E-17	2,490,516	482,657,057
High	CAH10609.1	hypothetical protein [Homo sapiens]	52	124.5	SPECC1L	100	4.4E-17	706,091	469,638,347
High	XP_016883301.1	PREDICTED: protein GNAS isoform X1 [Homo sapiens]	17	111	GNAS	100	4.4E-17	554,959	340,856,172
High	BAF83860.1	unnamed protein product [Homo sapiens]	9	40.3	GPRC5A	100	4.4E-17	571,948	320,719,035
High	NP_056009.1	unconventional myosin-Id isoform 1 [Homo sapiens]	49	116.1	MYO1D	100	4.4E-17	938,570	302,476,606
High	NP_570603.2	AP-2 complex subunit alpha-1 isoform 2 [Homo sapiens]	45	105.3	AP2A1	100	4.4E-17	2,600,700	294,918,043
High	BAG60181.1	unnamed protein product [Homo sapiens]	19	52.3	AP2M1	100	4.4E-17	1,772,829	253,316,150
High	XP_011522063.1	PREDICTED: myosin phosphatase Rho-interacting protein isoform X1 [Homo sapiens	80	277.4	MPRIP	100	4.4E-17	135,341	242,897,734
High	BAD92985.1	spectrin, beta, non-erythrocytic 1 isoform 1 variant, partial [Homo sapiens]	54	276	SPTBN1	100	4.4E-17	1,115,998	199,573,267
High	BAD93097.1	spectrin, alpha, non-erythrocytic 1 (alpha-fodrin) variant, partial [Homo sapiens]	66	288	SPTAN1	100	4.4E-17	1,768,058	195,163,687
High	AAF78783.1	myosin 5c [Homo sapiens]	55	202.7	MYO5C	100	4.4E-17	1,057,143	191,282,754
High	XP_011520054.2	PREDICTED: uveal autoantigen with coiled-coil domains and ankyrin repeats isoform	47	164.9	UACA	100	4.4E-17	1,027,823	186,749,704
High	AAA52556.1	guanine nucleotide-binding regulatory protein alpha-inhibitory subunit [Homo sapi	22	40.4	GNAI2	100	4.4E-17	1,375,030	182,248,770
High	EAW77638.1	lactamase, beta, isoform CRA_b [Homo sapiens]	17	60.7	LACTB	100	4.4E-17	1,354,316	157,999,288
High	NP_036333.2	Golgi apparatus protein 1 isoform 1 precursor [Homo sapiens]	36	137.1	GLG1	100	4.4E-17	1,413,205	145,014,436
High	NP_006487.1	guanine nucleotide-binding protein G(k) subunit alpha [Homo sapiens]	18	40.5	GNAI3	100	4.4E-17	95,606	127,069,401
High	BAG06714.1	MYO5B variant protein [Homo sapiens]	40	213.6	MYO5B	100	4.4E-17	933,552	124,909,296
High	XP_011532748.1	PREDICTED: drebrin isoform X1 [Homo sapiens]	13	76.4	DBN1	100	4.4E-17	372,803	122,723,436
High	NP_004989.2	unconventional myosin-Ie [Homo sapiens]	29	127	MYO1E	100	4.4E-17	402,335	117,112,014
High	NP_001317099.1	flotillin-2 isoform 2 [Homo sapiens]	20	47.1	FLOT2	100	4.4E-17	687,965	116,385,959
High	EAX00350.1	son of sevenless homolog 1 (Drosophila), isoform CRA_a [Homo sapiens]	33	154.9	SOS1	100	4.4E-17	709,431	115,017,241
High	NP_002636.2	phosphatidylinositol 4-phosphate 3-kinase C2 domain-containing subunit alpha isof	37	190.6	PIK3C2A	100	4.4E-17	635,757	112,167,514
High	BAG35501.1	unnamed protein product [Homo sapiens]	16	37.3	GNB1	100	4.4E-17	427,561	111,797,069
High	AAH68013.1	Myosin IC [Homo sapiens]	74	117.9	MYO1C	100	4.4E-17	307,401	101,640,248
High	NP_127492.1	general transcription factor II-I isoform 1 [Homo sapiens]	26	112.3	GTF2I	100	4.4E-17	522,773	95,558,440
High	EAX03325.1	flotillin 1, isoform CRA_a [Homo sapiens]	19	47.5	FLOT1	100	4.4E-17	800,036	94,780,437
High	XP_011522496.1	PREDICTED: brain-specific angiogenesis inhibitor 1-associated protein 2 isoform X1	15	63	BAIAP2	100	4.4E-17	847,567	94,467,226
High	BAG36767.1	unnamed protein product [Homo sapiens]	17	60.8	YES1	100	4.4E-17	542,272	91,604,950
High	BAD93151.1	Galectin-8 variant, partial [Homo sapiens]	12	38.2	LGALS8	100	4.4E-17	140,623	90,503,915
High	NP_009217.3	synaptopodin isoform A [Homo sapiens]	16	96.3	SYNPO	100	4.4E-17	525,280	81,750,138
High	AAH26326.1	Guanine nucleotide binding protein (G protein), alpha inhibiting activity polypeptide	17	40.3	GNAI1	100	4.4E-17	#NUM!	81,220,154
High	EAW75014.1	calpain 5, isoform CRA_a [Homo sapiens]	19	77.4	CAPN5	100	4.4E-17	77,043	77,302,276
High	NP_006308.3	brain acid soluble protein 1 [Homo sapiens]	11	22.7	BASP1	100	4.4E-17	#NUM!	76,876,219
High	EAW47484.1	myeloid/lymphoid or mixed-lineage leukemia (trithorax homolog, Drosophila); trans	15	207.7	MLLT4; AF	100	4.4E-17	547,485	73,298,382
High	NP_005919.2	lactadherin isoform a preproprotein [Homo sapiens]	10	43.1	MFGE8	100	4.4E-17	96,800	64,165,920
High	AAQ63176.1	myosin phosphatase-Rho interacting protein [Homo sapiens]	74	116.3	MPRIP	100	4.4E-17	#NUM!	64,134,262
High	EAW60315.1	ribosomal protein L3, isoform CRA_f [Homo sapiens]	12	48.8	RPL3	100	4.4E-17	341,587	61,982,263
High	EAW61746.1	platelet-derived growth factor receptor, beta polypeptide, isoform CRA_a [Homo sa	13	124	PDGFRB	100	4.4E-17	253,281	59,534,324
High	BAA32466.2	MEGF5, partial [Homo sapiens]	20	171.3	SLIT3	100	4.4E-17	244,875	57,847,481
High	EAW94708.1	calcium binding and coiled-coil domain 2, isoform CRA_b [Homo sapiens]	16	55.1	CALCOCO:	100	4.4E-17	107,180	53,809,231
High	XP_006710865.2	PREDICTED: ATP-binding cassette sub-family D member 3 isoform X1 [Homo sapien:	12	78.4	ABCD3	100	4.4E-17	268,064	53,559,106
High	NP_009049.2	triple functional domain protein [Homo sapiens]	3	346.7	TRIO	100	4.4E-17	320,220	52,143,845
High	NP_000959.2	60S ribosomal protein L4 [Homo sapiens]	15	47.7	RPL4	100	4.4E-17	407,932	49,972,566
High	CAA38139.1	glypican [Homo sapiens]	13	61.6	GPC1	100	4.4E-17	110,024	48,928,086
High	AAI14430.1	Eukaryotic translation initiation factor 3, subunit A [Homo sapiens]	19	166.4	EIF3A	100	4.4E-17	56,838	41,056,817
High	XP_011518907.1	PREDICTED: epidermal growth factor receptor kinase substrate 8 isoform X1 [Homo	13	93.9	EPS8	100	4.4E-17	#NUM!	38,400,055
High	XP_005253288.1	PREDICTED: CD44 antigen isoform X1 [Homo sapiens]	9	81.6	CD44	100	4.4E-17	118,685	36,929,325
High	NP_056210.1	tectonin beta-propeller repeat-containing protein 1 [Homo sapiens]	11	129.6	TECPR1	100	4.4E-17	205,280	36,579,346
High	NP_937895.1	gelsolin isoform b [Homo sapiens]	31	80.6	GSN	100	4.4E-17	164,378	36,381,624
High	EAW65726.1	hCG2013210, isoform CRA_a [Homo sapiens]	18	153		100	4.4E-17	156,504	33,940,562
High	NP_056344.2	DBH-like monooxygenase protein 1 precursor [Homo sapiens]	9	69.6	MOXD1	100	4.4E-17	121,340	33,687,191
High	AAA58624.1	guanine nucleotide-binding regulatory protein [Homo sapiens]	11	42.1	GNA11	100	4.4E-17	217,831	33,651,199
High	NP_665736.1	GDNF family receptor alpha-1 isoform b precursor [Homo sapiens]	6	50.8	GFRA1	100	4.4E-17	233,035	31,925,989
High	NP_001138994.1	ankycorbin isoform b [Homo sapiens]	75	106.8	RAI14	100	4.4E-17	113,501	31,836,348
High	XP_016874257.1	PREDICTED: oxysterol-binding protein-related protein 8 isoform X4 [Homo sapiens]	11	103.9	OSBPL8	100	4.4E-17	292,800	31,756,376
High	BAA92636.2	KIAA1398 protein, partial [Homo sapiens]	14	170.1	RRBP1	100	4.4E-17	50,255	28,237,403
High	XP_005256518.1	PREDICTED: TOM1-like protein 2 isoform X1 [Homo sapiens]	12	58.7	TOM1L2	100	4.4E-17	115,072	28,180,965
High	AAG27093.1	leucine-zipper protein FKSG13 [Homo sapiens]	9	43.4	PTRF; CAV	100	4.4E-17	223,139	27,606,678
High	BAG35452.1	unnamed protein product [Homo sapiens]	6	39.6	RP2	100	4.4E-17	147,557	27,529,451
High	AAH59394.1	LYN protein, partial [Homo sapiens]	11	65.8	LYN	100	4.4E-17	184,651	25,891,141
High	AAC31899.1	glypican 4 [Homo sapiens]	12	62.4	GPC4	100	4.4E-17	96,467	23,494,609
High	NP_060077.1	protein phosphatase 1 regulatory subunit 12C isoform a [Homo sapiens]	13	84.8	PPP1R12C	100	4.4E-17	187,500	23,059,960
High	EAW93902.1	Tax1 (human T-cell leukemia virus type I) binding protein 1, isoform CRA_b [Homo s	8	97.8	TAX1BP1	100	4.4E-17	201,812	23,039,271
High	AAD27720.1	CGI-11 protein [Homo sapiens]	7	55.8	ATP6V1H	100	4.4E-17	141,575	21,926,741
High	NP_998839.1	tropomyosin beta chain isoform Tpm2.1sm/cy [Homo sapiens]	18	33	TPM2	100	4.4E-17	93,670	21,352,759
High	NP_005757.1	FERM, RhoGEF and pleckstrin domain-containing protein 1 isoform 1 [Homo sapien	10	118.6	FARP1	100	4.4E-17	153,119	20,711,905
High	AAC39793.1	exportin t [Homo sapiens]	12	109.9	XPOT	100	4.4E-17	134,884	20,538,507
High	AAB06875.1	G alpha-q [Homo sapiens]	10	42.1	GNAQ	100	4.4E-17	#NUM!	19,799,287
High	NP_004256.1	fatty acid desaturase 2 isoform 1 [Homo sapiens]	5	52.2	FADS2	100	4.4E-17	157,065	19,709,187
High	AAH30828.1	EGF-like repeats and discoidin I-like domains 3 [Homo sapiens]	5	53.8	EDIL3	100	4.4E-17	#NUM!	18,739,749
High	XP_012359777.1	PREDICTED: ras-related protein R-Ras2 isoform X1 [Nomascus leucogenys]	6	24.2	LOC10060	100	4.4E-17	39,500	18,732,605
High	EAW55991.1	disabled homolog 2, mitogen-responsive phosphoprotein (Drosophila), isoform CRA	6	83.4	DAB2	100	4.4E-17	159,758	17,449,125
High	NP_003349.1	ceramide glucosyltransferase [Homo sapiens]	4	44.8	UGCG	100	4.4E-17	71,607	17,239,712
High	EAW68592.1	importin 7, isoform CRA_b [Homo sapiens]	6	120.9	IPO7	100	4.4E-17	84,165	16,385,821
High	BAH13305.1	unnamed protein product [Homo sapiens]	4	44.6	ATP6V0D1	100	4.4E-17	122,087	16,149,900

High	BAF85342.1	unnamed protein product [Homo sapiens]	7	106.4	RECK	100	4.4E-17	#NUM!	15,414,907
High	AAA35922.1	G protein beta subunit [Homo sapiens]	17	37.3	GNB2	100	4.4E-17	39,862	15,219,140
High	BAD96297.1	eukaryotic translation initiation factor 2, subunit 3 gamma, 52kDa variant, partial [H	5	51.1	EIF2S3	100	4.4E-17	135,434	14,913,734
High	BAD92613.1	SATB family member 2 variant, partial [Homo sapiens]	1	85.5	SATB2	100	4.4E-17	33,565	14,908,135
High	AAF44722.1	novel retinal pigment epithelial cell protein [Homo sapiens]	75	110	RAI14	100	4.4E-17	#NUM!	14,380,937
High	NP_001164014.1	serine/threonine-protein phosphatase PGAM5, mitochondrial isoform 1 [Homo sap	5	32	PGAM5	100	4.4E-17	137,889	14,320,983
High	CAA09590.2	sphingosine-1-phosphate lyase [Homo sapiens]	7	63.5	SGPL1	100	4.4E-17	104,491	12,979,764
High	AAC50064.1	human type 3 inositol 1,4,5-trisphosphate receptor [Homo sapiens]	7	303.9	ITPR3	100	4.4E-17	70,107	12,380,243
High	XP_011539561.1	PREDICTED: acid sphingomyelinase-like phosphodiesterase 3b isoform X1 [Homo sa	6	53.7	SMPDL3B	100	4.4E-17	#NUM!	11,707,448
High	XP_005257832.1	PREDICTED: leucine-rich repeat-containing protein 46 isoform X1 [Homo sapiens]	1	36.5	LRRC46	100	4.4E-17	#NUM!	10,599,359
High	AAM15531.1	182kDa tankyrase1-binding protein [Homo sapiens]	5	181.7	TNKS1BP1	100	4.4E-17	61,647	10,594,292
High	EAW70485.1	myosin, light polypeptide 1, alkali; skeletal, fast, isoform CRA_c [Homo sapiens]	4	21.8	MYL1	100	4.4E-17	#NUM!	9,990,114
High	NP_001017992.1	beta-actin-like protein 2 [Homo sapiens]	12	42	ACTBL2	100	4.4E-17	58,113	9,527,730
High	XP_011513184.1	PREDICTED: epithelial discoidin domain-containing receptor 1 isoform X1 [Homo sa	2	103.6	DDR1	100	4.4E-17	36,002	9,348,205
High	NP_006752.1	14-3-3 protein epsilon [Homo sapiens]	5	29.2	YWHAE	100	4.4E-17	#NUM!	9,087,450
High	XP_011511152.1	PREDICTED: melanotransferrin isoform X1 [Homo sapiens]	5	83.1	MFI2; MEI	100	4.4E-17	#NUM!	8,870,819
High	XP_016859587.1	PREDICTED: echinoderm microtubule-associated protein-like 6 isoform X1 [Homo sa	1	218.6	EML6	100	4.4E-17	#NUM!	8,507,259
High	AAC50796.1	epidermal growth factor receptor, partial [Homo sapiens]	2	5.1	EGFR	100	4.4E-17	41,507	8,494,931
High	EAW57798.1	copine VIII, isoform CRA_a [Homo sapiens]	6	69.3	CPNE8	100	4.4E-17	#NUM!	8,491,884
High	EAW67961.1	protein kinase C and casein kinase substrate in neurons 3, isoform CRA_b [Homo sa	4	48.5	PACSIN3	100	4.4E-17	#NUM!	8,173,153
High	XP_011508832.1	PREDICTED: formin-like protein 2 isoform X1 [Homo sapiens]	5	124.8	FMNL2	100	4.4E-17	58,109	7,944,515
High	EAW85124.1	hCG2042874, isoform CRA_a, partial [Homo sapiens]	1	96.7		100	4.4E-17	#NUM!	7,385,752
High	NP_001091.1	actin, alpha skeletal muscle [Homo sapiens]	23	42	ACTA1	100	4.4E-17	#NUM!	7,206,586
High	BAH12463.1	unnamed protein product [Homo sapiens]	2	119.2	PTK7	100	4.4E-17	29,019	6,957,019
High	XP_005259622.1	PREDICTED: paralemmin-1 isoform X1 [Homo sapiens]	3	42.3	PALM	100	4.4E-17	27,428	6,921,782
High	CAD97961.1	hypothetical protein, partial [Homo sapiens]	2	38.8	NEGR1	100	4.4E-17	32,098	6,899,738
High	EAW91381.1	leiomodin 1 (smooth muscle), isoform CRA_b [Homo sapiens]	4	70.3	LMOD1	100	4.4E-17	#NUM!	6,356,444
High	XP_011515432.1	PREDICTED: plectin isoform X2 [Homo sapiens]	249	516.4	PLEC	100	4.4E-17	#NUM!	6,004,187
High	XP_004769437.1	PREDICTED: calcium/calmodulin-dependent protein kinase type II subunit gamma is	7	65.2	CAMK2G	100	4.4E-17	#NUM!	5,700,563
High	AAH69224.1	CCNY protein, partial [Homo sapiens]	4	39.5	CCNY	100	4.4E-17	#NUM!	5,584,559
High	EAW99216.1	reticulocalbin 2, EF-hand calcium binding domain [Homo sapiens]	2	42.3	RCN2	100	4.4E-17	#NUM!	5,378,169
High	XP_011515669.1	PREDICTED: protein LYRIC isoform X1 [Homo sapiens]	2	66.9	MTDH	100	4.4E-17	#NUM!	5,055,786
High	NP_003886.3	synaptojanin-1 isoform a [Homo sapiens]	3	177.4	SYNJ1	100	4.4E-17	#NUM!	4,928,039
High	NP_001121700.2	taperin [Homo sapiens]	2	75.5	TPRN	100	4.4E-17	43,087	4,901,853
High	EAW80159.1	likely ortholog of mouse schlafen 5 [Homo sapiens]	2	112.1	SLFN5	100	4.4E-17	#NUM!	4,886,966
High	NP_112214.2	NUAK family SNF1-like kinase 2 [Homo sapiens]	3	73.9	NUAK2	100	4.4E-17	#NUM!	4,874,087
High	AAD27650.1	beclin 1 [Homo sapiens]	1	51.9	BECN1	100	4.4E-17	#NUM!	4,872,664
High	NP_037418.3	putative RNA-binding protein 15B [Homo sapiens]	1	97.1	RBM15B	100	4.4E-17	#NUM!	4,744,949
High	NP_064536.2	stAR-related lipid transfer protein 7, mitochondrial precursor [Homo sapiens]	1	43.1	STARD7	100	4.4E-17	#NUM!	4,600,737
High	EAW60869.1	contactin associated protein 1, isoform CRA_b [Homo sapiens]	1	156.1	CNTNAP1	100	4.4E-17	#NUM!	4,331,310
High	NP_689449.1	cdc42 effector protein 1 [Homo sapiens]	2	40.3	CDC42EP1	100	4.4E-17	#NUM!	4,148,955
High	XP_005264475.1	PREDICTED: girdin isoform X1 [Homo sapiens]	1	233	CCDC88A	100	4.4E-17	#NUM!	3,983,857
High	NP_065176.3	neuron navigator 1 isoform 1 [Homo sapiens]	2	202.3	NAV1	100	4.4E-17	35,714	3,962,623
High	AAR28200.1	MHC class I antigen, partial [Homo sapiens]	8	21	HLA-A	100	4.4E-17	#NUM!	3,929,931
High	XP_011510207.1	PREDICTED: lisH domain-containing protein ARMC9 isoform X1 [Homo sapiens]	6	102.4	ARMC9	100	4.4E-17	#NUM!	3,889,691
High	BAF82833.1	unnamed protein product [Homo sapiens]	5	62.7	GPC6	100	4.4E-17	#NUM!	3,836,033
High	AAA92733.1	hydroxymethylglutaryl-CoA lyase [Homo sapiens]	5	34.4	HMGCL	100	4.4E-17	#NUM!	3,669,286
High	NP_612382.1	myeloid-associated differentiation marker [Homo sapiens]	2	35.3	MYADM	100	4.4E-17	#NUM!	3,590,064
High	BAB55076.1	unnamed protein product [Homo sapiens]	1	62.4	EEPD1	100	4.4E-17	#NUM!	3,552,589
High	EAW68192.1	CD59 antigen, complement regulatory protein, isoform CRA_a [Homo sapiens]	1	17.2	CD59	100	4.4E-17	#NUM!	3,545,679
High	ALG00229.1	anti-HIV-1 immunoglobulin light chain variable region, partial [Homo sapiens]	1	11.6		100	4.4E-17	#NUM!	3,390,141
High	XP_016854992.1	PREDICTED: collagen alpha-1(III) chain-like [Homo sapiens]	1	34.9	LOC10272	100	4.4E-17	#NUM!	3,244,228
High	NP_116198.3	mitoguardin 2 isoform 1 [Homo sapiens]	1	72.1	FAM73B; I	100	4.4E-17	#NUM!	3,239,346
High	XP_011521729.1	PREDICTED: RING finger and SPRY domain-containing protein 1 isoform X1 [Homo s	2	67.5	RSPRY1	100	4.4E-17	#NUM!	3,216,063
High	XP_011527866.1	PREDICTED: lanosterol synthase isoform X1 [Homo sapiens]	3	84.3	LSS	100	4.4E-17	#NUM!	3,146,185
High	XP_005274379.1	PREDICTED: phosphatidylinositol-binding clathrin assembly protein isoform X1 [Hor	18	74.3	PICALM	100	4.4E-17	#NUM!	3,081,169
High	XP_005274380.1	PREDICTED: phosphatidylinositol-binding clathrin assembly protein isoform X2 [Hor	18	73.7	PICALM	100	4.4E-17	#NUM!	2,948,990
High	AAA75276.1	This CDS feature is included to show the translation of the corresponding V_segmer	1	5.5		100	4.4E-17	#NUM!	2,909,050
High	XP_016814383.1	PREDICTED: syntenin-1 isoform X1 [Pan troglodytes]	2	34.9	SDCBP	100	4.4E-17	#NUM!	2,906,218
High	AAI46795.1	Discs, large homolog 5 (Drosophila) [Homo sapiens]	2	213.8	DLG5	100	4.4E-17	#NUM!	2,878,270
High	AAH36470.1	Cadherin 2, type 1, N-cadherin (neuronal) [Homo sapiens]	1	99.7	CDH2	100	4.4E-17	#NUM!	2,832,362
High	XP_011511393.1	PREDICTED: helicase-like transcription factor isoform X1 [Homo sapiens]	1	114.3	HLTF	100	4.4E-17	#NUM!	2,809,507
High	BAF85173.1	unnamed protein product [Homo sapiens]	1	44.7	CEP44	100	4.4E-17	#NUM!	2,778,492
High	AAA59010.1	immunoglobulin lambda-chain, partial [Homo sapiens]	1	13.5		100	4.4E-17	#NUM!	2,740,327
High	XP_011530601.1	PREDICTED: ras-related protein Rab-33B isoform X1 [Homo sapiens]	1	30.7	RAB33B	100	4.4E-17	#NUM!	2,708,604
High	AAB94130.1	G protein coupled receptor [Homo sapiens]	1	41.5	ACKR3; CX	100	4.4E-17	#NUM!	2,691,814
High	XP_011517136.1	PREDICTED: serine/threonine-protein phosphatase 2A activator isoform X1 [Homo s	1	50.9	PPP2R4; P	100	4.4E-17	#NUM!	2,680,150
High	AAB84227.1	Cyr61 protein [Homo sapiens]	1	42	CYR61	100	4.4E-17	#NUM!	2,614,009
High	EAW80972.1	actinin, alpha 1, isoform CRA_b, partial [Homo sapiens]	29	55.2	ACTN1	100	4.4E-17	#NUM!	2,607,529
High	AAC62750.2	prion protein precursor [Homo sapiens]	2	30.7	PRNP	100	4.4E-17	21,112	2,526,848
High	EAW83288.1	WW domain containing E3 ubiquitin protein ligase 2, isoform CRA_b [Homo sapiens	2	101.6	WWP2	100	4.4E-17	#NUM!	2,515,904
High	XP_011518702.1	PREDICTED: fatty acyl-CoA reductase 1 isoform X1 [Homo sapiens]	1	59.8	FAR1	100	4.4E-17	#NUM!	2,508,024
High	AAA52383.1	translational initiation factor beta subunit [Homo sapiens]	1	38.4	EIF2S2	100	4.4E-17	#NUM!	2,464,950
High	BAF84958.1	unnamed protein product [Homo sapiens]	2	61.2	CPNE2	100	4.4E-17	#NUM!	2,464,675
High	AAD09340.1	NAD+-specific isocitrate dehydrogenase beta subunit isoform B [Homo sapiens]	1	42.4		100	4.4E-17	#NUM!	2,442,215
High	BAD96489.1	multi-substrate lipid kinase variant, partial [Homo sapiens]	1	47.1	AGK	100	4.4E-17	#NUM!	2,418,744
High	NP_002964.3	ataxin-2 isoform 1 [Homo sapiens]	1	140.2	ATXN2	100	4.4E-17	#NUM!	2,382,012
High	CAA91196.1	plectin [Homo sapiens]	231	531.4	PLEC	100	4.4E-17	#NUM!	2,374,631
High	NP_000970.1	60S ribosomal protein L18 isoform 1 [Homo sapiens]	1	21.6	RPL18	100	4.4E-17	#NUM!	2,334,641
High	BAG35762.1	unnamed protein product [Homo sapiens]	1	48.2	KHDRBS1	100	4.4E-17	#NUM!	2,269,783
High	BAG51355.1	unnamed protein product [Homo sapiens]	1	52.5	DAPK3	100	4.4E-17	#NUM!	2,234,428
High	Q8NCA5.1	RecName: Full=Protein FAM98A	1	55.4	FAM98A	100	4.4E-17	#NUM!	2,228,828
High	NP_001651.1	ADP-ribosylation factor 4 [Homo sapiens]	1	20.5	ARF4	100	4.4E-17	#NUM!	2,220,916
High	BAG70074.1	14-3-3 protein beta/alpha [Homo sapiens]	4	28.1	YWHAB	100	4.4E-17	#NUM!	2,217,548
High	AAC50296.1	band 7.2b stomatin [Homo sapiens]	3	32.6	STOM	100	4.4E-17	#NUM!	2,208,591
High	XP_013963508.1	PREDICTED: BTB/POZ domain-containing adapter for CUL3-mediated RhoA degrada	3	35	KCTD10	100	4.4E-17	#NUM!	2,199,399
High	NP_055807.1	disheveled-associated activator of morphogenesis 1 isoform 1 [Homo sapiens]	2	123.4	DAAM1	100	4.4E-17	#NUM!	2,194,108
High	EAX07509.1	KIAA1522, isoform CRA_a [Homo sapiens]	1	125.8	KIAA1522	100	4.4E-17	#NUM!	2,193,310
High	NP_001005743.1	protein numb homolog isoform 1 [Homo sapiens]	1	70.8	NUMB	100	4.4E-17	#NUM!	2,187,823
High	CAC94258.1	immunoglobulin lambda chain variable region, partial [Homo sapiens]	1	10.4		100	4.4E-17	#NUM!	2,168,522
High	NP_001275880.1	tetratricopeptide repeat protein 7A isoform 1 [Homo sapiens]	1	98.9	TTC7A	100	4.4E-17	#NUM!	2,154,795
High	AAH34346.1	Nucleoporin 93kDa [Homo sapiens]	1	93.5	NUP93	100	4.4E-17	#NUM!	2,121,240
High	AAH16838.1	Carboxypeptidase, vitellogenic-like [Homo sapiens]	1	54.2	CPVL	100	4.4E-17	#NUM!	2,021,875
High	XP_011532966.1	PREDICTED: calnexin isoform X2 [Homo sapiens]	1	73.4	CANX	100	4.4E-17	#NUM!	2,012,214
High	XP_011536806.1	PREDICTED: serine/threonine-protein phosphatase PP1-gamma catalytic subunit isc	13	41.4	PPP1CC	100	4.4E-17	#NUM!	2,008,392

High	AAH05978.1	Karyopherin alpha 2 (RAG cohort 1, importin alpha 1) [Homo sapiens]	1	57.8	KPNA2	100	4.4E-17	#NUM!	2,002,415
High	NP_542194.2	protein SOGA1 isoform 1 [Homo sapiens]	1	183.7	SOGA1	100	4.4E-17	#NUM!	1,995,442
High	XP_011543331.1	PREDICTED: low-density lipoprotein receptor-related protein 5 isoform X1 [Homo sa	2	184.3	LRP5	100	4.4E-17	#NUM!	1,985,230
High	XP_016880484.1	PREDICTED: nucleolar transcription factor 1 isoform X1 [Homo sapiens]	1	91.2	UBTF	100	4.4E-17	#NUM!	1,950,398
High	AAF29413.1	SEL1L [Homo sapiens]	2	88.7	SEL1L	100	4.4E-17	#NUM!	1,934,097
High	AFK93471.1	epidermal growth factor receptor, partial [Homo sapiens]	18	21	EGFR	100	4.4E-17	#NUM!	1,917,276
High	NP_998764.1	anoctamin-5 isoform a [Homo sapiens]	1	107.1	ANO5	100	4.4E-17	#NUM!	1,903,297
High	XP_005265804.1	PREDICTED: exocyst complex component 1 isoform X1 [Homo sapiens]	1	104.1	EXOC1	100	4.4E-17	#NUM!	1,879,632
High	BAG37182.1	unnamed protein product [Homo sapiens]	22	50.4	TUBB3	100	4.4E-17	#NUM!	1,850,907
High	XP_011516800.1	PREDICTED: G-protein-signaling modulator 1 isoform X2 [Homo sapiens]	1	78.2	GPSM1	100	4.4E-17	#NUM!	1,842,343
High	BAG09613.1	ubiquitin conjugation factor E4 A, partial [synthetic construct]	1	123.5		100	4.4E-17	#NUM!	1,840,401
High	BAD38640.1	putative protein product of HKMT1098 [Homo sapiens]	1	67.9	PPP1R18	100	4.4E-17	#NUM!	1,786,332
High	AAC67244.1	metalloprotease 1 [Homo sapiens]	1	117.5	PITRM1	100	4.4E-17	#NUM!	1,777,548
High	BAH13266.1	unnamed protein product [Homo sapiens]	1	125.3	EFTUD1; E	100	4.4E-17	#NUM!	1,769,472
High	AAA61281.2	vimentin, partial [Homo sapiens]	39	41.5	VIM	100	4.4E-17	#NUM!	1,701,324
High	1CP3_A	Chain A, Crystal Structure Of The Complex Of Apopain With The Tetrapeptide Inhibi	1	31.6		100	4.4E-17	#NUM!	1,683,852
High	EAX04100.1	tubulin-specific chaperone c [Homo sapiens]	1	42.4	TBCC	100	4.4E-17	#NUM!	1,662,135
High	XP_011542608.1	PREDICTED: serine/threonine-protein kinase MRCK alpha isoform X1 [Homo sapiens	1	209.7	CDC42BP/	100	4.4E-17	#NUM!	1,662,047
High	BAC78818.1	leukocyte-derived arginine aminopeptidase long form variant [Homo sapiens]	3	110.4	ERAP2	100	4.4E-17	#NUM!	1,649,303
High	AAA82889.1	CENP-F kinetochore protein [Homo sapiens]	1	367.4	CENPF	100	4.4E-17	#NUM!	1,633,231
High	XP_011539620.1	PREDICTED: basement membrane-specific heparan sulfate proteoglycan core protei	3	488.2	HSPG2	100	4.4E-17	#NUM!	1,597,181
High	EAW70081.1	hCG1640974, isoform CRA_b, partial [Homo sapiens]	1	24.1		100	4.4E-17	#NUM!	1,563,853
High	BAG35256.1	unnamed protein product [Homo sapiens]	2	141.3	HDLBP	100	4.4E-17	#NUM!	1,525,553
High	AAI31535.1	DEAH (Asp-Glu-Ala-Asp/His) box polypeptide 57 [Homo sapiens]	1	155.5	DHX57	100	4.4E-17	#NUM!	1,517,746
High	NP_001317958.1	protein phosphatase 1 regulatory subunit 12B isoform h [Homo sapiens]	4	116.4	PPP1R12B	100	4.4E-17	#NUM!	1,472,348
High	EAW88345.1	mannosidase, alpha, class 1B, member 1, isoform CRA_a, partial [Homo sapiens]	1	82.5	MAN1B1	100	4.4E-17	#NUM!	1,463,677
High	AIE48223.1	HIP1-ALK fusion protein [Homo sapiens]	1	141		100	4.4E-17	#NUM!	1,423,245
High	CAB60200.1	2-hydroxyphytanoyl-CoA lyase [Homo sapiens]	1	63.7	HACL1	100	4.4E-17	#NUM!	1,415,642
High	XP_011514682.1	PREDICTED: neurabin-1 isoform X1 [Homo sapiens]	4	156.4	PPP1R9A	100	4.4E-17	#NUM!	1,387,778
High	NP_001887.1	casein kinase II subunit alpha' [Homo sapiens]	1	41.2	CSNK2A2	100	4.4E-17	#NUM!	1,375,691
High	NP_055422.1	UBX domain-containing protein 4 [Homo sapiens]	1	56.7	UBXN4	100	4.4E-17	#NUM!	1,363,586
High	NP_001837.2	collagen alpha-2(IV) chain preproprotein [Homo sapiens]	1	167.4	COL4A2	100	4.4E-17	#NUM!	1,352,152
High	NP_001166925.1	pyruvate dehydrogenase E1 component subunit alpha, somatic form, mitochondria	1	47.5	PDHA1	100	4.4E-17	#NUM!	1,349,503
High	BAH11954.1	unnamed protein product [Homo sapiens]	1	137.2	TJP2	100	4.4E-17	#NUM!	1,267,382
High	XP_008002416.1	PREDICTED: plasma membrane calcium-transporting ATPase 1 isoform X4 [Chloroce	12	138.7	ATP2B1	100	4.4E-17	#NUM!	1,266,192
High	CAA29608.1	unnamed protein product [Homo sapiens]	1	69.9	SRPR; SRP	100	4.4E-17	#NUM!	1,240,280
High	BAA07892.3	KIAA0097 protein [Homo sapiens]	1	225.4	CKAP5	100	4.4E-17	#NUM!	1,169,560
High	ABQ59048.1	CHD1L protein [Homo sapiens]	1	101.3	CHD1L	100	4.4E-17	#NUM!	1,166,993
High	XP_016856104.1	PREDICTED: epidermal growth factor receptor substrate 15 isoform X1 [Homo sapie	1	102.4	EPS15	100	4.4E-17	#NUM!	1,068,515
High	NP_919415.2	vesicle-associated membrane protein-associated protein A isoform 2 [Homo sapien:	3	27.9	VAPA	100	4.4E-17	#NUM!	1,048,737
High	AAD38537.1	transportin-SR [Homo sapiens]	2	109.8	TNPO3	100	4.4E-17	#NUM!	984,857
High	EAW82497.1	tetracycline transporter-like protein, isoform CRA_b [Homo sapiens]	1	66.5	MFSD10	100	4.4E-17	#NUM!	977,347
High	XP_011531792.1	PREDICTED: putative ATP-dependent RNA helicase DHX30 isoform X1 [Homo sapien	2	140.4	DHX30	100	4.4E-17	#NUM!	950,130
High	EAX08933.1	glypican 6, isoform CRA_c [Homo sapiens]	1	14.2	GPC6	100	4.4E-17	#NUM!	935,016
High	NP_001026855.2	apoptosis-stimulating of p53 protein 2 isoform 1 [Homo sapiens]	1	126.2	TP53BP2	100	4.4E-17	#NUM!	890,855
High	BAC11670.1	unnamed protein product [Homo sapiens]	2	24.9	NDFIP1	100	4.4E-17	#NUM!	885,341
High	EAW78642.1	SMC4 structural maintenance of chromosomes 4-like 1 (yeast), isoform CRA_c [Hon	1	147.2	SMC4	100	4.4E-17	#NUM!	869,126
High	NP_149014.3	rho GTPase-activating protein SYDE1 isoform 1 [Homo sapiens]	1	79.7	SYDE1	100	4.4E-17	#NUM!	810,290
High	NP_775746.1	DCN1-like protein 3 [Homo sapiens]	1	34.3	DCUN1D3	100	4.4E-17	#NUM!	750,253
High	NP_000475.1	amyloid beta A4 protein isoform a precursor [Homo sapiens]	1	86.9	APP	100	4.4E-17	#NUM!	747,596
High	XP_016874530.1	PREDICTED: protein MON2 homolog isoform X1 [Homo sapiens]	1	190.4	MON2	100	4.4E-17	#NUM!	730,532
High	NP_068598.1	FAST kinase domain-containing protein 5, mitochondrial [Homo sapiens]	1	86.5	FASTKD5	100	4.4E-17	#NUM!	716,469
High	BAF85119.1	unnamed protein product [Homo sapiens]	1	115.1	BMPR2	100	4.4E-17	#NUM!	641,724
High	NP_036243.1	activator of 90 kDa heat shock protein ATPase homolog 1 isoform 1 [Homo sapiens]	1	38.3	AHSA1	100	4.4E-17	#NUM!	631,019
High	BAH14440.1	unnamed protein product [Homo sapiens]	17	113	ATP1A3	100	4.4E-17	#NUM!	618,483
High	XP_005251987.1	PREDICTED: golgin subfamily A member 2 isoform X1 [Homo sapiens]	1	116	GOLGA2	100	4.4E-17	#NUM!	611,425
High	NP_001333827.1	epidermal growth factor receptor isoform f precursor [Homo sapiens]	78	125.7	EGFR	100	4.4E-17	#NUM!	600,588
High	AAA51585.1	alpha-actinin [Homo sapiens]	12	103.2	ACTN3	100	4.4E-17	#NUM!	573,472
High	NP_001073867.1	proteasome-associated protein ECM29 homolog [Homo sapiens]	1	223.6	KIAA0368	100	4.4E-17	#NUM!	573,187
High	EAW97965.1	acyl-Coenzyme A dehydrogenase family, member 10, isoform CRA_e [Homo sapiens	1	125.6	ACAD10	100	4.4E-17	#NUM!	561,026
High	AAD42744.1	DEAD-box protein [Homo sapiens]	1	92.1	DDX20	100	4.4E-17	#NUM!	533,757
High	XP_016859066.1	PREDICTED: receptor tyrosine-protein kinase erbB-4 isoform X1 [Homo sapiens]	3	151.2	ERBB4	100	4.4E-17	#NUM!	511,855
High	NP_056380.2	E3 ubiquitin-protein ligase listerin isoform 1 [Homo sapiens]	1	205	LTN1	100	4.4E-17	#NUM!	506,386
High	AAH56219.1	DEAH (Asp-Glu-Ala-His) box polypeptide 29 [Homo sapiens]	1	155.2	DHX29	100	4.4E-17	#NUM!	504,093
High	BAG37923.1	unnamed protein product [Homo sapiens]	1	67.6	VPS33A	100	4.4E-17	#NUM!	456,645
High	BAD92778.1	toll interacting protein variant, partial [Homo sapiens]	1	38.9	TOLLIP	100	4.4E-17	#NUM!	440,865
High	NP_056288.2	HEAT repeat-containing protein 5A [Homo sapiens]	1	222.6	HEATR5A	100	4.4E-17	#NUM!	389,962
High	NP_001010867.1	putative transferase CAF17, mitochondrial isoform 1 precursor [Homo sapiens]	1	38.1	IBA57	100	4.4E-17	#NUM!	388,488
High	NP_612357.4	ubiquitin thioesterase otulin [Homo sapiens]	1	40.2	FAM105B	100	4.4E-17	#NUM!	354,188
High	XP_005247411.1	PREDICTED: calcium-transporting ATPase type 2C member 1 isoform X1 [Homo sapi	1	108.1	ATP2C1	100	4.4E-17	#NUM!	294,524
High	AAA17374.1	human homolog of E. coli mutL gene product, Swiss-Prot Accession Number P2336	1	84.5	MLH1	100	4.4E-17	#NUM!	267,137
High	BAG63985.1	unnamed protein product [Homo sapiens]	1	116.9	IPO11	100	4.4E-17	#NUM!	230,015
High	AAC41949.1	interleukin-1 receptor-associated kinase [Homo sapiens]	1	76.5	IRAK1	100	4.4E-17	#NUM!	229,602
High	XP_011519396.1	PREDICTED: UDP-glucose:glycoprotein glucosyltransferase 2 isoform X1 [Homo sapi	3	182.8	UGGT2	100	4.4E-17	#NUM!	227,437
High	EAW68637.1	hCG1784554, isoform CRA_a [Homo sapiens]	3	39.1	EIF3F	100	4.4E-17	#NUM!	210,780
High	AAH50538.1	Radical S-adenosyl methionine domain containing 1 [Homo sapiens]	1	48.7	RSAD1	100	4.4E-17	#NUM!	185,804
High	NP_001098707.1	coronin-1C isoform a [Homo sapiens]	21	58.9	CORO1C	98.204	0.05701	3,900,135	383,009,147
High	NP_002700.1	serine/threonine-protein phosphatase PP1-beta catalytic subunit isoform 1 [Homo :	16	37.2	PPP1CB	97.933	0.05744	610,257	59,764,649
High	XP_005271379.1	PREDICTED: nexilin isoform X1 [Homo sapiens]	2	80.8	NEXN	96.18	0.22233	42,960	4,085,282
High	CAC37685.1	neurabin II protein [Homo sapiens]	10	89.4	PPP1R9B	93.954	0.0643	258,562	24,292,944
High	NP_001934.2	desmoglein-2 preproprotein [Homo sapiens]	23	122.2	DSG2	93.807	0.06456	1,074,411	100,787,234
High	AAX82177.1	actin-like protein, partial [Homo sapiens]	4	11.5	ACTG1	93.068	0.17589	65,319	6,079,149
High	AAI51247.1	DnaJ (Hsp40) homolog, subfamily C, member 13 [synthetic construct]	55	254.3	DNAJC13	91.707	0.06853	1,886,370	172,994,036
High	XP_005003559.1	PREDICTED: clathrin heavy chain 1 isoform X1 [Cavia porcellus]	99	192.3	Cltc; LOC1	91.692	0.06853	39,338,999	3,607,067,344
High	CAI46036.1	hypothetical protein [Homo sapiens]	15	39.5	TMOD3	90.82	0.07035	666,968	60,573,848
High	XP_016859428.1	PREDICTED: trifunctional enzyme subunit beta, mitochondrial isoform X1 [Homo sa	22	52.1	HADHB	89.946	0.07216	1,376,956	123,852,256
High	AAC51917.1	mammary tumor-associated protein INT6 [Homo sapiens]	8	52	EIF3E	89.799	0.07239	178,551	16,033,634
High	AAC27674.1	eIF-3 p110 subunit [Homo sapiens]	8	105.3	EIF3C	89.172	0.07365	152,921	13,636,300
High	BAA03941.1	enoyl-CoA hydratase/3-hydroxyacyl-CoA dehydrogenase alpha-subunit of trifunctio	32	82.9	HADHA	86.861	0.0791	2,439,851	211,926,674
High	BAB71043.1	unnamed protein product [Homo sapiens]	5	52.7	MBOAT7	86.581	0.07963	179,095	15,506,211
High	AAA35682.1	ribosmal protein small subunit [Homo sapiens]	10	30	RPS3A	86.432	0.07993	466,215	40,296,045
High	AAH71611.1	EFR3A protein, partial [Homo sapiens]	21	99.3	EFR3A	85.374	0.08253	966,714	82,532,739
High	AAV51260.1	FINGER5-like variant I [Homo sapiens]	2	29.1	YIPF5	85.336	0.14837	78,146	6,668,663
High	AAH08285.1	Coiled-coil domain containing 102A [Homo sapiens]	12	62.6	CCDC102/	84.994	0.08352	551,956	46,913,052
High	XP_005274279.1	PREDICTED: myelin regulatory factor isoform X1 [Homo sapiens]	7	124.4	C11orf9; N	84.442	0.08478	361,376	30,515,317

High	AAY88753.1	toll-like receptor 6 [Homo sapiens]	1	91.8	TLR6	82.432	0.09002	155,957	12,855,784
High	BAG57380.1	unnamed protein product [Homo sapiens]	3	150.9	PNPLA6	82.048	0.24526	59,729	4,900,625
High	BAF82217.1	unnamed protein product [Homo sapiens]	47	105.6	AP2B1	81.649	0.09206	3,587,592	292,923,706
High	EAX03959.1	BTB (POZ) domain containing 9, isoform CRA_a, partial [Homo sapiens]	3	75.3	BTBD9	81.202	0.1751	78,440	6,339,002
High	XP_006716181.1	PREDICTED: voltage-dependent calcium channel subunit alpha-2/delta-1 isoform X1	21	125.2	CACNA2D	79.042	0.0995	709,721	56,097,813
High	BAE97426.1	decay-accelerating factor splicing variant 5 [Homo sapiens]	8	59	CD55	78.963	0.09951	676,351	53,406,417
High	EAX11430.1	plakophilin 4, isoform CRA_f [Homo sapiens]	8	131.8	PKP4	78.446	0.10066	197,206	15,469,944
High	NP_001074419.1	unconventional myosin-Ic isoform b [Homo sapiens]	74	119.6	MYO1C	77.194	0.27395	66,744	5,144,476
High	NP_695003.1	sorting nexin-33 isoform 1 [Homo sapiens]	2	65.2	SNX33	76.867	0.30837	68,858	4,390,031
High	AAH24282.1	Similar to AFG3 ATPase family gene 3-like 2 (yeast), partial [Homo sapiens]	11	90.1	AFG3L2	76.265	0.1076	236,140	18,009,356
High	NP_006261.1	ras-related protein R-Ras [Homo sapiens]	2	23.5	RRAS	75.42	0.25808	20,034	1,510,994
High	AAA53375.1	protein-tyrosine kinase [Homo sapiens]	9	108.2	EPHA2	74.793	0.11275	769,712	57,569,065
High	BAD96203.1	eukaryotic translation initiation factor 3, subunit 2 beta, 36kDa variant, partial [Hon	3	36.5	EIF3I	73.692	0.18551	107,088	7,891,510
High	XP_011528984.1	PREDICTED: plexin-B2 isoform X1 [Homo sapiens]	26	211.7	PLXNB2	73.422	0.11794	1,147,793	84,273,778
High	AAH39297.1	Fibronectin type III domain containing 3B [Homo sapiens]	2	132.9	FNDC3B	73.039	0.33278	47,219	3,448,833
High	XP_005258516.1	PREDICTED: heterogeneous nuclear ribonucleoprotein U-like protein 1 isoform X1 [I	9	96.8	HNRNPUL	72.184	0.12286	205,508	14,834,384
High	NP_003371.2	vimentin [Homo sapiens]	55	53.6	VIM	71.105	0.12723	23,916,788	1,700,601,815
High	NP_001316559.1	pleckstrin homology domain-containing family A member 7 isoform 1 [Homo sapier	5	144.3	PLEKHA7	70.083	0.34876	66,849	4,684,966
High	AAA61156.1	transglutaminase [Homo sapiens]	13	89.7	TGM1	70.01	0.13207	684,772	47,940,961
High	NP_003744.1	eukaryotic translation initiation factor 3 subunit D [Homo sapiens]	4	63.9	EIF3D	69.784	0.13302	225,569	15,740,833
High	NP_002218.2	tyrosine-protein kinase JAK1 isoform 1 [Homo sapiens]	9	133.2	JAK1	68.224	0.14068	381,356	26,017,481
High	AAH67263.1	Exocyst complex component 4 [Homo sapiens]	2	110.4	EXOC4	67.818	0.36888	50,340	3,394,523
High	BAG72819.1	TBC1 domain family, member 9B, partial [synthetic construct]	3	140.4		67.747	0.30649	81,406	5,514,980
High	NP_116262.2	ubiquitin-associated and SH3 domain-containing protein B [Homo sapiens]	17	72.6	UBASH3B	67.328	0.14459	584,234	39,335,060
High	BAD97009.1	connexin 43 variant, partial [Homo sapiens]	8	42.9	GJA1	66.99	0.14568	375,506	25,155,013
High	CAG30322.1	dJ1014D13.1 [Homo sapiens]	12	70.9	EIF3L	66.809	0.14651	385,031	25,723,665
High	AAK76432.1	SSA1 [Homo sapiens]	23	54.2	TRIM21	66.785	0.14657	2,609,174	174,253,936
High	NP_001247421.1	radixin isoform 1 [Homo sapiens]	13	71	RDX	66.635	0.35917	44,270	2,949,929
High	XP_011511955.1	PREDICTED: LIM and calponin homology domains-containing protein 1 isoform X19	45	125.1	LIMCH1	66.357	0.14837	3,263,122	216,531,440
High	EAW86376.1	inter-alpha (globulin) inhibitor H2, isoform CRA_a [Homo sapiens]	3	106.5	ITIH2	66.186	0.14879	201,589	12,008,754
High	EAW86447.1	tubulin, alpha-like 3 [Homo sapiens]	5	49.9	TUBAL3	65.942	0.31692	82,030	5,409,191
High	NP_001020262.1	ATP-binding cassette sub-family F member 1 isoform a [Homo sapiens]	5	95.9	ABCF1	63.498	0.18255	135,218	8,586,084
High	EAW68685.1	tripeptidyl peptidase I, isoform CRA_a [Homo sapiens]	2	62.2	TPP1	63.3	0.25114	121,132	7,647,275
High	AAI53882.1	GCN1 general control of amino-acid synthesis 1-like 1 (yeast) [Homo sapiens]	7	292.6	GCN1L1; (	63.051	0.40481	71,084	4,481,909
High	NP_009027.1	clathrin light chain A isoform b [Homo sapiens]	1	27.1	CLTA	62.711	0.41401	63,010	3,951,431
High	BAG35427.1	unnamed protein product [Homo sapiens]	7	105.8	PSMD1	62.35	0.17027	369,226	23,021,293
High	AAC50259.1	encodes region of fatty acid synthase activity; FAS; multifunctional protein [Homo s	70	272.9	FASN	61.903	0.39231	47,928	2,964,162
High	XP_006721929.1	PREDICTED: integrin beta-4 isoform X1 [Homo sapiens]	12	211.6	ITGB4	61.65	0.17399	308,112	18,995,137
High	BAF84382.1	unnamed protein product [Homo sapiens]	2	97.6	COPG1	61.234	0.17627	497,758	30,479,946
High	CAG38772.1	G3BP [Homo sapiens]	6	52.1	G3BP1	60.327	0.181	346,866	20,925,270
High	NP_001348.2	ATP-dependent RNA helicase A [Homo sapiens]	9	140.9	DHX9	60.19	0.18187	336,758	20,269,450
High	EAW88064.1	ribosomal protein L7a, isoform CRA_d, partial [Homo sapiens]	3	35	RPL7A	59.839	0.31828	103,592	6,198,847
High	AAH14546.1	ARP2 actin-related protein 2 homolog (yeast) [Homo sapiens]	5	44.7	ACTR2	59.478	0.29169	106,230	6,318,383
High	NP_036437.1	AP-2 complex subunit alpha-2 isoform 2 [Homo sapiens]	36	103.9	AP2A2	59.278	0.18746	1,651,360	97,889,520
High	NP_079426.2	threonine--tRNA ligase, mitochondrial isoform a [Homo sapiens]	5	81	TARS2	58.889	0.23353	136,216	8,021,648
High	XP_011515270.2	PREDICTED: protein argonaute-2 isoform X1 [Homo sapiens]	1	104	AGO2; EIF	58.846	0.39743	22,773	1,327,457
High	XP_011511727.1	PREDICTED: cyclin-G-associated kinase isoform X1 [Homo sapiens]	8	147.7	GAK	58.593	0.20722	148,693	8,712,362
High	NP_060024.2	poly [ADP-ribose] polymerase 14 [Homo sapiens]	5	202.7	PARP14	58.45	0.45479	70,280	3,856,934
High	NP_008996.1	hsp90 co-chaperone Cdc37 [Homo sapiens]	8	44.4	CDC37	58.023	0.19568	304,006	17,639,288
High	XP_008974847.1	PREDICTED: plasma membrane calcium-transporting ATPase 4 isoform X6 [Pan pani	25	137.8	ATP2B4	57.3	0.20114	458,042	26,245,870
High	XP_011541156.1	PREDICTED: protein NPAT isoform X1 [Homo sapiens]	1	155.3	NPAT	56.96	0.17756	189,949	10,819,428
High	EAX07023.1	ribosomal protein S8, isoform CRA_a, partial [Homo sapiens]	4	27.4	RPS8	56.371	0.21651	177,474	10,004,400
High	CAA52777.1	protein-tyrosine kinase [Homo sapiens]	3	96.7	DDR2	55.937	0.33676	124,047	6,938,820
High	XP_005546531.1	PREDICTED: fragile X mental retardation syndrome-related protein 1 isoform X1 [Ma	11	76.2	LOC10192	55.833	0.21203	315,016	17,588,302
High	NP_001894.2	catenin alpha-1 isoform 1 [Homo sapiens]	21	100	CTNNA1	55.684	0.21267	402,765	22,427,751
High	CAD38787.1	hypothetical protein, partial [Homo sapiens]	8	113	MATN2	55.67	0.21267	348,936	19,425,234
High	ACD03459.1	plakophilin-2 transcript variant 2b [Homo sapiens]	10	97.3	PKP2	55.581	0.21267	531,798	29,557,888
High	AAA58617.1	granulin [Homo sapiens]	1	63.5	GRN	55.404	0.41021	27,246	1,481,276
High	EAW61187.1	basigin (Ok blood group), isoform CRA_g [Homo sapiens]	4	46.4	BSG	55.055	0.21651	335,330	18,461,685
High	EAW71815.1	ribosomal protein S4, X-linked, isoform CRA_a [Homo sapiens]	7	43.4	RPS4X	55.016	0.2167	352,644	19,400,923
High	AAB66581.1	Skb1Hs [Homo sapiens]	5	72.7	PRMT5	54.984	0.21674	2,862,024	157,364,709
High	BAG61467.1	unnamed protein product [Homo sapiens]	5	44.1	NAP1L4	54.442	0.47769	84,944	4,624,559
High	XP_016866762.1	PREDICTED: protein DEK isoform X1 [Homo sapiens]	5	43.2	DEK	54.391	0.22158	228,010	12,401,654
High	XP_008974720.1	PREDICTED: probable arginine--tRNA ligase, mitochondrial isoform X1 [Pan paniscus	2	66.6	RARS2	52.822	0.46246	44,849	2,359,129
High	AAC50804.1	epidermal growth factor receptor, partial [Homo sapiens]	1	5	EGFR	52.471	0.23902	252,638	13,256,089
High	EAW64487.1	leucine rich repeat (in FLII) interacting protein 2, isoform CRA_b [Homo sapiens]	11	84.1	LRRFIP2	51.292	0.24941	414,553	21,263,429
High	EAW95034.1	leucine zipper protein 1, isoform CRA_a [Homo sapiens]	1	120.2	LUZP1	51.195	0.50026	55,593	2,744,953
High	NP_001333694.1	unconventional myosin-XVIIIa isoform c [Homo sapiens]	57	233.1	MYO18A	50.344	0.25808	326,220	16,423,329
High	NP_002261.3	transportin-1 isoform 1 [Homo sapiens]	4	102.3	TNPO1	50.127	0.28056	275,976	9,508,215
High	AAH44952.1	Virus-induced signaling adapter [Homo sapiens]	1	56.6	MAVS	49.859	0.4613	29,494	1,470,558
High	NP_001182484.1	clathrin interactor 1 isoform 1 [Homo sapiens]	12	70.3	CLINT1	48.685	0.27577	872,374	42,471,793
High	NP_115584.1	anthrax toxin receptor 1 isoform 1 precursor [Homo sapiens]	2	62.7	ANTXR1	48.565	0.51896	96,137	4,687,373
High	XP_016879224.1	PREDICTED: large neutral amino acids transporter small subunit 1 isoform X1 [Homo	1	54.9	SLC7A5	48.034	0.54013	65,392	3,149,978
High	XP_005249816.1	PREDICTED: 2-oxoglutarate dehydrogenase, mitochondrial isoform X1 [Homo sapier	26	117.6	OGDH	47.487	0.28875	1,841,813	87,461,846
High	EAW57794.1	hCG1773636 [Homo sapiens]	4	20.5		47.047	0.52938	103,814	4,684,740
High	XP_011745923.1	PREDICTED: regulator of nonsense transcripts 1 isoform X1 [Macaca nemestrina]	10	125.1	UPF1	46.288	0.30279	291,399	13,488,336
High	EAW58315.1	chromosome 9 open reading frame 19, isoform CRA_a, partial [Homo sapiens]	1	37.8	GLIPR2	45.985	0.55907	67,103	2,744,937
High	XP_016874541.1	PREDICTED: ELKS/Rab6-interacting/CAST family member 1 isoform X1 [Homo sapier	10	131	ERC1	45.857	0.30692	375,288	17,209,669
High	EAW61633.1	La ribonucleoprotein domain family, member 1, isoform CRA_b, partial [Homo sapie	5	129.2	LARP1	45.843	0.4304	154,641	7,089,159
High	XP_016878726.1	PREDICTED: multidrug resistance-associated protein 1 isoform X1 [Homo sapiens]	4	173.8	ABCC1	45.602	0.58057	81,641	3,636,034
High	AAB59629.1	dihydrolipoamide succinyltransferase [Homo sapiens]	11	48.6	DLST	44.456	0.32512	2,013,601	89,516,970
High	NP_005889.3	caprin-1 isoform 1 [Homo sapiens]	8	78.3	CAPRIN1	44.332	0.32645	605,427	26,839,938
High	BAG36500.1	unnamed protein product [Homo sapiens]	2	87.2	TAP1	43.888	0.53419	35,947	1,590,922
High	AAH58938.1	LZTS2 protein [Homo sapiens]	2	72.7	LZTS2	43.792	0.564	39,481	1,766,937
High	AAD29951.1	myosin heavy chain IIx/d [Homo sapiens]	18	223	MYH1	43.735	0.33396	751,117	32,850,389
High	AAA36377.2	NOTCH 2 [Homo sapiens]	9	265.2	NOTCH2	43.701	0.33399	294,336	12,862,667
High	EAL24246.1	deafness, autosomal dominant 5 [Homo sapiens]	7	54.5	DFNA5; G:	43.12	0.34168	852,845	36,774,297
High	NP_000685.1	aldehyde dehydrogenase family 3 member B1 isoform a [Homo sapiens]	6	51.8	ALDH3B1	42.807	0.34665	432,632	18,519,885
High	AAH19222.1	PA2G4 protein, partial [Homo sapiens]	3	45.1	PA2G4	42.672	0.55641	137,676	5,874,874
High	BAD92747.1	EBNA-2 co-activator variant, partial [Homo sapiens]	14	107.4	SND1	42.353	0.35246	346,141	14,660,128
High	BAG11373.1	sperm-specific antigen 2, partial [synthetic construct]	5	138.3		42.351	0.35246	273,734	11,592,908
High	AAI44510.1	RICTOR protein [Homo sapiens]	3	194.9	RICTOR	42.315	0.60583	51,839	2,193,574
High	NP_002943.2	40S ribosomal protein S2 [Homo sapiens]	6	31.3	RPS2	41.749	0.36086	761,980	31,811,951
High	XP_005272732.1	PREDICTED: probable ubiquitin carboxyl-terminal hydrolase FAF-X isoform X1 [Hom	2	292.7	USP9X	41.599	0.62138	107,319	4,464,407
High	AAB42010.1	Prt1 homolog [Homo sapiens]	11	98.8	EIF3B	41.086	0.37022	637,516	26,193,161

High	EAW97346.1	PRKC, apoptosis, WT1, regulator, isoform CRA_a [Homo sapiens]	11	36.6	PAWR	40.938	0.37146	1,272,763	52,104,340
High	BAG64064.1	unnamed protein product [Homo sapiens]	3	110.2	EIF4G2	40.833	0.63703	83,596	3,411,867
High	EAW63626.1	lysyl oxidase-like 2, isoform CRA_b [Homo sapiens]	7	94.7	LOXL2	40.527	0.37909	258,754	10,486,572
High	AAA52458.1	FMR1, partial [Homo sapiens]	5	75	FMR1	40.171	0.36415	265,693	10,673,168
High	XP_011531200.1	PREDICTED: cysteine-rich motor neuron 1 protein isoform X7 [Homo sapiens]	4	118.3	CRIM1	40.062	0.64106	107,615	4,311,216
High	A0FGR8.1	RecName: Full=Extended synaptotagmin-2; Short=E-Syt2; AltName: Full=Chr2Syt	2	102.3	ESYT2	39.961	0.6079	130,424	5,211,848
High	AAI36662.1	Rho guanine nucleotide exchange factor (GEF) 5 [Homo sapiens]	6	176.5	ARHGEF5	39.749	0.6056	145,709	5,791,737
High	AAG36783.1	MLEL1 protein [Homo sapiens]	1	114.7	DHX36	39.627	0.63245	64,476	2,530,707
High	CAA79696.1	contactin [Homo sapiens]	6	113.3	CNTN1	38.826	0.43186	235,782	9,011,921
High	NP_008835.5	DNA-dependent protein kinase catalytic subunit isoform 1 [Homo sapiens]	48	468.8	PRKDC	38.804	0.40767	4,286,800	166,344,322
High	NP_001186600.1	calumenin isoform c precursor [Homo sapiens]	4	38	CALU	38.716	0.48527	202,606	7,844,090
High	AAK07558.1	B aggressive lymphoma long isoform [Homo sapiens]	20	96.2	PARP9	38.693	0.40929	1,542,522	59,685,297
High	EAW73550.1	hypothetical protein BC002942, isoform CRA_c [Homo sapiens]	3	79.7	LMF2	38.51	0.66547	108,103	4,163,105
High	XP_011544451.1	PREDICTED: mitochondrial glutamate carrier 2 isoform X1 [Homo sapiens]	2	44	SLC25A18	38.261	0.43881	233,883	8,948,565
High	BAH13296.1	unnamed protein product [Homo sapiens]	4	65.6	ANXA11	38.106	0.41728	302,338	11,520,811
High	XP_016868908.1	PREDICTED: aspartyl/asparaginyl beta-hydroxylase isoform X1 [Homo sapiens]	15	90.4	ASPH	37.961	0.41782	916,803	34,802,426
High	XP_011511221.1	PREDICTED: 5'-3' exoribonuclease 1 isoform X1 [Homo sapiens]	1	194.1	XRN1	37.947	0.60292	25,314	964,029
High	NP_001243764.1	queuine tRNA-ribosyltransferase accessory subunit 2 isoform 2 [Homo sapiens]	1	48.2	QTRTD1; (	37.873	0.63714	124,241	4,705,352
High	AAH03618.1	TRIOBP protein [Homo sapiens]	22	68	TRIOBP	37.843	0.41949	2,157,587	81,649,759
High	AAG02026.1	hematopoietic PBX-interacting protein [Homo sapiens]	4	80.7	PBXIP1	37.605	0.43881	231,015	8,687,310
High	XP_016878025.1	PREDICTED: tropomyosin alpha-1 chain isoform X3 [Homo sapiens]	16	45	TPM1	37.586	0.42451	770,080	28,943,942
High	NP_000108.1	emerin [Homo sapiens]	5	29	EMD	37.267	0.4304	325,154	12,117,389
High	XP_011528148.1	PREDICTED: protein kinase C and casein kinase substrate in neurons protein 2 isofor	5	55.9	PACSIN2	37.139	0.43194	436,979	16,229,119
High	BAG11121.1	ubiquitin-protein ligase E3C, partial [synthetic construct]	5	123.9		37.021	0.44446	236,893	8,770,021
High	EAW79476.1	deltex 3-like (Drosophila) [Homo sapiens]	13	83.5	DTX3L	36.999	0.43467	1,196,840	44,281,544
High	CAD97853.1	hypothetical protein [Homo sapiens]	2	116.8	OGT	36.941	0.43575	1,677,194	61,956,698
High	XP_011517275.1	PREDICTED: TNF receptor-associated factor 2 isoform X1 [Homo sapiens]	3	61.9	TRAF2	36.582	0.67608	69,344	2,536,747
High	XP_005269750.1	PREDICTED: myoferlin isoform X1 [Homo sapiens]	20	236.5	MYOF	36.551	0.43923	1,211,336	44,276,041
High	BAH13251.1	unnamed protein product [Homo sapiens]	1	95.3	EXOC6	36.505	0.564	18,043	658,877
High	NP_001304889.1	5'-3' exoribonuclease 2 isoform 1 [Homo sapiens]	2	116.7	XRN2	36.151	0.65003	47,129	1,703,767
High	BAD93140.1	LL5 beta protein variant, partial [Homo sapiens]	23	142.7	PHLDB2	35.991	0.44993	2,296,438	82,650,934
High	XP_003279937.1	PREDICTED: protein transport protein Sec61 subunit alpha isoform 1 isoform X1 [Nc	6	52.9	SEC61A1	35.668	0.45648	720,355	25,693,363
High	EAW70740.1	desmin, isoform CRA_b [Homo sapiens]	5	24.2	DES	35.601	0.45652	281,472	10,020,632
High	BAG38011.1	unnamed protein product [Homo sapiens]	3	47.9	TES	35.357	0.70311	103,261	3,503,852
High	XP_016884318.1	PREDICTED: phosphatidylinositol 4-kinase alpha isoform X1 [Homo sapiens]	23	237.3	PI4KA	35.086	0.46674	1,074,231	37,690,831
High	BAA02656.1	DnaJ protein homolog [Homo sapiens]	9	44.8	DNAJA1	34.918	0.47072	934,325	32,624,334
High	XP_011533736.1	PREDICTED: collagen alpha-1(XII) chain isoform X1 [Homo sapiens]	11	339.4	COL12A1	34.882	0.47118	552,141	19,259,721
High	XP_011508441.1	PREDICTED: rho guanine nucleotide exchange factor 2 isoform X1 [Homo sapiens]	3	127.7	ARHGEF2	34.757	0.71995	111,908	3,940,036
High	XP_005268061.1	PREDICTED: inverted formin-2 isoform X1 [Homo sapiens]	3	139	INF2	34.136	0.69566	71,068	2,425,964
High	XP_006722489.1	PREDICTED: E3 ubiquitin-protein ligase NEDD4-like isoform X9 [Homo sapiens]	4	116.8	NEDD4L	33.999	0.70311	85,727	2,914,573
High	BAA23486.1	polyubiquitin [Homo sapiens]	5	68.4	UBC	33.907	0.48993	16,392,830	555,824,496
High	ABB18377.1	transferrin receptor trafficking protein [Homo sapiens]	2	107.4	SH3BP4	33.817	0.72053	123,599	4,179,755
High	XP_016882419.1	PREDICTED: microtubule-associated protein 1S isoform X1 [Homo sapiens]	3	113.1	MAP1S	33.793	0.6898	144,557	4,884,934
High	EAW69331.1	nicalin homolog (zebrafish), isoform CRA_b [Homo sapiens]	3	63.5	NCLN	33.711	0.60251	228,031	7,687,126
High	P39060.5	RecName: Full=Collagen alpha-1(XVIII) chain; Contains: RecName: Full=Endostatin; I	11	178.1	COL18A1	33.403	0.50163	1,186,061	39,618,350
High	NP_001544.1	insulin-like growth factor-binding protein 7 isoform 1 precursor [Homo sapiens]	1	29.1	IGFBP7	33.369	0.72967	129,351	4,316,342
High	EAW91377.1	importin 9, isoform CRA_c, partial [Homo sapiens]	17	117	IPO9	33.35	0.50263	1,485,242	49,532,824
High	NP_001018077.1	plasminogen activator inhibitor 1 RNA-binding protein isoform 1 [Homo sapiens]	6	44.9	SERBP1	33.343	0.50263	677,908	22,603,715
High	AAF06354.1	melanoma-associated antigen MG50, partial [Homo sapiens]	4	167.1	PXDN	33.325	0.68943	177,963	5,930,617
High	AAD51932.1	RNA-binding protein isoform G3BP-2a [Homo sapiens]	4	54.1	G3BP2	33.191	0.70774	73,100	2,418,726
High	NP_037368.1	ataxin-10 isoform 1 [Homo sapiens]	9	53.5	ATXN10	33.087	0.50688	304,125	10,062,690
High	NP_001165131.1	NADH-cytochrome b5 reductase 3 isoform 3 [Homo sapiens]	1	38.2	CYB5R3	32.863	0.69031	161,755	5,315,780
High	NP_036439.2	band 4.1-like protein 3 isoform 1 [Homo sapiens]	7	120.6	EPB41L3	32.623	0.5284	301,306	9,829,499
High	AAI21148.1	Translocase of inner mitochondrial membrane 50 homolog (S. cerevisiae) [Homo sa	4	50.4	TIMM50	32.616	0.67608	185,341	6,045,160
High	NP_588614.1	1-phosphatidylinositol 4,5-bisphosphate phosphodiesterase delta-3 [Homo sapiens]	17	89.2	PLCD3	32.606	0.51945	2,331,327	76,015,499
High	CAG29359.1	GRB2, partial [Homo sapiens]	6	25.1	GRB2	32.398	0.52454	479,406	15,531,747
High	AAA58643.1	gamma-glutamyl carboxylase [Homo sapiens]	1	87.5	GGCX	32.017	0.70552	62,441	1,998,699
High	NP_057368.3	CCR4-NOT transcription complex subunit 1 isoform a [Homo sapiens]	9	266.8	CNOT1	31.61	0.53878	378,415	11,946,354
High	AAI14621.1	Periplakin [Homo sapiens]	16	204.5	PPL	31.55	0.54013	899,852	28,390,265
High	XP_016869212.1	PREDICTED: 60S ribosomal protein L8 isoform X1 [Homo sapiens]	3	31.7	RPL8	31.448	0.54265	480,907	15,123,735
High	CAA65633.1	mitochondrial citrate transport protein, partial [Homo sapiens]	6	34.8	SLC25A1	31.234	0.54647	381,496	11,915,841
High	AAF29085.1	HSPC121 [Homo sapiens]	4	44.4	PTPLAD1;	30.994	0.55309	453,296	14,049,474
High	BAG70078.1	catenin beta-1 [Homo sapiens]	19	85.5	CTNNB1	30.881	0.55572	1,253,039	38,695,133
High	BAD96324.1	ribosomal protein L5 variant, partial [Homo sapiens]	11	34.3	RPL5	30.695	0.55907	407,772	12,516,452
High	EAW56188.1	ATPase family, AAA domain containing 3A, isoform CRA_c [Homo sapiens]	15	68.9	ATAD3A	30.534	0.56319	2,093,051	63,909,806
High	NP_000773.2	1,25-dihydroxyvitamin D(3) 24-hydroxylase, mitochondrial isoform 1 precursor [Hoi	3	58.8	CYP24A1	29.994	0.77112	109,980	3,298,796
High	NP_001244066.1	E3 ubiquitin-protein ligase Itchy homolog isoform 1 [Homo sapiens]	4	102.7	ITCH	29.943	0.68901	236,920	7,093,993
High	BAG10210.1	breakpoint cluster region protein, partial [synthetic construct]	1	142.7		29.933	0.765	88,992	2,663,802
High	EAW95471.1	ubiquitin specific peptidase 10, isoform CRA_b [Homo sapiens]	2	98	USP10	29.751	0.77182	113,213	3,370,944
High	AAQ89432.1	mannosyltransferase, partial [Homo sapiens]	1	53.1	ALG1	29.657	0.73967	58,496	1,734,805
High	BAG50990.1	unnamed protein product [Homo sapiens]	3	32.4	ATG5	29.58	0.76834	155,626	4,603,395
High	NP_003357.2	cytochrome b-c1 complex subunit 2, mitochondrial precursor [Homo sapiens]	5	48.4	UQCRC2	29.529	0.61519	279,649	8,257,658
High	XP_005260495.1	PREDICTED: 1-phosphatidylinositol 4,5-bisphosphate phosphodiesterase gamma-1 i	3	152.7	PLCG1	29.472	0.77648	133,066	3,921,780
High	NP_115914.1	tubulin beta-6 chain isoform 1 [Homo sapiens]	27	49.8	TUBB6	29.209	0.5969	1,658,670	48,448,104
High	XP_016864546.1	PREDICTED: casein kinase I isoform X1 [Homo sapiens]	1	55.2	CSNK1G3	29.153	0.79033	127,580	3,721,163
High	EAW59051.1	hCG28765, isoform CRA_b [Homo sapiens]	7	147.1	PALM2-AK	29.114	0.5985	430,810	12,542,745
High	BAG35769.1	unnamed protein product [Homo sapiens]	1	88.8	ZW10	28.993	0.76641	76,424	2,215,736
High	AAC05086.1	delta7-sterol reductase [Homo sapiens]	5	54.5	DHCR7	28.853	0.604	1,269,110	36,617,212
High	BAD92940.1	Dihydrolipoamide dehydrogenase, variant, partial [Homo sapiens]	13	55.6	DLD	28.829	0.60433	2,764,253	79,690,731
High	AAK17926.1	tropomyosin 4-anaplastic lymphoma kinase fusion protein, partial [Homo sapiens]	12	36.6		28.808	0.60441	477,244	13,748,603
High	EAW47668.1	sorting nexin 9, isoform CRA_a [Homo sapiens]	11	67.4	SNX9	28.785	0.60464	2,068,509	59,542,822
High	AAH11855.1	DnaJ (Hsp40) homolog, subfamily A, member 3 [Homo sapiens]	3	52.5	DNAJA3	28.535	0.755	195,180	5,569,405
High	NP_001034764.1	protein unc-45 homolog A isoform 3 [Homo sapiens]	6	101.6	UNC45A	28.498	0.64378	280,931	8,005,939
High	XP_016884480.1	PREDICTED: activating signal cointegrator 1 complex subunit 2 isoform X1 [Homo sa	2	92.7	ASCC2	28.409	0.70311	26,817	748,940
High	XP_011536903.1	PREDICTED: calcium-binding and coiled-coil domain-containing protein 1 isoform X:	2	79.7	CALCOCO:	28.325	0.79033	117,359	3,324,196
High	XP_008960788.1	PREDICTED: mitochondrial 2-oxoglutarate/malate carrier protein isoform X1 [Pan pa	4	37.4	SLC25A11	28.297	0.62006	350,107	9,906,833
High	XP_005257516.1	PREDICTED: V-type proton ATPase 116 kDa subunit a isoform X1 [Homo sapiens]	8	97.2	ATP6V0A1	28.285	0.61554	595,563	16,845,285
High	AAH12266.2	ATG12 autophagy related 12 homolog (S. cerevisiae) [Homo sapiens]	1	20.6	ATG12	28.266	0.77182	77,387	2,187,452
High	BAD96553.1	transforming growth factor, beta-induced, 68kDa variant, partial [Homo sapiens]	8	74.6	TGFBI	28.105	0.61942	699,277	19,653,418
High	XP_006715119.1	PREDICTED: MAM domain-containing glycosylphosphatidylinositol anchor protein 1	6	107.3	MDGA1	28.088	0.69562	269,525	7,570,454
High	NP_004854.1	serine palmitoyltransferase 2 [Homo sapiens]	1	62.9	SPTLC2	27.981	0.79161	157,065	4,394,831
High	NP_115970.2	hyccin [Homo sapiens]	6	57.6	FAM126A	27.938	0.60583	372,921	10,418,653
High	AAH01127.1	Ribosomal protein, large, P0 [Homo sapiens]	8	34.3	RPLP0	27.873	0.62138	1,743,973	48,609,139
High	BAB14138.1	unnamed protein product [Homo sapiens]	2	62.2	ELP3	27.853	0.79033	160,649	2,533,633
High	XP_011544439.1	PREDICTED: protein-methionine sulfoxide oxidase MICAL3 isoform X1 [Homo sapier	5	247.5	MICAL3	27.738	0.62138	419,813	11,644,967

High	XP_005245506.1	PREDICTED: SHC-transforming protein 1 isoform X1 [Homo sapiens]	1	64.2	SHC1	27.653	0.78302	106,743	2,951,730
High	AAI08915.1	LRRFIP1 protein [Homo sapiens]	3	89.2	LRRFIP1	27.528	0.77182	73,265	1,959,711
High	NP_001258522.1	protein phosphatase methylesterase 1 isoform b [Homo sapiens]	2	43.8	PPME1	27.384	0.78302	78,118	1,904,414
High	NP_003964.3	60S ribosomal protein L14 [Homo sapiens]	3	23.4	RPL14	27.293	0.72967	227,014	6,195,871
High	NP_002797.3	26S protease regulatory subunit 10B [Homo sapiens]	4	45.8	PSMC6	27.225	0.6489	361,371	8,581,547
High	CAD98028.1	hypothetical protein, partial [Homo sapiens]	4	48.6	FKBP8	27.217	0.68825	291,886	7,944,229
High	NP_001247435.1	40S ribosomal protein S3 isoform 2 [Homo sapiens]	14	28.5	RPS3	27.167	0.63723	2,364,474	64,235,135
High	AAM17922.1	TRK-fused gene/anaplastic large cell lymphoma kinase extra long form [Homo sapie	1	88.6		27.123	0.732	281,073	6,876,464
High	NP_001269526.1	catenin alpha-2 isoform 3 [Homo sapiens]	6	105.2	CTNNA2	27.048	0.78725	71,775	1,941,355
High	BAG59797.1	unnamed protein product [Homo sapiens]	1	84	KIF2A	26.9	0.77182	51,613	1,393,989
High	AKD41818.1	anti-HIV-1 immunoglobulin heavy chain variable region, partial [Homo sapiens]	1	14.3		26.815	0.79033	69,123	1,853,575
High	AAA20993.1	NF45 protein [Homo sapiens]	5	44.7	ILF2	26.712	0.6476	758,469	20,260,258
High	EAX09105.1	hCG32158 [Homo sapiens]	4	139		26.697	0.79096	192,814	5,147,511
High	AAL99921.1	CLL-associated antigen KW-14 [Homo sapiens]	1	78.2	YTHDF2	26.664	0.80285	94,378	2,516,479
High	XP_011514525.1	PREDICTED: hepatocyte growth factor receptor isoform X1 [Homo sapiens]	12	157.6	MET	26.611	0.6489	626,700	16,676,997
High	XP_016871260.1	PREDICTED: inactive ubiquitin carboxyl-terminal hydrolase 54 isoform X2 [Homo sa	2	189.5	USP54	26.464	0.79033	62,413	1,645,877
High	NP_848018.1	coiled-coil domain-containing protein 50 long isoform [Homo sapiens]	2	56.3	CCDC50	26.349	0.80791	108,407	2,856,393
High	NP_005871.1	dnaJ homolog subfamily A member 2 [Homo sapiens]	4	45.7	DNAJA2	26.257	0.755	294,574	6,115,226
High	NP_006055.3	ras-related GTP-binding protein B short isoform [Homo sapiens]	3	40.1	RRAGB	26.173	0.81869	162,113	4,237,031
High	BAG50909.1	unnamed protein product [Homo sapiens]	5	36	TECR	26.114	0.66416	441,714	11,534,952
High	AAH47083.1	Spastic paraplegia 20 (Troyer syndrome) [Homo sapiens]	3	72.8	SPG20; SP	26.028	0.69562	305,306	7,946,492
High	BAA83712.1	ASY [Homo sapiens]	5	40.3	RTN4	25.923	0.67041	741,740	19,228,056
High	AAH14895.2	Aldehyde dehydrogenase 16 family, member A1 [Homo sapiens]	5	85.1	ALDH16A1	25.871	0.80285	196,199	5,075,835
High	EAW53671.1	transferrin receptor (p90, CD71), isoform CRA_b [Homo sapiens]	4	89.7	TFRC	25.611	0.79672	225,216	5,767,993
High	BAC86287.1	unnamed protein product [Homo sapiens]	17	157.9	LRPPRC	25.569	0.68112	1,377,894	35,231,792
High	BAG61388.1	unnamed protein product [Homo sapiens]	10	36.3	SFXN3	25.568	0.68112	1,538,090	39,326,347
High	XP_005269031.1	PREDICTED: ATP-dependent 6-phosphofructokinase, muscle type isoform X1 [Homo	9	96.8	PFKM	25.357	0.75811	288,508	7,315,568
High	XP_005251468.1	PREDICTED: KN motif and ankyrin repeat domain-containing protein 1 isoform X1 [H	5	148.6	KANK1	25.126	0.80313	228,612	5,744,176
High	BAG51120.1	unnamed protein product [Homo sapiens]	9	74.7	SLC25A12	25.103	0.80477	227,035	5,699,259
High	XP_011519908.2	PREDICTED: unconventional myosin-Va isoform X1 [Homo sapiens]	17	221.2	MYO5A	24.933	0.68943	884,343	22,049,682
High	BAG36345.1	unnamed protein product [Homo sapiens]	9	69.6	SYNCRIP	24.878	0.6898	989,360	24,613,622
High	ACF94484.1	epididymis luminal protein 176 [Homo sapiens]	17	25	ACTG1	24.736	0.82572	123,233	2,917,573
High	AAA74235.1	guanine nucleotide regulatory protein [Homo sapiens]	8	44	GNA13	24.663	0.69509	729,556	17,993,347
High	NP_001244127.1	cullin-3 isoform 3 [Homo sapiens]	6	89.7	CUL3	24.642	0.80791	234,740	5,784,555
High	XP_016885310.1	PREDICTED: zinc finger protein 185 isoform X1 [Homo sapiens]	3	81.4	ZNF185	24.577	0.83298	115,987	2,850,580
High	CAA79476.1	caveolin [Homo sapiens]	3	20.5	CAV1	24.561	0.81666	194,689	4,781,816
High	NP_055428.1	FAS-associated factor 2 [Homo sapiens]	1	52.6	FAF2	24.492	0.82572	98,533	2,104,942
High	XP_006713694.1	PREDICTED: leucine-, glutamate- and lysine-rich protein 1 isoform X1 [Homo sapien	1	98.9	LEKR1	24.25	0.82665	84,361	2,045,753
High	NP_009106.1	erlin-2 isoform 1 [Homo sapiens]	6	37.8	ERLIN2	23.965	0.79865	252,064	6,040,818
High	AAA86463.1	Csa-19 [Homo sapiens]	3	24.8	RPL10A	23.868	0.73511	341,451	8,149,622
High	NP_001278910.1	receptor-type tyrosine-protein phosphatase kappa isoform c precursor [Homo sapie	16	164.5	PTPRK	23.793	0.71323	1,090,679	25,950,297
High	AAB60433.1	thymopoietin alpha [Homo sapiens]	3	75.4	TMPO	23.774	0.82572	224,240	5,331,119
High	XP_005254127.1	PREDICTED: tripeptidyl-peptidase 2 isoform X1 [Homo sapiens]	12	144.4	TPP2	23.709	0.71587	1,214,821	28,801,722
High	XP_004645627.1	PREDICTED: peripheral plasma membrane protein CASK isoform X1 [Octodon degus	4	105.1	Cask	23.444	0.86816	160,884	3,771,730
High	NP_149351.1	surfeit locus protein 4 isoform 1 [Homo sapiens]	2	30.4	SURF4	23.375	0.86648	154,448	3,610,236
High	CAB82412.1	hypothetical protein [Homo sapiens]	7	53.9	SLC16A1	23.173	0.72991	1,844,616	42,744,532
High	XP_011539528.1	PREDICTED: D-3-phosphoglycerate dehydrogenase isoform X1 [Homo sapiens]	9	64.7	PHGDH	23.147	0.73032	2,419,648	56,007,814
High	AEE02020.1	NF110b [Homo sapiens]	6	95.7	ILF3	22.904	0.73475	652,080	14,935,100
High	NP_001611.1	neuroblast differentiation-associated protein AHNAK isoform 1 [Homo sapiens]	43	628.7	AHNAK	22.835	0.73676	4,526,576	103,363,663
High	AAA93299.1	p60 [Homo sapiens]	2	47.6	SQSTM1	22.827	0.79033	325,857	7,438,280
High	BAG10224.1	eukaryotic translation initiation factor 4 gamma 1, partial [synthetic construct]	6	176.1		22.692	0.73956	537,292	12,192,259
High	EAW53694.1	guanine nucleotide binding protein (G protein), beta polypeptide 2-like 1, isoform C	8	42.9	GNB2L1; F	22.688	0.73956	1,417,743	32,166,154
High	BAG37193.1	unnamed protein product [Homo sapiens]	2	104	EXOC2	22.478	0.85676	90,162	2,026,616
High	NP_003861.1	ras GTPase-activating-like protein IQGAP1 [Homo sapiens]	67	189.1	IQGAP1	22.396	0.74167	18,401,375	412,111,034
High	CAA45118.1	fibrillin, partial [Homo sapiens]	2	325.5	FBN1	22.286	0.82572	60,376	953,481
High	BAF84156.1	unnamed protein product [Homo sapiens]	1	101.3	MAP4K3	22.243	0.85101	76,977	1,712,201
High	BAG37644.1	unnamed protein product [Homo sapiens]	4	38	CTGF	22.181	0.80285	336,706	7,468,393
High	NP_001278798.1	insulin-like growth factor 2 mRNA-binding protein 2 isoform c [Homo sapiens]	3	66.7	IGF2BP2	21.888	0.80285	348,084	7,619,015
High	XP_005272072.1	PREDICTED: endoplasmic reticulum aminopeptidase 1 isoform X1 [Homo sapiens]	8	108.3	ERAP1	21.837	0.75752	802,782	17,530,000
High	CAH18695.1	hypothetical protein [Homo sapiens]	16	123.3	XPO1	21.825	0.75757	1,143,819	24,963,320
High	AAF99680.1	DNA-binding protein TAXREB107 [Homo sapiens]	8	32.9	RPL6	21.75	0.75875	1,042,813	22,681,043
High	DAA01244.1	TPA_inf: NOD9 [Homo sapiens]	3	107.6	NLRX1	21.72	0.87353	96,309	2,091,455
High	NP_003115.1	sepiapterin reductase [Homo sapiens]	8	28	SPR	21.643	0.7627	1,814,208	39,264,977
High	AAC35008.1	cellular apoptosis susceptibility protein [Homo sapiens]	8	110.3	CSE1L	21.588	0.7642	693,586	14,973,253
High	BAD92713.1	importin 4 variant, partial [Homo sapiens]	3	138.1		21.514	0.85401	67,226	1,433,029
High	XP_011507656.1	PREDICTED: SLIT-ROBO Rho GTPase-activating protein 2 isoform X1 [Homo sapiens]	4	122.2	SRGAP2	21.478	0.76323	479,679	10,302,543
High	NP_002007.1	filaggrin [Homo sapiens]	1	434.9	FLG	21.381	0.80477	14,331	306,409
High	EAX10760.1	hCG1781941, partial [Homo sapiens]	1	32.1		21.333	0.8805	144,733	3,086,714
High	AAA85336.1	transmembrane protein [Homo sapiens]	14	83.6	IMMT	21.206	0.77112	1,831,583	38,840,586
High	BAG37313.1	unnamed protein product [Homo sapiens]	34	112.8	ATP1A1	21.184	0.77136	5,178,885	109,708,356
High	XP_016877212.1	PREDICTED: alpha-actinin-1 isoform X4 [Homo sapiens]	56	121.9	ACTN1	21.046	0.77182	7,712,873	162,323,948
High	XP_016793385.1	PREDICTED: proto-oncogene tyrosine-protein kinase Src isoform X2 [Pan troglodyte	14	60.6	SRC	20.829	0.77943	1,886,995	39,303,984
High	5A2Q_G	Chain G, Structure Of The Hcv Ires Bound To The Human Ribosome	1	28.7		20.615	0.8805	256,926	5,296,441
High	EAW59030.1	inhibitor of kappa light polypeptide gene enhancer in B-cells, kinase complex-associ	3	162.9	IKBKAP; EI	20.581	0.88535	245,880	5,060,488
High	AAH95485.1	Membrane protein, palmitoylated 5 (MAGUK p55 subfamily member 5) [Homo sapi	3	77.3	MPP5	20.56	0.88077	238,101	4,895,446
High	AAG53603.1	RANBP21 [Homo sapiens]	1	136.3	XPO5	20.477	0.85051	40,656	820,731
High	BAG37316.1	unnamed protein product [Homo sapiens]	16	97.1	KPNB1	20.475	0.78964	2,525,033	51,700,829
High	NP_036524.1	peflin [Homo sapiens]	3	30.4	PEF1	20.426	0.8942	105,204	2,184,217
High	BAD96840.1	serine racemase variant, partial [Homo sapiens]	3	36.6	SRR	20.406	0.91154	202,564	4,133,566
High	AAM33633.1	URB [Homo sapiens]	4	108.1	CCDC80	20.405	0.9059	139,861	2,853,820
High	1HZJ_A	Chain A, Human Udp-Galactose 4-Epimerase: Accommodation Of Udp-N- Acetylgluc	2	38.3		20.356	0.89051	230,970	4,701,645
High	NP_056281.1	tyrosine-protein phosphatase non-receptor type 23 isoform 1 [Homo sapiens]	5	178.9	PTPN23	20.319	0.79876	445,004	9,042,065
High	XP_016874792.1	PREDICTED: prolow-density lipoprotein receptor-related protein 1 isoform X1 [Hom	27	506	LRP1	20.279	0.79033	2,487,559	50,444,435
High	XP_005254037.1	PREDICTED: ankyrin repeat domain-containing protein 13A isoform X1 [Homo sapie	1	67.7	ANKRD13A	20.218	0.8946	113,576	2,296,313
High	NP_004915.2	alpha-actinin-4 isoform 1 [Homo sapiens]	62	104.8	ACTN4	20.213	0.79161	40,918,690	827,098,499
High	AAQ02560.1	casein kinase 1, alpha 1, partial [synthetic construct]	1	39		20.121	0.86816	52,143	1,049,161
High	NP_001129224.1	protein ecdysoneless homolog isoform 2 [Homo sapiens]	3	76.5	ECD	20.098	0.91583	223,181	4,502,162
High	AAM09527.1	macrothioredoxin [Homo sapiens]	27	86.1	DNAJC10	20.078	0.7949	868,651	17,440,370
High	AAH13590.1	Eukaryotic translation initiation factor 2B, subunit 5 epsilon, 82kDa [Homo sapiens]	1	80.3	EIF2B5	20.056	0.91181	147,890	2,966,069
High	BAF84471.1	unnamed protein product [Homo sapiens]	2	88.9	FCHO2	19.98	0.88069	69,487	1,388,330
High	AAB47425.1	bacterial MutS homolog [Homo sapiens]	5	152.8	MSH6	19.659	0.90216	235,385	4,627,422
High	XP_011516714.1	PREDICTED: constitutive coactivator of PPAR-gamma-like protein 1 isoform X1 [Hon	5	125.2	FAM120A	19.604	0.90216	288,655	5,658,829
High	NP_000518.1	low-density lipoprotein receptor isoform 1 precursor [Homo sapiens]	8	95.3	LDLR	19.396	0.80791	1,186,438	23,011,569
High	NP_000453.2	ubiquitin-protein ligase E3A isoform 2 [Homo sapiens]	2	100.6	UBE3A	19.353	0.86492	322,147	6,234,453
High	NP_065789.1	kinase D-interacting substrate of 220 kDa [Homo sapiens]	2	196.4	KIDINS220	19.009	0.90216	64,294	1,222,150

High	NP_001041675.1	chloride channel CLIC-like protein 1 isoform 1 precursor [Homo sapiens]	2	62	CLCC1	18.962	0.87084	385,854	7,316,671
High	AHN96194.1	MHC class I antigen, partial [Homo sapiens]	10	31.8	HLA-A	18.595	0.91606	280,471	5,114,503
High	BAD93080.1	proteasome 26S non-ATPase subunit 2 variant, partial [Homo sapiens]	16	100.5	PSMD2	18.575	0.82876	2,119,518	39,369,463
High	XP_016878013.1	PREDICTED: tight junction protein ZO-1 isoform X6 [Homo sapiens]	26	201.8	TJP1	18.456	0.93392	153,735	2,837,349
High	AAA35750.1	DNA-binding protein B, partial [Homo sapiens]	2	40	YBX1	18.387	0.93392	176,841	3,251,547
High	AAD29048.1	histone deacetylase 6 [Homo sapiens]	20	131.3	HDAC6	18.357	0.83298	8,189,483	150,337,669
High	XP_011543091.1	PREDICTED: serine/threonine-protein kinase MARK2 isoform X1 [Homo sapiens]	1	89.8	MARK2	18.305	0.93392	149,817	2,109,443
High	AAC24312.1	Unknown gene product [Homo sapiens]	3	40	CIAPIN1	18.248	0.93013	310,334	5,662,866
High	BAD97283.1	cytoskeleton-associated protein 4 variant, partial [Homo sapiens]	10	66	CKAP4	18.229	0.83612	1,758,000	32,046,442
High	XP_011538066.1	PREDICTED: tumor necrosis factor receptor superfamily member 6 isoform X1 [Hom	1	43.6	FAS	18.216	0.90216	49,283	897,608
High	XP_016882520.1	PREDICTED: proline-rich protein 12 isoform X1 [Homo sapiens]	1	146.8	PRR12	18.116	0.93392	124,155	2,249,189
High	NP_775823.1	deleted in autism protein 1 isoform a precursor [Homo sapiens]	2	49.5	C3orf58	18.006	0.93392	207,739	3,740,528
High	NP_694578.1	BTB/POZ domain-containing protein KCTD7 isoform 1 [Homo sapiens]	4	33.1	KCTD7	17.98	0.84251	596,899	10,732,327
High	XP_011529261.1	PREDICTED: moesin isoform X1 [Homo sapiens]	18	71.3	MSN	17.902	0.84845	839,262	15,024,351
High	NP_006406.1	serine palmitoyltransferase 1 isoform a [Homo sapiens]	6	52.7	SPTLC1	17.666	0.85443	1,290,708	22,801,758
High	AAD30280.1	endocytic receptor Endo180 [Homo sapiens]	1	166.6	MRC2	17.543	0.9285	65,845	1,155,123
High	NP_001291217.1	40S ribosomal protein SA isoform 2 [Homo sapiens]	8	33.3	RPSA	17.54	0.8574	3,816,006	66,932,801
High	XP_005271136.1	PREDICTED: receptor-type tyrosine-protein phosphatase F isoform X1 [Homo sapier	3	214.3	PTPRF	17.385	0.93392	125,595	2,160,941
High	NP_002699.1	serine/threonine-protein phosphatase PP1-alpha catalytic subunit isoform 1 [Homo	17	37.5	PPP1CA	17.372	0.86278	1,107,583	19,240,695
High	AAG34678.1	vacuolar protein sorting protein 16 [Homo sapiens]	2	94.6	VPS16	17.358	0.87583	463,132	8,039,137
High	BAD96317.1	ubiquitin domain containing 1 variant, partial [Homo sapiens]	2	25.8	UBTD1	17.342	0.93392	198,340	3,376,449
High	BAA75062.1	apg-2 [Homo sapiens]	1	94.2	HSPA4	17.279	0.93392	214,360	3,714,492
High	AAK69110.1	PWP1-interacting protein 4 [Homo sapiens]	1	40.5	DNAJB11	17.246	0.87463	523,095	9,021,095
High	XP_005259654.1	PREDICTED: polypyrimidine tract-binding protein 1 isoform X1 [Homo sapiens]	7	59.8	PTBP1	17.231	0.86728	1,468,802	25,308,571
High	NP_004388.2	probable ATP-dependent RNA helicase DDX6 [Homo sapiens]	6	54.4	DDX6	17.173	0.86816	767,532	13,180,454
High	NP_055726.3	AP2-associated protein kinase 1 [Homo sapiens]	2	103.8	AAK1	17.124	0.93392	97,474	1,669,186
High	AAA50598.1	homolog of Drosophila discs large protein, isoform 2 [Homo sapiens]	7	103.2	DLG1	17.075	0.87084	1,593,223	27,203,837
High	AAF66130.1	transmembrane protein BRI [Homo sapiens]	1	31.8	ITM2B	17.033	0.91181	45,106	760,868
High	NP_002601.1	[Pyruvate dehydrogenase (acetyl-transferring)] kinase isozyme 1, mitochondrial isof	2	49.2	PDK1	16.937	0.93392	389,849	4,506,132
High	AAC26080.1	homolog of the Aspergillus nidulans sudD gene product [Homo sapiens]	2	59.1	RIOK3	16.805	0.93392	406,901	5,835,580
High	XP_005264612.1	PREDICTED: CAD protein isoform X1 [Homo sapiens]	16	244.6	CAD	16.703	0.8805	2,087,911	34,874,146
High	XP_006719595.1	PREDICTED: paxillin isoform X3 [Homo sapiens]	3	121.1	PXN	16.599	0.93392	239,690	3,978,641
High	EAX05831.1	BMP2 inducible kinase, isoform CRA_c [Homo sapiens]	5	129.7	BMP2K	16.58	0.88077	1,505,733	24,964,752
High	EAW67508.1	Rho guanine nucleotide exchange factor (GEF) 12, isoform CRA_a [Homo sapiens]	1	174.5	ARHGEF1	16.433	0.93392	170,252	2,797,810
High	NP_005558.1	galectin-3-binding protein precursor [Homo sapiens]	14	65.3	LGALS3BP	16.401	0.88535	4,839,890	79,381,449
High		T47177 hypothetical protein DKFZp762H157.1 - human (fragment)	36	73.9		16.385	0.88578	13,440,807	220,229,533
High	AAH32229.1	AXL receptor tyrosine kinase [Homo sapiens]	5	98.3	AXL	16.346	0.88732	769,732	12,582,219
High	NP_005879.1	phosphate carrier protein, mitochondrial isoform a precursor [Homo sapiens]	15	40.1	SLC25A3	16.329	0.88785	11,840,522	193,343,468
High	NP_001012680.1	4F2 cell-surface antigen heavy chain isoform b [Homo sapiens]	2	68.1	SLC3A2	16.246	0.93392	302,225	4,910,032
High	AAA18904.1	microtubule-associated protein 1B [Homo sapiens]	9	270.5	MAP1B	16.241	0.89051	1,104,186	17,933,418
High	BAA91471.1	unnamed protein product [Homo sapiens]	1	28.7	NUBP2	16.149	0.93392	185,035	2,988,213
High	BAG37888.1	unnamed protein product [Homo sapiens]	19	29	TPM3	16.114	0.8946	2,051,684	33,059,886
High	AAA59183.1	integrin beta-5 subunit precursor [Homo sapiens]	6	88	ITGB5	16.009	0.89818	803,100	12,856,678
High	BAA05519.1	membrane-type matrix metalloproteinase [Homo sapiens]	3	65.8	MMP14	15.993	0.93392	232,817	3,723,552
High	AAA20967.1	syntaxin [Homo sapiens]	1	34.2	STX4	15.955	0.93392	120,763	1,926,821
High	EAW73515.1	histone deacetylase 10, isoform CRA_e [Homo sapiens]	12	71.8	HDAC10	15.944	0.90071	5,161,844	82,302,119
High	AAH16812.1	ATP synthase, H+ transporting, mitochondrial F1 complex, gamma polypeptide 1 [H	4	32.9	ATP5C1	15.857	0.90216	1,314,111	20,838,445
High	BAG65446.1	unnamed protein product [Homo sapiens]	2	68	GPNMB	15.848	0.90216	3,710,859	58,809,304
High	AAD05029.1	unknown [Homo sapiens]	5	78.9	NCDN	15.772	0.93392	454,835	6,836,227
High	AAB87693.1	importin-alpha homolog [Homo sapiens]	1	57.7	KPNA3	15.7	0.93392	67,422	1,058,499
High	NP_079106.3	lysophosphatidylcholine acyltransferase 1 [Homo sapiens]	2	59.1	LPCAT1	15.683	0.93392	203,048	3,184,369
High	AAC60637.1	putative cytoskeletal protein [Homo sapiens]	1	65.9	CCDC6	15.658	0.93392	32,966	532,109
High	AAB24841.1	Tat binding protein 7, TBP-7=transcriptional activator [human, Peptide, 458 aa]	3	51.5		15.595	0.93392	260,178	4,057,551
High	XP_016872004.1	PREDICTED: MMS19 nucleotide excision repair protein homolog isoform X1 [Homo	8	117.5	MMS19	15.562	0.91181	1,028,785	16,010,118
High	EAW61129.1	drebrin-like, isoform CRA_a [Homo sapiens]	8	55.3	DBNL	15.535	0.91181	2,172,816	33,755,188
High	AAS48345.1	histone deacetylase 10 [Homo sapiens]	8	43	HDAC10	15.46	0.93392	84,057	1,299,523
High	AAX82302.1	actin-like protein, partial [Homo sapiens]	5	11.5	ACTG1	15.456	0.91389	3,931,710	60,767,024
High	AAL35904.1	protein phosphatase type 2A catalytic subunit [Homo sapiens]	5	35.6	PPP2CB	15.359	0.91583	770,258	11,830,412
High	NP_006078.2	tubulin beta-4A chain isoform 3 [Homo sapiens]	29	49.6	TUBB4A	15.321	0.9251	616,569	9,446,249
High	XP_011537894.1	PREDICTED: probable E3 ubiquitin-protein ligase HERC4 isoform X1 [Homo sapiens]	2	121.1	HERC4	15.306	0.91181	665,224	10,181,734
High	BAF84486.1	unnamed protein product [Homo sapiens]	3	69.9	PPP2R5D	15.263	0.9353	227,204	3,431,202
High	NP_033473.1	tubulin alpha-4A chain isoform 1 [Mus musculus]	26	49.9	Tuba4a	15.214	0.92181	8,763,312	133,325,085
High	EAX05872.1	heterogeneous nuclear ribonucleoprotein D (AU-rich element RNA binding protein	3	40.8	HNRNPD	15.083	0.93392	502,103	7,573,093
High	EAX08336.1	poly (ADP-ribose) polymerase family, member 4 [Homo sapiens]	40	192.4	PARP4	15.081	0.92541	14,042,307	211,768,847
High	EAW81764.1	dynein, cytoplasmic 1, heavy chain 1, isoform CRA_f [Homo sapiens]	1	532.5	DYNC1H1	15.066	0.93392	13,013	243,573
High	XP_014199518.1	PREDICTED: signal transducer and activator of transcription 1-alpha/beta isoform X:	9	87.6	STAT1	15.065	0.92541	1,204,525	18,145,933
High	XP_016863821.1	PREDICTED: UPF0609 protein C4orf27 isoform X1 [Homo sapiens]	1	41.7	C4orf27; I	14.94	0.93392	66,633	995,527
High	XP_005264262.1	PREDICTED: EGF-containing fibulin-like extracellular matrix protein 1 isoform X1 [Ho	5	59.3	EFEMP1	14.841	0.93392	359,882	5,341,041
High	AAL56011.1	very-long-chain acyl-CoA dehydrogenase VLCAD [Homo sapiens]	5	68.7	ACAD9	14.775	0.93392	491,779	7,266,174
High	NP_149124.3	2',3'-cyclic-nucleotide 3'-phosphodiesterase isoform 1 [Homo sapiens]	16	47.5	CNP	14.737	0.93392	3,424,248	50,464,494
High	ACX50452.1	MHC class I antigen, partial [Homo sapiens]	3	21		14.736	0.93392	206,165	3,037,983
High	BAD96399.1	proteasome 26S non-ATPase subunit 12 isoform 1 variant, partial [Homo sapiens]	2	52.9	PSMD12	14.735	0.93933	285,428	4,205,655
High	AAC63263.1	retinal short-chain dehydrogenase/reductase retSDR1 [Homo sapiens]	1	33.5	DHRS3	14.734	0.93472	193,175	2,846,295
High	NP_065812.1	regulatory-associated protein of mTOR isoform 1 [Homo sapiens]	1	148.9	RPTOR	14.717	0.93392	29,537	439,964
High	XP_016879246.1	PREDICTED: protein spinster homolog 1 isoform X1 [Homo sapiens]	1	62.5	SPNS1	14.696	0.93392	155,510	2,285,330
High	AAH28399.1	ACSF3 protein [Homo sapiens]	10	64.1	ACSF3	14.585	0.93392	1,726,920	25,187,078
High	BAE45763.1	putative protein product of Nbla10058 [Homo sapiens]	2	48.6	PSMC2	14.57	0.93986	200,412	2,920,026
High	BAD92053.1	calpastatin isoform a variant, partial [Homo sapiens]	3	84.2	CAST	14.552	0.93392	375,551	5,465,126
High	EAW70720.1	tubulin, alpha 4, isoform CRA_a [Homo sapiens]	1	6.5	TUBA4B	14.515	0.95193	232,896	3,380,402
High	BAD96601.1	steroid dehydrogenase homolog, partial [Homo sapiens]	3	34.3	HSD17B12	14.419	0.93809	311,032	4,484,785
High	BAD92710.1	centaurin delta 2 isoform a variant, partial [Homo sapiens]	1	164.4		14.378	0.93392	45,050	647,743
High	NP_004199.1	apoptosis-inducing factor 1, mitochondrial isoform AIF precursor [Homo sapiens]	14	66.9	AIFM1	14.363	0.93392	3,388,943	48,673,807
High	AAH11719.1	Transmembrane protein 43 [Homo sapiens]	1	44.8	TMEM43	14.354	0.94634	207,127	2,973,058
High	XP_003808090.1	PREDICTED: target of rapamycin complex 2 subunit MAPKAP1 isoform X1 [Pan panis	1	64.4	MAPKAP1	14.344	0.93392	38,424	557,556
High	XP_011532899.1	PREDICTED: endoplasmic reticulum-Golgi intermediate compartment protein 1 isofo	5	35.1	ERGIC1	14.298	0.93392	2,919,619	41,744,357
High	XP_016880861.1	PREDICTED: puromycin-sensitive aminopeptidase isoform X1 [Homo sapiens]	3	104	NPEPPS	14.268	0.96072	258,125	3,682,893
High	XP_003831370.1	PREDICTED: 60S ribosomal protein L7 isoform X1 [Pan paniscus]	4	30.4	RPL7	14.226	0.93392	1,735,764	24,693,520
High	XP_011537116.1	PREDICTED: early endosome antigen 1 isoform X1 [Homo sapiens]	2	167.2	EEA1	14.206	0.9508	205,214	2,915,290
High	NP_036359.3	putative GTP-binding protein 6 [Homo sapiens]	2	56.9	GTPBP6	14.205	0.9602	246,713	3,504,661
High	BAG35362.1	unnamed protein product [Homo sapiens]	1	59.2	PPM1G	14.174	0.93392	87,064	1,234,030
High	XP_005266116.1	PREDICTED: mitochondrial-processing peptidase subunit alpha isoform X1 [Homo sa	4	61.7	PMPCA	14.153	0.93944	349,122	4,941,016
High	AAF13156.1	double stranded RNA activated protein kinase [Homo sapiens]	1	62	EIF2AK2	14.07	0.93392	75,917	1,068,414
High	AAK61251.1	Weakly similar to ORF YNL240c [S.cerevisiae] [Homo sapiens]	2	58.3	NARFL	14.045	0.94259	161,580	2,269,329
High	AAG50180.1	tripartite motif protein TRIM19 alpha [Homo sapiens]	11	97.5	PML	14.022	0.93392	1,695,680	23,776,194
High	CAG28544.1	ATP1B1, partial [Homo sapiens]	5	35	ATP1B1	14.014	0.93392	1,237,621	17,344,281

High	XP_006715894.1	PREDICTED: calcium-binding mitochondrial carrier protein Aralar2 isoform X1 [Hom	23	75.4	SLC25A13	14	0.93392	3,902,982	54,640,866
High	XP_006713191.1	PREDICTED: inosine-5'-monophosphate dehydrogenase 2 isoform X1 [Homo sapien	18	66.1	IMPDH2	13.938	0.93392	5,813,902	81,032,679
High	NP_001091868.1	coatomer subunit alpha isoform 1 [Homo sapiens]	11	139.2	COPA	13.91	0.93392	2,051,123	28,532,112
High	AAN87335.1	class IVb beta tubulin [Homo sapiens]	30	49.7	TUBB4B	13.804	0.93392	11,057,764	152,646,874
High	EAW61235.1	proteasome (prosome, macropain) 26S subunit, non-ATPase, 13, isoform CRA_b [Ho	2	42.9	PSMD13	13.711	0.96562	285,705	3,917,222
High	AAV38817.1	tyrosine 3-monooxygenase/tryptophan 5-monooxygenase activation protein, theta	4	27.7	YWHAQ	13.673	0.95601	207,346	2,835,120
High	AAH20946.1	Tubulin, beta [Homo sapiens]	30	49.6	TUBB	13.596	0.93392	38,843,827	528,112,950
High	NP_055577.1	delta(24)-sterol reductase precursor [Homo sapiens]	1	60.1	DHCR24	13.487	0.96625	300,982	4,059,313
High	BAA06126.1	KIAA0115 [Homo sapiens]	7	50.7	DDOST	13.474	0.93392	3,506,598	47,247,441
High	AAH68456.2	Tyrosine 3-monooxygenase/tryptophan 5-monooxygenase activation protein, zeta p	8	27.7	YWHAZ	13.443	0.93392	1,147,053	15,420,259
High	BAF84660.1	unnamed protein product [Homo sapiens]	9	46.1	EIF4A1	13.398	0.93392	2,268,348	30,390,748
High	AAH11762.1	Cytoplasmic FMR1 interacting protein 2 [Homo sapiens]	18	145.6	CYFIP2	13.386	0.96625	267,245	3,577,414
High	AAH68012.1	ATXN2L protein, partial [Homo sapiens]	3	115.5	ATXN2L	13.312	0.93635	479,288	6,380,355
High	AAC51317.1	karyopherin beta 3 [Homo sapiens]	21	123.5	IPO5	13.278	0.93392	3,734,660	49,588,125
High	BAD93013.1	prosaposin variant, partial [Homo sapiens]	1	58.7	PSAP	13.234	0.94349	80,151	1,082,301
High	XP_005265952.1	PREDICTED: heterogeneous nuclear ribonucleoprotein H isoform X1 [Homo sapiens	6	51.2	HNRNPH1	13.19	0.93392	1,144,237	15,092,837
High	NP_001317652.1	sorting nexin-27 isoform 1 [Homo sapiens]	2	61.2	SNX27	13.155	0.96484	152,321	2,003,840
High	CAA07022.1	beta-tubulin cofactor D [Homo sapiens]	8	138.6	TBCD	13.135	0.93392	1,268,089	16,656,389
High	BAH13586.1	unnamed protein product [Homo sapiens]	23	54.5	DNPEP	13.1	0.93392	33,728,118	441,823,023
High	AAG22575.1	PELOTA [Homo sapiens]	7	43.5	PELO	13.038	0.93392	1,170,194	15,256,583
High	NP_001129498.1	protein SEC13 homolog isoform 3 [Homo sapiens]	1	40.7	SEC13	12.992	0.96625	209,268	2,038,642
High	NP_733765.1	sarcoplasmic/endoplasmic reticulum calcium ATPase 2 isoform b [Homo sapiens]	24	114.7	ATP2A2	12.985	0.93392	6,230,933	80,907,736
High	BAD93149.1	thrombospondin 1 precursor variant, partial [Homo sapiens]	30	134.8	THBS1	12.975	0.93392	22,385,186	290,440,582
High	NP_001250.1	cyclin-dependent kinase 6 [Homo sapiens]	2	36.9	CDK6	12.963	0.95193	76,538	979,030
High	ABQ50603.1	non-functional aryl hydrocarbon receptor interacting protein, partial [Homo sapiens	5	40.7	AIP	12.9	0.93392	1,916,980	24,729,446
High	BAF83546.1	unnamed protein product [Homo sapiens]	10	101.1	MARS	12.894	0.93392	2,754,708	35,519,150
High	AAA61932.1	putative nucleotide-binding protein [Homo sapiens]	3	34.6	NUBP1	12.874	0.93392	941,880	12,125,816
High	AAC16903.1	TGF beta receptor associated protein-1 [Homo sapiens]	1	97.1	TGFBRAP1	12.797	0.93472	34,746	444,541
High	CAD97622.1	hypothetical protein, partial [Homo sapiens]	1	75.2	ACOX1	12.783	0.96288	449,587	5,747,128
High	XP_006904422.1	PREDICTED: exportin-7 isoform X1 [Pteropus alecto]	1	124.9	XPO7	12.783	0.9568	77,195	985,854
High	NP_060422.4	pentatricopeptide repeat domain-containing protein 3, mitochondrial precursor [Ho	1	78.5	PTCD3	12.772	0.96562	127,345	1,626,451
High	NP_078939.3	probable aminopeptidase NPEPL1 isoform 1 [Homo sapiens]	3	55.8	NPEPL1	12.768	0.95024	524,943	6,702,415
High	XP_011523430.1	PREDICTED: E3 ubiquitin-protein ligase SMURF2 isoform X1 [Homo sapiens]	2	86.4	SMURF2	12.693	0.9704	331,564	4,208,438
High	BAG37619.1	unnamed protein product [Homo sapiens]	9	134.4	LARS	12.69	0.93392	1,085,673	13,777,345
High	BAF83740.1	unnamed protein product [Homo sapiens]	4	56.2	PSMD5	12.681	0.95024	487,748	6,185,112
High	NP_849193.1	dolichyl-diphosphooligosaccharide--protein glycosyltransferase subunit STT3B [Hom	1	93.6	STT3B	12.606	0.96625	179,592	2,307,767
High	BAG58089.1	unnamed protein product [Homo sapiens]	1	42.4		12.502	0.96625	125,874	1,573,730
High	BAA25517.2	KIAA0591 protein, partial [Homo sapiens]	1	208.3	KIF1B	12.5	0.9704	198,919	2,486,538
High	NP_079477.2	pleckstrin homology domain-containing family O member 2 isoform 1 [Homo sapien	2	53.3	PLEKHO2	12.401	0.96625	453,894	5,613,980
High	NP_055144.3	enhancer of mRNA-decapping protein 4 [Homo sapiens]	1	151.6	EDC4	12.393	0.96625	99,656	1,235,012
High	XP_001138227.3	PREDICTED: galactokinase [Pan troglodytes]	10	45.3	GALK1	12.372	0.93392	3,672,649	45,437,739
High	CAA50592.1	vacuolar proton ATPase [Homo sapiens]	1	26.2	ATP6V1E1	12.372	0.93944	42,830	446,192
High	XP_011515603.1	PREDICTED: gasdermin-D isoform X1 [Homo sapiens]	1	74.8	GSDMD	12.371	0.96625	134,594	1,668,978
High	NP_001034702.1	isoamyl acetate-hydrolyzing esterase 1 homolog isoform a precursor [Homo sapiens	2	27.6	IAH1	12.357	0.96072	491,354	6,071,694
High	AAC50934.1	acetolactate synthase homolog [Homo sapiens]	3	67.9	ILVBL	12.347	0.97411	201,571	2,488,732
High	BAD93131.1	integrin alpha-V precursor variant, partial [Homo sapiens]	5	121.6	ITGAV	12.328	0.93449	917,500	11,310,765
High	CAA88742.1	OA1 [Homo sapiens]	4	46	GPR143	12.326	0.93635	703,759	8,674,887
High	XP_011527207.1	PREDICTED: acetyl-coenzyme A synthetase, cytoplasmic isoform X1 [Homo sapiens]	1	82.7	ACSS2	12.257	0.96127	67,481	827,147
High	XP_011538278.1	PREDICTED: transmembrane 9 superfamily member 3 isoform X1 [Homo sapiens]	2	70.2	TM9SF3	12.214	0.98284	297,127	3,629,234
High	BAH13250.1	unnamed protein product [Homo sapiens]	14	62.7	CALD1	12.159	0.93635	3,874,357	47,108,344
High	BAG59593.1	unnamed protein product [Homo sapiens]	3	48.1		12.159	0.96356	579,875	7,050,814
High	NP_001001935.1	ATP synthase subunit alpha, mitochondrial isoform c [Homo sapiens]	23	54.5	ATP5A1	12.154	0.96892	459,727	5,587,585
High	NP_065174.1	coronin-1B [Homo sapiens]	6	54.2	CORO1B	12.152	0.93635	1,102,783	13,400,640
High	NP_001301006.1	vitamin K-dependent protein S isoform 1 precursor [Homo sapiens]	3	78.8	PROS1	12.077	0.93944	997,417	12,045,346
High	AAH28337.1	Glucosidase I [Homo sapiens]	1	91.9	MOGS	12.004	0.96684	106,929	1,280,863
High	NP_001612.1	aryl hydrocarbon receptor [Homo sapiens]	2	96.1	AHR	11.975	0.97673	195,637	2,342,671
High	BAC11130.1	unnamed protein product [Homo sapiens]	1	49.3	GRWD1	11.963	0.97829	173,187	2,051,039
High	XP_006718553.1	PREDICTED: echinoderm microtubule-associated protein-like 3 isoform X3 [Homo sa	1	100.8	EML3	11.931	0.9704	134,573	1,605,527
High	CAA07619.2	lysine-ketoglutarate reductase /saccharopine dehydrogenase [Homo sapiens]	3	102.1	AASS	11.896	0.9785	160,438	1,881,933
High	BAG57168.1	unnamed protein product [Homo sapiens]	2	89.1	LRCH3	11.883	0.98363	369,613	4,392,279
High	NP_001609.2	poly [ADP-ribose] polymerase 1 [Homo sapiens]	34	113	PARP1	11.878	0.94726	12,123,940	144,007,818
High	BAD92231.1	tripartite motif-containing 25 variant, partial [Homo sapiens]	7	72.2	TRIM25	11.864	0.94803	1,892,025	22,446,853
High	NP_004095.4	fatty acid synthase [Homo sapiens]	105	273.3	FASN	11.814	0.95024	20,239,742	239,105,762
High	AAF86954.1	GK001 [Homo sapiens]	1	55.8	CCDC47	11.777	0.96932	98,494	1,159,939
High	XP_005274035.1	PREDICTED: phosphatidylinositol 3,4,5-trisphosphate 5-phosphatase 2 isoform X1 [I	11	140.9	INPPL1	11.763	0.95193	1,994,094	23,455,707
High	CAD97649.1	hypothetical protein, partial [Homo sapiens]	5	91.1	ITGB1	11.761	0.96625	517,525	6,086,802
High	NP_114152.3	arf-GAP with GTPase, ANK repeat and PH domain-containing protein 3 isoform a [Ho	1	97.9	AGAP3	11.75	0.96653	76,122	883,316
High	EAW93310.1	glutamyl-prolyl-tRNA synthetase, isoform CRA_b [Homo sapiens]	29	171.5	EPRS	11.734	0.95266	5,750,658	67,477,893
High	NP_001171725.1	extended synaptotagmin-1 isoform 1 [Homo sapiens]	8	123.9	ESYT1	11.734	0.95266	2,253,013	26,436,896
High	CAA55016.1	hnRNP-E1 [Homo sapiens]	13	37.5	PCBP1	11.724	0.95286	13,923,865	163,246,223
High	EAW68921.1	heterogeneous nuclear ribonucleoprotein M, isoform CRA_c [Homo sapiens]	15	77.6	HNRNPM	11.712	0.95324	2,610,224	30,572,066
High	NP_001253938.1	ADP/ATP translocase 2 [Macaca mulatta]	17	32.9	SLC25A5	11.641	0.9568	5,891,256	68,578,092
High	XP_005267329.1	PREDICTED: exocyst complex component 5 isoform X1 [Homo sapiens]	2	85.4	EXOC5	11.607	0.96867	508,918	5,906,968
High	XP_005254765.1	PREDICTED: talin-2 isoform X1 [Homo sapiens]	1	273	TLN2	11.588	0.99335	346,526	4,015,651
High	BAG70196.1	ATP-dependent RNA helicase DDX5, partial [Homo sapiens]	8	69.1	DDX5	11.562	0.98284	478,066	5,527,263
High	XP_011541007.1	PREDICTED: pleckstrin homology-like domain family B member 1 isoform X4 [Homo	6	171.8	PHLDB1	11.502	0.98522	394,016	4,532,075
High	NP_001026976.1	fatty aldehyde dehydrogenase isoform 1 [Homo sapiens]	5	57.6	ALDH3A2	11.461	0.96444	795,906	9,122,014
High	AAQ13609.1	MSTP086 [Homo sapiens]	2	51.2	HM13	11.46	0.99484	223,410	2,547,236
High	NP_037386.1	serine/threonine-protein kinase TBK1 [Homo sapiens]	5	83.6	TBK1	11.453	0.96444	804,489	9,213,968
High	NP_001078927.1	catenin delta-1 isoform 1ABC [Homo sapiens]	13	108.1	CTNND1	11.431	0.96146	3,136,434	35,853,090
High	EAW58883.1	TBC1 domain family, member 2, isoform CRA_b [Homo sapiens]	6	106.9	TBC1D2	11.414	0.96562	729,429	8,325,840
High	AAH34051.1	Serine/threonine kinase 11 interacting protein [Homo sapiens]	1	121.3	STK11IP	11.352	0.98878	394,427	4,477,526
High	NP_079483.3	FAD synthase isoform 1 [Homo sapiens]	8	65.2	FLAD1	11.278	0.96562	990,498	11,170,531
High	XP_005256095.1	PREDICTED: protein VAC14 homolog isoform X1 [Homo sapiens]	2	88.6	VAC14	11.271	0.99056	264,567	2,981,838
High	EAW84621.1	glycosyltransferase 25 domain containing 1, isoform CRA_a [Homo sapiens]	6	74	COLGALT1	11.266	0.96444	906,547	10,213,174
High	NP_003306.3	dnaJ homolog subfamily C member 7 isoform 1 [Homo sapiens]	1	56.4	DNAJC7	11.241	0.9807	127,789	1,436,534
High	BAD97315.1	ribophorin I variant, partial [Homo sapiens]	11	68.5	RPN1	11.232	0.96625	2,814,369	31,611,102
High	NP_054706.1	vinculin isoform meta-VCL [Homo sapiens]	1	123.7	VCL	11.182	0.96625	18,753	210,979
High	XP_016871767.1	PREDICTED: nuclear factor NF-kappa-B p100 subunit isoform X1 [Homo sapiens]	3	115.8	NFKB2	11.137	0.99613	359,022	3,998,329
High	AAI43937.1	FLRT2 protein [Homo sapiens]	4	74	FLRT2	11.116	0.97829	571,481	6,352,775
High	EAW60840.1	Coenzyme A synthase, isoform CRA_c [Homo sapiens]	5	65.4	COASY	11.076	0.96625	1,560,656	17,285,988
High	EAW52998.1	lamin A/C, isoform CRA_c [Homo sapiens]	16	87.3	LMNA	11.033	0.96625	5,515,892	60,856,060
High	AAH51192.1	Splicing factor proline/glutamine-rich (polypyrimidine tract binding protein associat	10	76.1	SFPQ	11.032	0.96625	2,644,804	29,177,360
High	NP_004198.1	monocarboxylate transporter 4 [Homo sapiens]	9	49.4	SLC16A3	10.999	0.96625	27,034,785	297,352,933
High	BAD92224.1	solute carrier family 2 (facilitated glucose transporter), member 1 variant, partial [H	7	57	SLC2A1	10.999	0.96625	12,102,084	133,112,964

High	BAG10083.1	ankyrin repeat domain-containing protein 25, partial [synthetic construct]	18	91.1		10.981	0.96625	4,448,572	48,849,876
High	XP_005260781.1	PREDICTED: 1-phosphatidylinositol 4,5-bisphosphate phosphodiesterase beta-4 isof	6	137.4	PLCB4	10.905	0.96653	1,103,009	12,027,921
High	EAW60826.1	signal transducer and activator of transcription 3 (acute-phase response factor), iso	13	88.7	STAT3	10.879	0.96684	3,033,458	32,999,549
High	NP_001748.1	carbonyl reductase [NADPH] 1 isoform 1 [Homo sapiens]	2	30.4	CBR1; SET	10.879	0.98878	304,019	3,269,953
High	NP_036450.1	LETM1 and EF-hand domain-containing protein 1, mitochondrial precursor [Homo s	1	83.3	LETM1	10.872	0.99094	144,763	1,573,863
High	BAD92749.1	epithelial protein lost in neoplasm beta variant, partial [Homo sapiens]	5	86.1	LIMA1	10.866	0.96932	762,423	8,284,183
High	EAW71838.1	myosin, heavy polypeptide 14, isoform CRA_a [Homo sapiens]	18	243.9	MYH14	10.858	0.96684	1,168,867	12,691,864
High	NP_079434.3	RUN and FYVE domain-containing protein 1 isoform a [Homo sapiens]	12	79.8	RUFY1	10.826	0.96684	3,034,227	32,848,485
High	AAH02487.1	Tumor susceptibility gene 101 [Homo sapiens]	5	43.9	TSG101	10.791	0.96684	1,725,214	18,617,507
High	AAH50036.1	CSNK2A1 protein [Homo sapiens]	3	45.9	CSNK2A1	10.763	0.99921	490,601	5,280,287
High	AAH16736.1	HNRPF protein [Homo sapiens]	10	45.7	HNRNPF	10.647	0.9704	1,723,000	18,345,488
High	AAI13115.1	Rho-associated, coiled-coil containing protein kinase 1 [Homo sapiens]	1	158	ROCK1	10.602	0.99335	173,503	1,868,815
High	Q14393.2	RecName: Full=Growth arrest-specific protein 6; Short=GAS-6; AltName: Full=AXL re	9	79.6	GAS6	10.6	0.97345	827,853	8,775,474
High	NP_114032.2	heterogeneous nuclear ribonucleoprotein U isoform a [Homo sapiens]	10	90.5	HNRNPU	10.559	0.97225	3,338,153	35,247,724
High	BAG58281.1	unnamed protein product [Homo sapiens]	3	86.9	PANK4	10.552	0.98284	295,304	3,116,134
High	AAI43248.1	TUBGCP2 protein [Homo sapiens]	1	105.4	TUBGCP2	10.55	0.9807	58,799	623,527
High	AAH14775.1	Solute carrier family 25 (mitochondrial carrier; adenine nucleotide translocator), me	15	32.9	SLC25A6	10.532	0.97345	1,836,252	19,340,198
High	BAD92022.1	phosphoribosylglycinamide formyltransferase, phosphoribosylglycinamide syntheta	29	112.1	GART	10.499	0.97439	9,323,052	97,885,288
High	AAA36025.1	90kDa heat shock protein [Homo sapiens]	39	83.2	HSP90AB1	10.461	0.97589	19,580,462	204,833,038
High	AAT52215.1	cell proliferation-inducing protein 53 [Homo sapiens]	5	104.1	NEDD4	10.439	0.99335	194,063	1,896,187
High	BAG51276.1	unnamed protein product [Homo sapiens]	7	28.2	YWHAG	10.406	0.99056	454,603	4,730,484
High	BAG52597.1	unnamed protein product [Homo sapiens]	4	32.4	ATP2A1	10.396	0.99722	147,780	1,536,330
High	BAD96916.1	proteasome 26S non-ATPase subunit 11 variant, partial [Homo sapiens]	1	47.5	PSMD11	10.368	0.98897	188,878	1,957,422
High	AAI21795.1	Valosin-containing protein [Homo sapiens]	25	89.3	VCP	10.348	0.98042	11,280,931	116,737,205
High	AAH62638.1	SNX5 protein, partial [Homo sapiens]	4	47.4	SNX5	10.343	0.98044	1,430,508	14,796,097
High	AAA36399.1	phosphatase 2A regulatory subunit [Homo sapiens]	25	65.2	PPP2R1A	10.338	0.98052	13,423,847	138,771,137
High	XP_011517184.1	PREDICTED: DENN domain-containing protein 1A isoform X1 [Homo sapiens]	3	119.4	DENND1A	10.319	0.98056	5,753,645	59,369,040
High	NP_001138473.1	serine/threonine-protein kinase Nek6 isoform 1 [Homo sapiens]	1	39.8	NEK6	10.272	0.97907	421,015	4,324,462
High	EAW97759.1	KIAA1033, isoform CRA_b [Homo sapiens]	3	142.5	KIAA1033;	10.264	0.98118	273,552	2,807,819
High	EAW95621.1	lysyl-tRNA synthetase, isoform CRA_c [Homo sapiens]	5	71.9	KARS	10.251	0.98198	1,463,399	15,001,651
High	EAW75608.1	dolichyl-phosphate mannosyltransferase polypeptide 1, catalytic subunit, isoform C	2	35.8	DPM1	10.234	0.97648	256,774	2,627,699
High	BAD96295.1	TATA binding protein interacting protein 49 kDa variant, partial [Homo sapiens]	14	50.2	RUVBL1	10.226	0.98237	4,571,019	46,743,284
High	BAO19692.1	FGFR2-AHCYL1 fusion kinase protein [Homo sapiens]	7	130.8	FGFR2	10.173	0.98284	1,297,666	13,201,722
High	AAC16004.1	autosomal dominant polycystic kidney disease type II protein [Homo sapiens]	4	109.7	PKD2	10.165	0.98804	825,035	8,386,603
High	NP_006741.1	beta-2-syntrophin [Homo sapiens]	5	57.9	SNTB2	10.129	0.98499	1,415,702	14,339,040
High	EAL23965.1	KIAA0415 gene product [Homo sapiens]	2	164.6	AP5Z1	10.109	0.9704	344,243	3,480,122
High	NP_006588.1	heat shock cognate 71 kDa protein isoform 1 [Homo sapiens]	40	70.9	HSPA8	10.074	0.98761	39,785,303	400,804,997
High	NP_001311230.1	dolichyl-diphosphooligosaccharide--protein glycosyltransferase subunit 2 isoform 4	10	73	RPN2	10.06	0.98804	5,444,484	54,770,693
High	BAD92832.1	DEAD box polypeptide 17 isoform p82 variant, partial [Homo sapiens]	16	81	DDX17	10.044	0.98845	2,336,066	23,463,884
High	EAW62546.1	annexin A1, isoform CRA_b [Homo sapiens]	12	40.2	ANXA1	10.034	0.98852	4,644,160	46,599,338
High	AAG39278.1	MSTP017 [Homo sapiens]	2	48.1	BZW2	10.011	0.97345	247,193	2,474,541
High	NP_653173.1	TBC1 domain family member 2B isoform a [Homo sapiens]	2	109.8	TBC1D2B	9.972	0.98011	200,731	2,001,618
High	EAW96654.1	keratin 8, isoform CRA_d [Homo sapiens]	45	62.1	KRT8	9.969	0.98966	39,985,195	398,602,093
High	BAD92220.1	DEAD/H (Asp-Glu-Ala-Asp/His) box polypeptide 3 variant, partial [Homo sapiens]	19	74.5	DDX3X	9.899	0.99289	6,032,275	59,713,143
High	EAW49375.1	chromosome 10 open reading frame 119, isoform CRA_a [Homo sapiens]	2	73.5	MCMBP	9.886	0.96932	375,932	3,716,403
High	AAA72127.1	CTP:phosphocholine cytidylyltransferase [Homo sapiens]	5	41.7	PCYT1A	9.868	0.99335	1,053,785	10,398,870
High	EAW84427.1	GIPC PDZ domain containing family, member 1, isoform CRA_c [Homo sapiens]	2	36.7	GIPC1	9.794	0.9757	530,031	5,191,059
High	NP_060684.4	leucine-rich repeat-containing protein 1 [Homo sapiens]	16	59.2	LRRC1	9.781	0.99484	3,219,895	31,494,997
High	AAA03427.1	54 kDa protein [Homo sapiens]	7	54.2	NONO	9.777	0.99484	1,493,098	14,598,448
High	AAB67978.1	helicase [Homo sapiens]	3	137.7	SKIV2L	9.767	0.9704	295,568	2,886,910
High	NP_705694.2	keratin, type I cytoskeletal 13 isoform a [Homo sapiens]	7	49.5	KRT13	9.756	0.99605	1,762,957	17,198,534
High	XP_016884719.1	PREDICTED: peroxiredoxin-4 isoform X1 [Homo sapiens]	6	39.7	PRDX4	9.724	0.99733	1,127,760	10,966,422
High	AAH08674.1	ERO1-like (S. cerevisiae) [Homo sapiens]	12	54.4	ERO1L; ER	9.669	0.99921	5,999,184	58,003,412
High	BAA11829.1	collagen binding protein 2 [Homo sapiens]	10	46.5	SERPINH1	9.664	0.99921	4,095,834	39,582,209
High	XP_016872416.1	PREDICTED: phosphotriesterase-related protein isoform X1 [Homo sapiens]	4	41.4	PTER	9.639	0.99921	1,583,604	15,264,813
High	XP_005269076.1	PREDICTED: 5'-AMP-activated protein kinase subunit gamma-1 isoform X1 [Homo sa	4	41.4	PRKAG1	9.602	0.9704	493,316	4,736,999
High	NP_001531.1	heat shock protein beta-1 [Homo sapiens]	4	22.8	HSPB1	9.597	0.99778	2,316,828	22,233,597
High	AAH68454.1	PDCD6IP protein [Homo sapiens]	24	96.8	PDCD6IP	9.589	0.99764	8,965,211	85,969,171
High	XP_005246300.1	PREDICTED: lanC-like protein 1 isoform X1 [Homo sapiens]	7	46.5	LANCL1	9.583	0.9974	2,498,035	23,938,595
High	NP_002270.1	keratin, type I cuticular Ha3-II [Homo sapiens]	9	46.2	KRT33B	9.56	0.99613	2,336,897	22,340,136
High	CAG30732.1	keratin b20 [Homo sapiens]	21	50.5	KRT80	9.547	0.99605	5,330,809	50,890,814
High	XP_016858234.1	PREDICTED: translation initiation factor eIF-2B subunit gamma isoform X1 [Homo sa	1	51.1	EIF2B3	9.542	0.97988	114,232	1,083,739
High	NP_001311048.1	cytoplasmic FMR1-interacting protein 1 1287 isoform c [Homo sapiens]	30	148.6	CYFIP1	9.522	0.99484	4,137,108	39,392,592
High	NP_001238833.1	DCC-interacting protein 13-beta isoform 2 [Homo sapiens]	5	75.1	APPL2	9.514	0.99335	903,043	8,591,802
High	AAH47240.1	HEAT repeat containing 2 [Homo sapiens]	3	93.5	HEATR2; [	9.497	0.96625	334,697	3,178,524
High	AAA85135.1	desmoplakin I [Homo sapiens]	42	331.6	DSP	9.496	0.99451	9,377,006	89,044,226
High	NP_000935.1	serine/threonine-protein phosphatase 2B catalytic subunit alpha isoform isoform 1	4	58.7	PPP3CA	9.486	0.99484	1,116,078	10,586,606
High	NP_001026866.1	phospholipase D3 [Homo sapiens]	4	54.7	PLD3	9.479	0.9936	1,313,287	12,449,157
High	NP_001308288.1	nuclear receptor-binding protein isoform 2 [Homo sapiens]	1	60.8	NRBP1	9.47	0.98198	147,478	862,642
High	ABC40730.1	heat shock 90kDa protein 1, alpha [Homo sapiens]	30	98.1	HSP90AA1	9.462	0.99335	16,479,804	155,938,383
High	BAG35276.1	unnamed protein product [Homo sapiens]	1	55.9	IFIT5	9.458	0.99778	48,760	460,637
High	CAH18438.1	hypothetical protein [Homo sapiens]	18	155.1	TNS3	9.451	0.99335	5,074,952	47,962,391
High	AAH00151.1	Chromosome 22 open reading frame 28 [Homo sapiens]	12	55.2	C22orf28;	9.44	0.99335	1,050,336	9,915,208
High	BAA07919.1	26S proteasome subunit p45 [Homo sapiens]	4	45.6	PSMC5	9.428	0.96684	484,860	4,571,022
High	BAB91138.1	CLCP1 [Homo sapiens]	2	85	DCBLD2	9.376	0.96625	445,727	4,179,335
High	XP_006723869.1	PREDICTED: uridine-cytidine kinase-like 1 isoform X1 [Homo sapiens]	1	62.4	UCKL1	9.376	0.96684	223,159	2,092,447
High	NP_006532.2	glutaredoxin-3 isoform 1 [Homo sapiens]	1	37.4	GLRX3	9.331	0.99399	37,039	348,104
High	NP_004528.1	nucleosome assembly protein 1-like 1 isoform 1 [Homo sapiens]	5	45.3	NAP1L1	9.324	0.98897	1,892,379	17,645,125
High	CCQ43957.1	alternative protein LOC442572 [Homo sapiens]	1	10.1		9.315	0.96684	498,351	4,649,724
High	NP_001291278.1	cAMP-dependent protein kinase catalytic subunit alpha isoform 3 [Homo sapiens]	1	49.1	PRKACA	9.275	0.96625	214,386	1,995,673
High	BAA37142.1	Acyl-CoA synthetase 3 [Homo sapiens]	1	80.3	ACSL3	9.256	0.96684	181,250	1,639,296
High	CAB66747.1	hypothetical protein [Homo sapiens]	2	67.6	CPSF3L; IN	9.247	0.96146	371,214	3,432,443
High	EAX02330.1	ribonuclease/angiogenin inhibitor 1, isoform CRA_a [Homo sapiens]	19	49.4	RNH1	9.218	0.98558	10,583,769	97,563,050
High	EAW84148.1	dynamin 2, isoform CRA_e [Homo sapiens]	2	98.2	DNM2	9.189	0.96146	411,829	3,784,323
High	BAG36700.1	unnamed protein product [Homo sapiens]	29	48	KRT18	9.133	0.98242	88,617,068	809,327,106
High	XP_005262538.1	PREDICTED: cullin-4B isoform X1 [Homo sapiens]	2	104.1	CUL4B	9.066	0.96684	138,778	1,258,090
High	NP_001032248.1	acyl-coenzyme A thioesterase 9, mitochondrial isoform a precursor [Homo sapiens]	16	50.8	ACOT9	9.051	0.98118	2,593,020	23,470,551
High	NP_004512.1	kinesin-1 heavy chain [Homo sapiens]	16	109.6	KIF5B	9.047	0.98118	2,915,176	26,374,501
High	AAA35484.1	26S protease (S4) regulatory subunit [Homo sapiens]	5	49.2	PSMC1	8.963	0.97961	1,128,074	10,111,494
High	BAH14104.1	unnamed protein product [Homo sapiens]	1	112.5	RTN3	8.945	0.9568	415,150	3,713,500
High	AAA67526.1	MTHSP75 [Homo sapiens]	23	73.7	HSPA9	8.937	0.97821	12,361,863	110,478,960
High	XP_011531926.1	PREDICTED: sulfatase-modifying factor 1 isoform X1 [Homo sapiens]	1	49.4	SUMF1	8.915	0.96625	165,805	1,478,216
High	NP_055454.1	tetratricopeptide repeat protein 37 [Homo sapiens]	6	175.4	TTC37	8.9	0.97574	1,315,944	11,711,992
High	BAD93116.1	procollagen-lysine, 2-oxoglutarate 5-dioxygenase 2 isoform b variant, partial [Homo	12	90.8	PLOD2	8.896	0.9757	4,864,072	43,270,094
High	NP_742034.1	retinol dehydrogenase 10 [Homo sapiens]	2	38.1	RDH10	8.87	0.96684	798,166	7,079,413

High	BAD97316.1	dihydrolipoamide S-acetyltransferase (E2 component of pyruvate dehydrogenase co	4	69	DLAT	8.843	0.96863	796,113	7,040,090
High	XP_016879314.1	PREDICTED: mitochondrial Rho GTPase 2 isoform X1 [Homo sapiens]	2	73	RHOT2	8.842	0.96146	252,436	2,232,001
High	NP_001681.2	V-type proton ATPase catalytic subunit A [Homo sapiens]	19	68.3	ATP6V1A	8.815	0.9721	6,968,032	61,422,226
High	BAG59267.1	unnamed protein product [Homo sapiens]	9	54.8	TOM1	8.806	0.97144	2,045,139	18,008,561
High	XP_016856050.1	PREDICTED: alpha-taxilin isoform X1 [Homo sapiens]	3	65.5	TXLNA	8.803	0.96146	215,762	1,897,378
High	CAI42843.1	ERGIC and golgi 3, partial [Homo sapiens]	1	44.5	ERGIC3	8.759	0.96625	125,498	1,090,175
High	NP_065132.1	Golgi-associated PDZ and coiled-coil motif-containing protein isoform a [Homo sapi	4	50.5	GOPC	8.757	0.9704	1,239,828	10,856,766
High	EAW63361.1	proline synthetase co-transcribed homolog (bacterial), isoform CRA_b [Homo sapie	3	34	PROSC; PL	8.737	0.9704	1,157,024	10,109,165
High	CAH18231.1	hypothetical protein [Homo sapiens]	3	93.7	CSDE1	8.732	0.9582	616,181	5,380,237
High	AAC39915.1	mitochondrial processing peptidase beta-subunit [Homo sapiens]	3	54.1	PMPCB	8.704	0.96625	804,966	7,006,091
High	XP_005266191.1	PREDICTED: protein transport protein Sec16A isoform X1 [Homo sapiens]	1	254	SEC16A	8.673	0.96625	140,116	1,215,234
High	NP_001127703.1	5'-nucleotidase domain-containing protein 2 isoform 1 [Homo sapiens]	18	64.1	NT5DC2	8.665	0.96867	12,603,383	109,207,947
High	BAG63980.1	unnamed protein product [Homo sapiens]	1	43.4	METTL2B	8.581	0.96444	174,586	1,498,113
High	AAC60648.1	nucleoprotein interactor 1 [Homo sapiens]	2	60.3	KPNA1	8.574	0.93971	376,919	3,239,092
High	NP_776993.1	polyadenylate-binding protein 1 [Bos taurus]	15	70.6	PABPC1	8.539	0.96653	6,822,307	58,254,133
High	EAW89326.1	WW domain binding protein 2, isoform CRA_b [Homo sapiens]	4	29.8	WBP2	8.531	0.96625	1,140,278	9,727,952
High	AAA59553.1	microtubule-associated protein 4 [Homo sapiens]	26	121.1	MAP4	8.521	0.96625	12,733,924	108,508,937
High	BAH14038.1	unnamed protein product [Homo sapiens]	2	46.6	AHSG	8.514	0.95193	546,656	4,465,910
High	XP_005266967.1	PREDICTED: monofunctional C1-tetrahydrofolate synthase, mitochondrial isoform X	7	106.7	MTHFD1L	8.507	0.96625	2,115,675	17,998,656
High	BAD92062.1	poly(rC)-binding protein 2 isoform b variant, partial [Homo sapiens]	7	39.5	PCBP2	8.504	0.96625	3,300,831	28,069,970
High	AAH12514.1	ELP4 protein [Homo sapiens]	2	58.7	ELP4	8.459	0.96146	164,626	1,392,545
High	EAW89820.1	fructosamine 3 kinase, isoform CRA_b [Homo sapiens]	4	50.1	FN3K	8.445	0.96625	1,128,426	9,529,747
High	NP_001995.1	farnesyl pyrophosphate synthase isoform a [Homo sapiens]	1	48.2	FDPS	8.443	0.95789	184,229	1,563,586
High	NP_006657.1	ruvB-like 2 isoform 1 [Homo sapiens]	19	51.1	RUVBL2	8.441	0.96625	8,135,933	68,671,646
High	BAA92005.1	unnamed protein product [Homo sapiens]	11	40.7	RRM2B	8.39	0.96625	3,525,590	29,579,162
High	NP_001316166.1	serine/threonine-protein kinase Nek9 isoform 1 [Homo sapiens]	18	108.4	NEK9	8.346	0.96625	14,930,474	124,608,959
High	ABR25253.1	ubiquitin-activating enzyme 6 [Homo sapiens]	22	117.9	UBA6	8.303	0.96625	7,777,249	64,572,681
High	AAD41240.1	beta-cop homolog [Homo sapiens]	9	107.1	COPB1	8.252	0.96562	2,291,924	18,912,045
High	EAW89818.1	fructosamine-3-kinase-related protein, isoform CRA_b [Homo sapiens]	13	38.1	FN3KRP	8.227	0.96444	23,795,722	195,756,469
High	AAB40653.1	MAP kinase 3c [Homo sapiens]	4	39.9	MAP2K3	8.201	0.93635	235,673	1,932,750
High	XP_016862105.1	PREDICTED: 6-phosphofructo-2-kinase/fructose-2,6-bisphosphatase 4 isoform X4 [H	1	52.7	PFKFB4	8.183	0.9539	166,917	1,365,804
High	XP_005268923.1	PREDICTED: keratin, type II cuticular Hb6 isoform X1 [Homo sapiens]	2	62	KRT86	8.154	0.93392	394,213	3,214,579
High	XP_011536999.1	PREDICTED: signal transducer and activator of transcription 2 isoform X1 [Homo sap	2	98.7	STAT2	8.125	0.93392	383,881	3,081,639
High	EAW82829.1	acyltransferase like 1 [Homo sapiens]	11	60.2	LPCAT2	8.122	0.96146	5,765,010	46,821,408
High	NP_115679.2	hydroxysteroid dehydrogenase-like protein 2 isoform 1 [Homo sapiens]	7	45.4	HSDL2	8.109	0.96127	2,360,035	19,138,508
High	BAD96198.1	chaperonin containing TCP1, subunit 7 (eta) variant, partial [Homo sapiens]	18	59.3	CCT7	8.058	0.96012	5,313,722	42,818,724
High	EAX10959.1	DnaJ (Hsp40) homolog, subfamily C, member 10, isoform CRA_a [Homo sapiens]	28	94.5	DNAJC10	8.052	0.9596	1,890,312	15,220,139
High	NP_059447.2	major vault protein isoform 1 [Homo sapiens]	62	99.3	MVP	8.03	0.9582	167,031,368	1,341,300,860
High	AAA96830.1	aldehyde dehydrogenase [Homo sapiens]	15	57.2	ALDH1B1	7.986	0.95614	6,118,100	48,861,085
High	AAD45919.2	MAGUK protein VAM-1 [Homo sapiens]	6	61.1	MPP6	7.931	0.95244	1,690,689	13,408,035
High	XP_016859680.1	PREDICTED: septin-2 isoform X3 [Homo sapiens]	11	49.4	SEPT2	7.917	0.95193	5,224,632	41,363,963
High	NP_997226.2	RING finger protein 214 isoform 1 [Homo sapiens]	1	77.6	RNF214	7.915	0.94349	114,577	906,847
High	BAD96555.1	asparaginyl-tRNA synthetase variant, partial [Homo sapiens]	6	62.9	NARS	7.904	0.95139	1,561,571	12,342,843
High	Q6ZSR9.2	RecName: Full=Uncharacterized protein FLJ45252	1	38		7.902	0.95789	85,617	674,243
High	CCB78975.1	MHC class I antigen, partial [Homo sapiens]	6	38.1	HLA-A	7.888	0.93392	561,153	4,426,292
High	NP_004604.2	protein-glutamine gamma-glutamyltransferase 2 isoform a [Homo sapiens]	14	77.3	TGM2	7.873	0.94942	6,530,540	51,414,975
High	CAE45708.1	hypothetical protein, partial [Homo sapiens]	2	51.2	SUCLA2	7.871	0.93392	558,796	4,398,286
High	EAX00246.1	ras homolog gene family, member Q, isoform CRA_a, partial [Homo sapiens]	1	28.5	RHOQ	7.82	0.93635	149,589	1,168,986
High	NP_115722.1	programmed cell death protein 2-like [Homo sapiens]	1	39.4	PDCD2L	7.817	0.94006	106,294	830,936
High	EAX02376.1	EPS8-like 2, isoform CRA_b [Homo sapiens]	11	85.1	EPS8L2	7.812	0.94494	4,161,024	32,504,100
High	BAB13991.1	unnamed protein product [Homo sapiens]	2	72.6	TBC1D17	7.784	0.93392	409,681	2,704,514
High	ABB01006.1	heat shock protein 60 [Homo sapiens]	21	61.2	HSPD1	7.781	0.94327	10,565,657	82,211,308
High	AAI07805.1	PSMC3 protein, partial [Homo sapiens]	5	55	PSMC3	7.765	0.94067	931,286	7,231,650
High	BAB14426.1	unnamed protein product [Homo sapiens]	1	74	FIGNL1	7.741	0.93392	463,669	3,589,484
High	BAA00845.1	VLA-3 alpha subunit, partial [Homo sapiens]	4	113.4	ITGA3	7.696	0.93846	1,936,505	14,903,544
High	NP_659471.1	torsin-1A-interacting protein 2 isoform b [Homo sapiens]	1	51.2	TOR1AIP2	7.694	0.93392	455,699	3,506,199
High	BAD92969.1	replication protein A1, 70kDa variant, partial [Homo sapiens]	4	69.4	RPA1	7.681	0.93392	574,149	4,410,016
High	XP_005274822.1	PREDICTED: myotubularin-related protein 1 isoform X1 [Homo sapiens]	1	78.7	MTMR1	7.665	0.93392	263,513	1,872,361
High	AAH60792.1	ACBD3 protein [Homo sapiens]	1	60.6	ACBD3	7.654	0.93392	345,640	2,645,591
High	BAD92525.1	calcium/calmodulin-dependent protein kinase II delta isoform 1 variant, partial [Hoi	11	58.8	CAMK2D	7.645	0.93635	3,287,643	25,133,812
High	NP_005372.2	nucleolin [Homo sapiens]	22	76.6	NCL	7.637	0.93635	17,752,838	135,576,981
High	AAC16046.1	FIP2 [Homo sapiens]	3	65.9	OPTN	7.613	0.93392	172,744	1,315,039
High	CAI45986.1	hypothetical protein [Homo sapiens]	5	50.3	ACADM	7.596	0.9353	1,502,149	11,409,597
High	EAW77320.1	hCG2001986, isoform CRA_b [Homo sapiens]	12	54.9		7.589	0.9353	7,249,705	55,018,208
High	NP_056415.1	monoacylglycerol lipase ABHD12 isoform b [Homo sapiens]	5	45.5	ABHD12	7.589	0.9353	1,494,085	11,338,857
High	AAH32540.1	F-box protein 22 [Homo sapiens]	10	44.4	FBXO22	7.569	0.93472	3,973,051	30,072,052
High	NP_001317366.1	tyrosine-protein phosphatase non-receptor type 11 isoform 3 [Homo sapiens]	20	68.4	PTPN11	7.564	0.93472	10,212,498	77,249,731
High	XP_006712562.1	PREDICTED: type I inositol 3,4-bisphosphate 4-phosphatase isoform X1 [Homo sapie	4	111.6	INPP4A	7.559	0.93392	333,487	2,520,769
High	BAD92968.1	Glucan, branching enzyme 1 variant, partial [Homo sapiens]	8	86.1	GBE1	7.547	0.93449	2,458,383	18,552,762
High	XP_005245715.1	PREDICTED: ubiquitin-associated protein 2-like isoform X1 [Homo sapiens]	1	117.6	UBAP2L	7.531	0.93392	192,661	1,450,856
High	AAH13878.1	Thimet oligopeptidase 1 [Homo sapiens]	8	78.8	THOP1	7.525	0.93392	1,876,935	14,124,114
High	EAW83689.1	filamin C, gamma (actin binding protein 280), isoform CRA_a, partial [Homo sapiens	21	292	FLNC	7.524	0.93392	2,832,349	21,311,849
High	ADP90080.1	mitochondrial dynamin-like 120 kDa protein [Homo sapiens]	47	115.8	OPA1	7.515	0.93392	48,571,131	364,991,113
High	EAW86495.1	phosphofructokinase, platelet, isoform CRA_a [Homo sapiens]	25	92.7	PFKP	7.512	0.93392	13,933,280	104,667,923
High	BAD93045.1	adaptor-related protein complex 1, mu 1 subunit variant, partial [Homo sapiens]	3	53.2	AP1M1	7.475	0.93392	444,658	3,323,706
High	EAW52854.1	kin of IRRE like (Drosophila), isoform CRA_b [Homo sapiens]	1	88.5	KIRREL; KI	7.473	0.93392	398,111	2,974,937
High	BAG61736.1	unnamed protein product [Homo sapiens]	1	106	FAM120B	7.472	0.93392	244,217	1,823,498
High	NP_001157789.1	filamin-B isoform 1 [Homo sapiens]	51	281.5	FLNB	7.434	0.93392	15,768,663	117,225,525
High	NP_005338.1	78 kDa glucose-regulated protein precursor [Homo sapiens]	34	72.3	HSPA5	7.407	0.93392	55,434,208	410,595,245
High	AAH22281.1	Tripartite motif-containing 22 [Homo sapiens]	6	56.9	TRIM22	7.394	0.93392	3,856,072	28,511,148
High	NP_000242.1	DNA mismatch repair protein Msh2 isoform 1 [Homo sapiens]	12	104.7	MSH2	7.385	0.93392	2,706,257	19,985,359
High	XP_009240803.1	PREDICTED: cytohesin-3 [Pongo abelii]	2	46.3	CYTH3	7.364	0.93392	395,437	2,912,113
High	NP_001290043.1	tubulin alpha-1C chain isoform a [Homo sapiens]	28	57.7	TUBA1C	7.354	0.93392	275,705	2,027,590
High	BAF84522.1	unnamed protein product [Homo sapiens]	3	35.1	C11orf54	7.268	0.93392	1,000,277	7,269,616
High	XP_006715869.1	PREDICTED: septin-7 isoform X2 [Homo sapiens]	11	52	SEPT7	7.263	0.93392	5,760,051	41,837,027
High	EAX09512.1	cystathionine-beta-synthase, isoform CRA_c [Homo sapiens]	4	62.4	CBS	7.23	0.93392	705,997	5,104,286
High	AAD21815.1	HSP70-2 [Homo sapiens]	33	70	HSPA1B	7.227	0.93392	3,471,405	25,087,891
High	BAG58692.1	unnamed protein product [Homo sapiens]	3	112.9		7.216	0.93392	888,738	6,413,510
High	AAP97210.1	lysophospholipase LPL-I [Homo sapiens]	2	24.4	LYPLA2	7.199	0.93392	673,343	4,847,520
High	NP_001104026.1	filamin-A isoform 2 [Homo sapiens]	89	280.6	FLNA	7.187	0.93392	63,364,217	455,402,980
High	BAF83227.1	unnamed protein product [Homo sapiens]	15	57.9	CCT4	7.17	0.93392	7,458,740	53,481,757
High	AAH00441.2	JUP protein, partial [Homo sapiens]	10	85.6	JUP	7.138	0.93392	1,606,093	11,464,934
High	BAG37094.1	unnamed protein product [Homo sapiens]	18	100.7	AMPD2	7.132	0.93392	13,309,143	94,924,454
High	XP_011521615.1	PREDICTED: alpha-ketoglutarate-dependent dioxygenase FTO isoform X1 [Homo sap	3	59.4	FTO	7.119	0.93392	1,111,621	7,914,152
High	EAW70563.1	X-ray repair complementing defective repair in Chinese hamster cells 5 (double-stra	13	93.5	XRCC5	7.111	0.93392	5,346,803	38,021,502

High	CAA53972.1	RNase L inhibitor [Homo sapiens]	1	67.5	ABCE1	7.083	0.93392	219,887	1,555,524
High	EAW67101.1	cullin 5, isoform CRA_a [Homo sapiens]	13	97.2	CUL5	7.051	0.93392	3,480,128	24,538,922
High	XP_005249419.1	PREDICTED: valine--tRNA ligase isoform X1 [Homo sapiens]	10	140.5	VARS	7.033	0.93392	1,731,566	12,177,511
High	NP_001171846.1	ethanolamine-phosphate cytidylyltransferase isoform 1 [Homo sapiens]	1	45.6	PCYT2	7.009	0.93392	274,785	1,925,906
High	AAA51915.1	common acute lymphoblastic leukemia antigen precursor [Homo sapiens]	1	85.7	MME	7	0.93392	230,123	1,611,218
High	BAA95163.2	hVPS11 [Homo sapiens]	3	107.7	VPS11	6.997	0.93392	675,811	4,728,955
High	AAA52712.1	insulin-degrading enzyme [Homo sapiens]	3	117.9	IDE	6.997	0.93392	410,688	2,873,558
High	BAG61974.1	unnamed protein product [Homo sapiens]	13	56.1	EEF1G	6.979	0.93392	8,648,920	60,359,261
High	AAD34145.1	CGI-150 protein [Homo sapiens]	11	55	GLOD4	6.936	0.93392	6,771,125	46,961,697
High	XP_005262745.1	PREDICTED: protein transport protein Sec24B isoform X1 [Homo sapiens]	8	140.5	SEC24B	6.933	0.93392	1,622,882	11,251,605
High	XP_005259725.1	PREDICTED: far upstream element-binding protein 2 isoform X1 [Homo sapiens]	12	76.6	KHSRP	6.921	0.93392	5,664,256	39,200,429
High	EAW65917.1	sorting nexin 6, isoform CRA_d [Homo sapiens]	5	50.1	SNX6	6.918	0.93392	2,780,552	19,236,537
High	Q96ME1.2	RecName: Full=F-box/LRR-repeat protein 18; AltName: Full=F-box and leucine-rich r	1	88.3	FBXL18	6.908	0.93392	309,954	2,141,291
High	AAD00728.1	hepatocellular carcinoma associated protein [Homo sapiens]	2	64.9	MAGED2	6.894	0.93392	376,190	2,593,571
High	NP_001092006.1	ubiquitin carboxyl-terminal hydrolase 5 isoform 1 [Homo sapiens]	18	95.7	USP5	6.87	0.93392	7,272,187	49,958,351
High	EAW49364.1	bromodomain and WD repeat domain containing 2, isoform CRA_b, partial [Homo s	3	137.1	WDR11	6.856	0.93392	594,602	4,076,651
High	NP_995314.1	nck-associated protein 1 isoform 2 [Homo sapiens]	34	129.4	NCKAP1	6.827	0.93392	14,002,039	95,595,652
High	NP_001010853.1	peptidase M20 domain-containing protein 2 [Homo sapiens]	6	47.7	PM20D2	6.826	0.93392	2,119,502	14,467,118
High	NP_008921.1	wiskott-Aldrich syndrome protein family member 2 isoform 1 [Homo sapiens]	6	54.3	WASF2	6.821	0.93392	1,879,933	12,822,472
High	AAH01705.1	Serine active site containing 1 [Homo sapiens]	1	74.1	SERAC1	6.786	0.93392	118,632	804,985
High	ADZ73084.1	MHC class I antigen, partial [Homo sapiens]	7	21.1	HLA-A	6.784	0.93392	170,629	1,176,811
High	CAA23753.1	unnamed protein product [Homo sapiens]	16	69.3	ALB	6.734	0.93392	1,260,649	8,489,481
High	2B5L_A	Chain A, Crystal Structure Of Ddb1 In Complex With Simian Virus 5 V Protein	22	126.9		6.725	0.93392	9,303,631	62,562,950
High	NP_056289.2	deoxynucleoside triphosphate triphosphohydrolase SAMHD1 [Homo sapiens]	11	72.2	SAMHD1	6.715	0.93392	2,580,609	17,328,806
High	BAA96023.1	KIAA1499 protein, partial [Homo sapiens]	1	73.7	NPLOC4	6.693	0.93392	139,576	940,122
High	EAL24101.1	family with sequence similarity 40, member B [Homo sapiens]	1	101.7	STRIP2	6.688	0.93392	244,747	1,636,954
High	AAL84159.1	CD109 [Homo sapiens]	2	161.6	CD109	6.658	0.93392	1,342,548	8,938,026
High	AAH17801.1	BPNT1 protein [Homo sapiens]	1	35.7	BPNT1	6.645	0.93392	284,187	1,888,320
High	NP_001460.1	X-ray repair cross-complementing protein 6 isoform 1 [Homo sapiens]	16	69.8	XRCC6	6.596	0.93392	9,730,057	64,175,965
High	XP_016884163.1	PREDICTED: 2-amino-3-ketobutyrate coenzyme A ligase, mitochondrial isoform X1 [	1	56.3	GCAT	6.594	0.93392	146,483	964,104
High	EAW76471.1	thyroid hormone receptor interactor 6, isoform CRA_a, partial [Homo sapiens]	4	57.5	TRIP6	6.587	0.93392	1,042,356	6,865,499
High	ABO32290.1	TDP43 [Homo sapiens]	1	45	TARDBP	6.584	0.93392	410,060	2,699,968
High	NP_068751.4	synembryn-A isoform 1 [Homo sapiens]	4	60.3	RIC8A	6.579	0.93392	897,487	5,904,147
High	BAG64781.1	unnamed protein product [Homo sapiens]	1	17.6	IBA57	6.579	0.93392	396,097	2,605,819
High	AAD09325.1	ATP sulfurylase/APS kinase [Homo sapiens]	6	70.8	PAPSS1	6.557	0.93392	2,525,136	16,557,523
High	BAF84075.1	unnamed protein product [Homo sapiens]	8	92.2	MALT1	6.552	0.93392	1,647,429	10,793,990
High	XP_011511565.1	PREDICTED: GMP synthase [glutamine-hydrolyzing] isoform X1 [Homo sapiens]	20	77.4	GMPS	6.542	0.93392	9,025,231	59,046,281
High	XP_016878628.1	PREDICTED: copine-7 isoform X2 [Homo sapiens]	18	70.7	CPNE7	6.536	0.93392	16,040,224	104,831,221
High	BAG70037.1	T-complex protein 1 subunit beta [Homo sapiens]	18	57.4	CCT2	6.486	0.93392	6,314,765	40,960,619
High	BAD92471.1	Isoleucyl-tRNA synthetase, cytoplasmic variant, partial [Homo sapiens]	20	146.3	IARS	6.467	0.93392	9,802,375	63,387,567
High	XP_011530070.1	PREDICTED: palladin isoform X1 [Homo sapiens]	21	159.6	PALLD	6.451	0.93392	1,360,872	8,778,405
High	BAG64112.1	unnamed protein product [Homo sapiens]	2	57.3	ATG4B	6.431	0.91583	485,852	3,124,399
High	NP_004334.1	calreticulin precursor [Homo sapiens]	1	48.1	CALR	6.429	0.93392	332,039	2,134,650
High	XP_016885266.1	PREDICTED: ubiquitin-like modifier-activating enzyme 1 isoform X1 [Homo sapiens]	15	123.1	UBA1	6.428	0.93392	6,316,960	40,607,701
High	NP_000684.2	aldehyde dehydrogenase family 1 member A3 isoform 1 [Homo sapiens]	26	56.1	ALDH1A3	6.423	0.93392	34,034,541	218,605,468
High	BAG54627.1	unnamed protein product [Homo sapiens]	1	94.1	AP1G1	6.418	0.91583	393,977	2,529,656
High	XP_005261192.1	PREDICTED: ATP-dependent 6-phosphofructokinase, liver type isoform X3 [Homo sa	16	87.6	PFKL	6.387	0.93392	4,555,409	29,095,562
High	NP_001303249.1	procollagen-lysine,2-oxoglutarate 5-dioxygenase 1 isoform 1 precursor [Homo sapie	15	88.2	PLOD1	6.374	0.93392	4,697,686	29,943,142
High	BAB13971.1	unnamed protein product [Homo sapiens]	8	77.2	TBC1D15	6.366	0.93392	2,908,215	18,512,523
High	XP_011543800.1	PREDICTED: SH3 domain-containing kinase-binding protein 1 isoform X1 [Homo sap	3	77.9	SH3KBP1	6.363	0.93392	981,239	6,244,084
High	AAC42010.1	pyruvate dehydrogenase kinase [Homo sapiens]	2	46.2	PDK2	6.358	0.92036	369,670	2,350,329
High	NP_055955.1	tubulin--tyrosine ligase-like protein 12 [Homo sapiens]	7	74.4	TTLL12	6.341	0.93392	1,684,117	10,679,203
High	XP_016872625.1	PREDICTED: NADP-dependent malic enzyme, mitochondrial isoform X1 [Homo sapie	1	80.4	ME3	6.337	0.93392	175,103	1,109,569
High	NP_001184222.1	dihydropyrimidinase-related protein 2 isoform 1 [Homo sapiens]	31	73.5	DPYSL2	6.274	0.93392	52,240,524	327,745,425
High	BAG51068.1	unnamed protein product [Homo sapiens]	3	73.3	OGFR	6.26	0.92439	714,541	4,472,971
High	NP_055682.1	kelch repeat and BTB domain-containing protein 11 [Homo sapiens]	21	65.7	KBTBD11	6.258	0.93375	30,148,138	188,659,636
High	EAW67681.1	FAD-dependent oxidoreductase domain containing 1, isoform CRA_a [Homo sapien	1	54.5	FOXRED1	6.243	0.93392	104,328	642,373
High	XP_016882575.1	PREDICTED: epidermal growth factor receptor substrate 15-like 1 isoform X1 [Homo	2	101.3	EPS15L1	6.22	0.91491	440,847	2,742,095
High	AAH03656.1	Minichromosome maintenance complex component 5 [Homo sapiens]	11	82.3	MCM5	6.206	0.92746	3,705,658	22,997,819
High	NP_055045.2	signal recognition particle subunit SRP68 isoform 1 [Homo sapiens]	8	70.7	SRP68	6.196	0.92623	2,012,427	12,468,162
High	NP_003355.1	uridine phosphorylase 1 isoform a [Homo sapiens]	10	33.9	UPP1	6.188	0.92566	7,182,532	44,445,745
High	BAC11526.1	unnamed protein product [Homo sapiens]	16	47.6	TXNDC5	6.185	0.92541	49,767,284	307,812,468
High	NP_065143.2	manganese-transporting ATPase 13A1 [Homo sapiens]	5	132.9	ATP13A1	6.185	0.92541	1,633,049	10,101,182
High	AAD37491.1	ribonucleotide reductase M1 subunit [Homo sapiens]	14	90	RRM1	6.172	0.9251	6,312,657	38,958,760
High	CAA44721.1	vacuolar isoform 2 of H+ATPase Mr 56,000 subunit [Homo sapiens]	15	56.5	ATP6V1B2	6.158	0.92464	6,234,574	38,392,308
High	NP_002824.1	tyrosine-protein phosphatase non-receptor type 9 [Homo sapiens]	2	68	PTPN9	6.152	0.93373	236,159	1,452,875
High	XP_005265573.1	PREDICTED: calcium/calmodulin-dependent protein kinase type 1 isoform X1 [Homo	1	46	CAMK1	6.141	0.92246	266,571	1,631,532
High	NP_061185.1	protein RCC2 [Homo sapiens]	15	56	RCC2	6.114	0.92082	8,969,155	54,836,218
High	AAH50341.1	Cullin-associated and neddylation-dissociated 1 [Homo sapiens]	24	136.3	CAND1	6.065	0.91583	9,596,054	58,204,866
High	NP_001900.1	cathepsin D preproprotein [Homo sapiens]	14	44.5	CTSD	6.059	0.91583	71,961,812	436,044,788
High	XP_016880042.1	PREDICTED: acetyl-CoA carboxylase 1 isoform X1 [Homo sapiens]	32	270.5	ACACA	6.048	0.91491	13,326,823	80,597,201
High	BAG09933.1	palladin, partial [synthetic construct]	18	73.2		6.046	0.90216	433,036	2,625,478
High	BAG50859.1	unnamed protein product [Homo sapiens]	5	111	SEC23IP	6.022	0.90671	789,608	4,755,270
High	CAG38740.1	PCNA [Homo sapiens]	2	28.7	PCNA	5.974	0.89826	844,165	5,043,075
High	AAH09503.2	GSPT1 protein [Homo sapiens]	14	68.4	GSPT1	5.971	0.91181	6,926,085	41,357,723
High	NP_001118.3	AP-1 complex subunit beta-1 isoform a [Homo sapiens]	27	104.5	AP1B1	5.966	0.91181	1,353,113	8,072,404
High	CAH18166.1	hypothetical protein [Homo sapiens]	8	42.9	PRKAR1A	5.959	0.91491	1,128,341	6,724,218
High	NP_065796.1	alanine--tRNA ligase, mitochondrial [Homo sapiens]	2	107.3	AARS2	5.957	0.89991	450,981	2,686,651
High	EAW90617.1	skeletal muscle and kidney enriched inositol phosphatase, isoform CRA_d [Homo sa	2	51.1	INPP5K	5.946	0.89051	551,747	3,280,944
High	BAG62969.1	unnamed protein product [Homo sapiens]	4	56.3	SHMT2	5.941	0.90616	1,486,040	8,828,412
High	BAD92636.1	glycerol-3-phosphate dehydrogenase 2 (mitochondrial) variant, partial [Homo sapie	30	80.9	GPD2	5.93	0.90671	17,555,661	104,113,392
High	NP_001124151.1	eukaryotic translation initiation factor 4E isoform 2 [Homo sapiens]	2	28.8	EIF4E	5.903	0.90437	985,452	5,817,045
High	AAH08751.1	Calpain 1, (mu/I) large subunit [Homo sapiens]	25	81.8	CAPN1	5.902	0.90437	15,080,294	89,003,944
High	CAG46459.1	PSME1, partial [Homo sapiens]	9	28.7	PSME1	5.896	0.90404	5,839,491	34,431,869
High	NP_005198.1	crk-like protein [Homo sapiens]	9	33.8	CRKL	5.885	0.90254	6,666,673	39,230,265
High	NP_001014437.1	cysteine--tRNA ligase, cytoplasmic isoform c [Homo sapiens]	30	94.6	CARS	5.881	0.90216	34,353,637	202,035,203
High	BAD96230.1	glutaminyl-tRNA synthetase variant, partial [Homo sapiens]	20	87.7	QARS	5.866	0.90216	6,407,558	37,586,952
High	NP_064505.1	UDP-glucose:glycoprotein glucosyltransferase 1 precursor [Homo sapiens]	60	177.1	UGGT1	5.797	0.89547	68,244,436	395,615,720
High	BAD93092.1	Calpain 2, large [catalytic] subunit precursor variant, partial [Homo sapiens]	23	83.1	CAPN2	5.791	0.89504	20,949,123	121,321,021
High	AAI06055.1	Aldehyde dehydrogenase 18 family, member A1 [Homo sapiens]	19	87.3	ALDH18A1	5.791	0.89504	11,197,745	64,849,946
High	BAG65435.1	unnamed protein product [Homo sapiens]	20	40.5	PRMT1	5.767	0.89297	34,320,228	197,921,039
High	AAF05708.1	ADP-ribosylation factor binding protein GGA2 [Homo sapiens]	2	67.2	GGA2	5.759	0.88322	873,687	5,031,547
High	XP_011533826.1	PREDICTED: band 4.1-like protein 2 isoform X1 [Homo sapiens]	12	118.4	EPB41L2	5.708	0.88812	3,934,356	22,459,044
High	NP_060713.1	septin-11 isoform 2 [Homo sapiens]	7	49.4	SEPT11	5.684	0.8857	2,379,746	13,525,506
High	XP_016864369.1	PREDICTED: WD repeat-containing protein 1 isoform X1 [Homo sapiens]	7	72	WDR1	5.677	0.88535	2,397,796	13,612,003

High	AAI07750.1	Aspartyl-tRNA synthetase [Homo sapiens]	18	57.1	DARS	5.676	0.88535	9,767,723	55,439,395
High	NP_001129071.1	transforming growth factor beta-2 isoform 1 precursor [Homo sapiens]	1	50.5	TGFB2	5.662	0.88535	350,022	1,981,793
High	EAX07268.1	poly(A) binding protein, cytoplasmic 4 (inducible form), isoform CRA_h [Homo sapie	8	72.4	PABPC4	5.659	0.88115	1,700,159	9,622,003
High	BAG36670.1	unnamed protein product [Homo sapiens]	16	41.8	GMDS	5.624	0.88087	20,902,563	117,561,530
High	EAW68336.1	protein arginine methyltransferase 3, isoform CRA_d [Homo sapiens]	4	63.7	PRMT3	5.593	0.8805	1,795,393	10,041,243
High	XP_011528279.1	PREDICTED: adenylosuccinate lyase isoform X1 [Homo sapiens]	2	59.3	ADSL	5.587	0.87451	456,183	2,548,858
High	EAW69227.1	SH3-domain GRB2-like 1, isoform CRA_a [Homo sapiens]	5	43.5	SH3GL1	5.577	0.8805	2,762,433	15,406,001
High	NP_001952.1	elongation factor 2 [Homo sapiens]	39	95.3	EEF2	5.555	0.87973	46,732,064	259,611,381
High	NP_000593.1	plasminogen activator inhibitor 1 precursor [Homo sapiens]	16	45	SERPINE1	5.553	0.87948	10,506,252	58,341,163
High	NP_001166038.1	myeloid differentiation primary response protein MyD88 isoform 1 [Homo sapiens]	7	35.4	MYD88	5.544	0.87811	2,571,396	14,255,824
High	BAG62968.1	unnamed protein product [Homo sapiens]	5	37.5	CAPZB	5.538	0.87811	3,782,290	20,947,602
High	BAG63427.1	unnamed protein product [Homo sapiens]	2	37.5	RTFDC1	5.511	0.87548	767,456	4,229,263
High	AAH19832.1	B3GAT3 protein, partial [Homo sapiens]	1	37.8	B3GAT3	5.502	0.87583	397,513	2,187,299
High	NP_001087240.1	thioredoxin reductase 1, cytoplasmic isoform 3 [Homo sapiens]	28	70.9	TXNRD1	5.473	0.87342	104,511,544	571,979,691
High	BAA92063.1	unnamed protein product [Homo sapiens]	1	79.7	ABCF3	5.453	0.88446	197,950	1,078,809
High	XP_016858652.1	PREDICTED: SET and MYND domain-containing protein 5 isoform X1 [Homo sapiens	2	48.9	SMYD5	5.422	0.87454	986,644	5,349,334
High	NP_109591.1	leukocyte elastase inhibitor [Homo sapiens]	19	42.7	SERPINB1	5.418	0.86816	16,699,913	90,486,907
High	XP_011524077.1	PREDICTED: serine/threonine-protein phosphatase 4 regulatory subunit 1 isoform X	1	112.2	PPP4R1	5.409	0.88871	132,126	714,636
High	BAD96636.1	isocitrate dehydrogenase 2 (NADP+), mitochondrial precursor variant, partial [Hom	2	50.9	IDH2	5.405	0.85912	552,682	2,987,441
High	XP_011521388.1	PREDICTED: diphosphomevalonate decarboxylase isoform X1 [Homo sapiens]	3	47	MVD	5.396	0.86816	1,382,952	7,462,794
High	BAG36555.1	unnamed protein product [Homo sapiens]	16	57.9	CCT6A	5.373	0.86399	8,214,308	44,138,591
High	AAH40013.1	EARS2 protein [Homo sapiens]	1	59.9	EARS2	5.365	0.85922	517,536	2,930,871
High	AAF89953.1	vacuolar sorting protein 35 [Homo sapiens]	12	91.6	VPS35	5.328	0.85922	5,998,249	31,959,698
High	ACR16321.1	immunoglobulin light chain variable region, partial [Homo sapiens]	2	11.8		5.305	0.86075	1,552,037	8,233,736
High	NP_001677.2	ATP synthase subunit beta, mitochondrial precursor [Homo sapiens]	21	56.5	ATP5B	5.303	0.85713	14,921,488	79,132,133
High	BAF83220.1	unnamed protein product [Homo sapiens]	11	50.7	CSK	5.289	0.85676	5,265,668	27,852,503
High	BAA08205.1	proteasome activator hPA28 suunit beta [Homo sapiens]	5	27.3	PSME2	5.282	0.85341	925,915	4,890,280
High	BAD96210.1	chaperonin containing TCP1, subunit 8 (theta) variant, partial [Homo sapiens]	17	59.6	CCT8	5.261	0.85341	10,711,449	56,350,406
High	XP_011507965.1	PREDICTED: protoporphyrinogen oxidase isoform X1 [Homo sapiens]	1	60.6	PPOX	5.258	0.89085	114,904	604,688
High	NP_005902.1	S-adenosylmethionine synthase isoform type-2 [Homo sapiens]	10	43.6	MAT2A	5.257	0.85341	13,740,345	72,232,076
High	XP_011537005.1	PREDICTED: signal transducer and activator of transcription 6 isoform X1 [Homo sap	2	96.2	STAT6	5.218	0.85713	352,823	1,840,889
High	BAA06338.1	glycyl tRNA synthetase [Homo sapiens]	13	83.1	GARS	5.216	0.8504	7,490,646	39,071,882
High	AAB23031.1	protein disulphide isomerase, PDI {N-terminal} [human, Peptide Partial, 20 aa]	1	2.3		5.212	0.84998	2,725,044	14,203,286
High	NP_004930.1	ATP-dependent RNA helicase DDX1 [Homo sapiens]	14	82.4	DDX1	5.206	0.84898	6,787,272	35,337,610
High	XP_016867753.1	PREDICTED: calcium/calmodulin-dependent 3',5'-cyclic nucleotide phosphodiestera	2	90.4	PDE1C	5.204	0.87503	218,651	1,134,823
High	AAW67752.1	nucleophosmin [Homo sapiens]	3	32.8	NPM1	5.199	0.84787	3,232,896	16,807,554
High	BAG53567.1	unnamed protein product [Homo sapiens]	1	35.3	OTUB1	5.167	0.85341	408,307	2,109,551
High	XP_011514713.1	PREDICTED: trypsin-1 isoform X1 [Homo sapiens]	2	51.3	PRSS1	5.157	0.84318	976,653	5,036,373
High	AAA68889.1	angio-associated migratory cell protein [Homo sapiens]	1	49	AAMP	5.151	0.83612	615,291	3,169,606
High	BAF84383.1	unnamed protein product [Homo sapiens]	6	49.4	TMLHE	5.098	0.83462	2,928,623	14,928,882
High	Q58FF6.1	PUTATIVE PSEUDOGENE: RecName: Full=Putative heat shock protein HSP 90-beta 4	4	58.2	HSP90AB4	5.089	0.84382	1,377,223	7,009,268
High	CAC14047.1	aminopeptidase B, partial [Homo sapiens]	8	72.6	RNPEP	5.085	0.83352	3,485,335	17,724,159
High	DAA00404.1	TPA_exp: keratin 5c [Homo sapiens]	6	59.3	KRT74	5.069	0.83298	42,710,437	216,499,462
High	EAW82339.1	acyl-Coenzyme A oxidase 3, pristanoyl, isoform CRA_a [Homo sapiens]	6	81.2	ACOX3	5.067	0.83382	1,512,587	7,664,122
High	EAW55711.1	COP9 constitutive photomorphogenic homolog subunit 3 (Arabidopsis), isoform CR/	1	51.3	COPS3	5.06	0.8574	182,171	921,727
High	AAA20046.1	ubiquinol-cytochrome c reductase core I protein [Homo sapiens]	1	52.6	UQCRC1	5.056	0.83274	986,557	4,988,508
High	EAW56332.1	syntaxin binding protein 3, isoform CRA_c [Homo sapiens]	1	67.8	STXBP3	5.045	0.85523	308,528	1,556,481
High	NP_001035787.1	ubiquitin-conjugating enzyme E2 variant 3 isoform a [Homo sapiens]	4	52.2	UEVLD	5.042	0.83823	1,119,323	5,643,278
High	XP_011511125.1	PREDICTED: lipoma-preferred partner isoform X1 [Homo sapiens]	6	68.4	LPP	5.037	0.83171	1,939,799	9,770,327
High	EAW62679.1	heterogeneous nuclear ribonucleoprotein K, isoform CRA_e [Homo sapiens]	16	51.2	HNRNPK	5.033	0.83118	11,482,906	57,791,484
High	AAH09806.1	Methylenetetrahydrofolate dehydrogenase (NADP+ dependent) 1, methenyltetrahy	32	101.5	MTHFD1	5.018	0.82876	27,864,465	139,813,205
High	CAA82315.1	cytokeratin 9 [Homo sapiens]	34	62.1	KRT9	5.015	0.82876	138,195,104	693,115,543
High	XP_005274593.1	PREDICTED: E3 ubiquitin-protein ligase Midline-1 isoform X1 [Homo sapiens]	3	81.5	MID1	5.008	0.82572	649,342	3,251,767
High	NP_001308427.1	septin-10 isoform 4 [Homo sapiens]	4	62.9	SEPT10	5.004	0.8323	1,590,894	7,960,709
High	EAW50181.1	3'-phosphoadenosine 5'-phosphosulfate synthase 2, isoform CRA_a [Homo sapiens]	14	70.6	PAPSS2	4.988	0.82572	8,474,986	42,270,224
High	XP_011512974.1	PREDICTED: serpin B6 isoform X1 [Homo sapiens]	13	51.2	SERPINB6	4.986	0.82572	6,107,893	30,456,208
High	CAH18259.1	hypothetical protein [Homo sapiens]	8	73.6	KIAA1598;	4.983	0.82572	2,780,758	13,855,565
High	NP_997263.2	uncharacterized protein C15orf52 [Homo sapiens]	1	57.3	C15orf52;	4.947	0.82572	509,539	2,520,502
High	XP_016857913.1	PREDICTED: F-actin-capping protein subunit alpha-1 isoform X1 [Homo sapiens]	10	36.5	CAPZA1	4.945	0.82372	15,062,460	74,484,674
High	BAA23651.1	proteasome subunit p58 [Homo sapiens]	6	61	PSMD3	4.929	0.82164	2,631,064	12,967,445
High	XP_016855583.1	PREDICTED: nuclear migration protein nudC isoform X1 [Homo sapiens]	2	45.2	NUDC	4.922	0.8323	1,286,116	6,330,284
High	EAW79671.1	ATG3 autophagy related 3 homolog (S. cerevisiae), isoform CRA_d [Homo sapiens]	1	36.9	ATG3	4.906	0.84393	186,371	899,206
High	CAA28775.1	unnamed protein product [Homo sapiens]	27	57.1	P4HB	4.85	0.8139	30,922,037	149,959,929
High	NP_705618.1	nuclear pore complex protein Nup155 isoform 1 [Homo sapiens]	12	155.1	NUP155	4.826	0.81092	4,045,156	19,522,641
High	NP_035783.1	tubulin alpha-1A chain [Mus musculus]	28	50.1	Tuba1a	4.79	0.80674	7,740,001	37,072,730
High	XP_016862903.1	PREDICTED: sorting nexin-4 isoform X1 [Homo sapiens]	1	55.9	SNX4	4.79	0.83552	202,688	970,798
High	AAD17527.1	leucine aminopeptidase [Homo sapiens]	1	56	LAP3	4.784	0.83298	179,208	857,416
High	AAH70049.1	LanC lantibiotic synthetase component C-like 2 (bacterial) [Homo sapiens]	15	50.8	LANCL2	4.783	0.80563	21,490,654	102,786,369
High	AAB72237.1	ubiquitin protease [Homo sapiens]	1	108.5	USP4	4.773	0.81606	568,490	2,713,351
High	AAA51681.1	S-adenosylhomocysteine hydrolase [Homo sapiens]	13	47.7	AHCY	4.771	0.80477	12,979,597	61,931,013
High	NP_000628.2	glutathione reductase, mitochondrial isoform 1 precursor [Homo sapiens]	23	56.2	GSR	4.746	0.80285	84,285,964	400,058,261
High	XP_011540628.1	PREDICTED: dedicator of cytokinesis protein 7 isoform X1 [Homo sapiens]	18	242.4	DOCK7	4.743	0.80285	6,453,177	30,610,385
High	AAC15767.1	LIM protein [Homo sapiens]	13	64	PDLIM5	4.729	0.80285	9,757,995	46,143,797
High	NP_006357.1	adenylyl cyclase-associated protein 2 [Homo sapiens]	2	52.8	CAP2	4.725	0.81258	1,115,386	5,270,103
High	AAH28295.1	PEPD protein [Homo sapiens]	12	54.5	PEPD	4.7	0.80249	8,936,223	41,998,525
High	BAC87413.1	unnamed protein product [Homo sapiens]	1	118.3	FUK	4.692	0.82876	196,360	921,349
High	XP_007985196.1	PREDICTED: gephyrin isoform X8 [Chlorocebus sabaeus]	3	89.8	GPHN	4.682	0.80285	681,975	3,193,265
High	NP_001290203.1	ATP-citrate synthase isoform 3 [Homo sapiens]	34	126.1	ACLY	4.68	0.79876	30,774,051	144,027,505
High	AAQ13423.1	putative signal recognition particle [Homo sapiens]	2	74.6	SRP72	4.666	0.81258	577,041	2,713,636
High	BAH11911.1	unnamed protein product [Homo sapiens]	6	44.1	BCAT1	4.655	0.7956	3,666,341	17,067,169
High	XP_011517227.1	PREDICTED: niban-like protein 1 isoform X2 [Homo sapiens]	1	87.5	FAM129B	4.655	0.82287	193,112	899,287
High	BAG57019.1	unnamed protein product [Homo sapiens]	19	75.2		4.652	0.79558	19,755,593	91,908,504
High	EAX06811.1	oxysterol binding protein-like 9, isoform CRA_b [Homo sapiens]	2	86.2	OSBPL9	4.635	0.80285	994,549	4,609,823
High	EAW63188.1	farnesyltransferase, CAAX box, alpha, isoform CRA_a [Homo sapiens]	4	52.6	FNTA	4.631	0.81092	345,038	1,597,707
High	EAX06804.1	nardilysin (N-arginine dibasic convertase), isoform CRA_a [Homo sapiens]	7	139.5	NRD1; NR	4.625	0.79167	2,776,541	12,840,194
High	BAD92951.1	crystallin, zeta variant, partial [Homo sapiens]	1	35.4	CRYZ	4.623	0.82164	260,611	1,204,688
High	AAH71727.1	Eukaryotic translation elongation factor 1 alpha 1 [Homo sapiens]	26	50.2	EEF1A1	4.596	0.79033	603,215,551	2,772,405,994
High	XP_006721823.1	PREDICTED: tripartite motif-containing protein 65 isoform X1 [Homo sapiens]	5	58.6	TRIM65	4.596	0.79033	2,209,930	10,157,626
High	EAW61507.1	arginyl-tRNA synthetase [Homo sapiens]	20	75.3	RARS	4.581	0.79033	13,880,636	63,590,492
High	BAG59669.1	unnamed protein product [Homo sapiens]	3	51.1	GDI2	4.573	0.80285	588,435	2,685,603
High	AAH77077.1	DPYSL3 protein [Homo sapiens]	13	73.9	DPYSL3	4.552	0.78967	4,096,754	18,649,163
High	AAH62302.1	Phosphoglycerate mutase 1 (brain) [Homo sapiens]	1	28.8	PGAM1	4.551	0.82372	127,831	557,026
High	AAK73017.1	chronic myelogenous leukemia tumor antigen 66 [Homo sapiens]	1	66.7	NUDCD1	4.547	0.80285	392,522	1,773,868
High	BAG59420.1	unnamed protein product [Homo sapiens]	2	65.6	HDAC2	4.534	0.79553	556,627	2,523,680
High	AAA35656.1	calelectrin [Homo sapiens]	3	75.9	ANXA6	4.528	0.79161	1,074,132	4,863,552

High	BAB71628.1	unnamed protein product [Homo sapiens]	1	36.7	UBLCP1	4.519	0.79729	472,797	2,136,511
High	NP_005711.1	actin-related protein 2/3 complex subunit 1B [Homo sapiens]	1	40.9	ARPC1B	4.501	0.79558	595,040	2,678,420
High	XP_005269689.1	PREDICTED: cytosolic purine 5'-nucleotidase isoform X1 [Homo sapiens]	2	65.8	NT5C2	4.496	0.79033	701,471	3,153,727
High	AAB05994.1	putative transmembrane protein precursor [Homo sapiens]	1	80.4	STT3A	4.492	0.79558	1,003,323	4,506,704
High	AAR91619.1	hornerin precursor [Homo sapiens]	2	282.2	HRNR	4.488	0.79033	788,227	3,537,463
High	AAH05404.1	SEC23B protein [Homo sapiens]	14	86.4	SEC23B	4.465	0.77798	5,297,941	23,656,869
High	NP_004716.1	mitotic checkpoint protein BUB3 isoform a [Homo sapiens]	7	37.1	BUB3	4.463	0.77798	3,982,106	17,773,633
High	NP_001312.1	cysteine and glycine-rich protein 2 [Homo sapiens]	2	20.9	CSRP2	4.439	0.79161	601,332	2,669,069
High	AAC32232.1	RIP protein kinase [Homo sapiens]	2	75.9	RIPK1	4.437	0.78856	756,288	3,355,306
High	EAW49686.1	ARP1 actin-related protein 1 homolog A, centractin alpha (yeast), isoform CRA_b [H	2	42.6	ACTR1A	4.419	0.79161	1,169,775	5,169,018
High	BAG60258.1	unnamed protein product [Homo sapiens]	3	39.8	STRAP	4.394	0.78906	1,418,118	6,230,665
High	NP_060238.3	leucine-rich repeat-containing protein 40 [Homo sapiens]	1	68.2	LRRC40	4.393	0.79161	439,298	1,929,794
High	AAH09408.1	CTP synthase [Homo sapiens]	9	66.7	CTPS1	4.383	0.77112	4,997,190	21,903,496
High	NP_068800.2	phosphoglucomutase-like protein 5 [Homo sapiens]	11	62.2	PGM5	4.382	0.77112	4,107,768	18,000,304
High	BAA23987.1	ATP-dependent RNA helicase #46 [Homo sapiens]	14	92.8	DHX15	4.381	0.77112	5,955,837	26,095,361
High	AAB59391.1	hypoxanthine phosphoribosyltransferase, partial [Homo sapiens]	5	24.5	HPRT1	4.381	0.77112	4,444,070	19,468,317
High	AAH23990.1	Annexin A2 [Homo sapiens]	29	38.6	ANXA2	4.376	0.77055	226,985,370	993,259,265
High	XP_011539331.1	PREDICTED: WD repeat-containing protein 47 isoform X1 [Homo sapiens]	1	103.1	WDR47	4.358	0.80477	171,104	745,091
High	BAD96889.1	phosphoribosyl pyrophosphate synthetase 1 variant, partial [Homo sapiens]	4	34.8	PRPS1	4.35	0.76728	2,265,193	9,853,170
High	BAD92974.1	methylcrotonoyl-Coenzyme A carboxylase 1 (alpha) variant, partial [Homo sapiens]	1	82.3	MCCC1	4.343	0.78302	497,869	2,166,722
High	NP_056365.1	oxysterol-binding protein-related protein 3 isoform a [Homo sapiens]	5	101.2	OSBPL3	4.316	0.765	1,885,859	8,138,477
High		T46292 hypothetical protein DKFZp434E0610.1 - human (fragment)	3	65.8		4.308	0.77182	807,957	3,480,974
High	NP_001489.1	glutamate--cysteine ligase catalytic subunit isoform a [Homo sapiens]	2	72.7	GCLC	4.262	0.78302	491,074	2,092,735
High	NP_002818.1	tyrosine-protein phosphatase non-receptor type 1 isoform 1 [Homo sapiens]	9	49.9	PTPN1	4.252	0.75562	2,389,615	10,160,395
High	NP_001135888.1	aminoacyl tRNA synthase complex-interacting multifunctional protein 1 isoform b p	2	37	AIMP1	4.247	0.77127	861,546	3,658,865
High	NP_001264890.1	stromal interaction molecule 1 isoform 1 precursor [Homo sapiens]	1	88.6	STIM1	4.241	0.79849	181,696	778,306
High	BAD96709.1	capping protein (actin filament) muscle Z-line, alpha 2 variant, partial [Homo sapien	8	32.9	CAPZA2	4.238	0.75401	12,351,418	52,346,506
High	EAW63580.1	protein phosphatase 2 (formerly 2A), regulatory subunit B (PR 52), alpha isoform, is	11	55.1	PPP2R2A	4.237	0.7539	8,808,985	37,321,350
High	NP_004757.1	coatomer subunit beta' [Homo sapiens]	11	102.4	COPB2	4.236	0.7539	4,254,610	18,021,963
High	AAC39757.1	spectrin SH3 domain binding protein 1 [Homo sapiens]	5	55.1	ABI1	4.224	0.74822	2,049,297	8,655,782
High	CAA80852.1	mitogen inducible gene mig-2, partial [Homo sapiens]	3	81.9	FERMT2	4.209	0.76641	683,395	2,876,346
High	CAA37064.1	t-complex polypeptide 1 [Homo sapiens]	20	60.4	TCP1	4.177	0.74454	12,575,026	52,526,677
High	BAA09534.1	P1cdc47 [Homo sapiens]	2	81.2	MCM7	4.156	0.78964	324,586	1,348,898
High	EAW74770.1	cortactin, isoform CRA_d [Homo sapiens]	26	62.2	CTTN	4.135	0.74135	40,369,181	166,937,077
High	AAB59537.1	complement component C4A, partial [Homo sapiens]	2	193.5	C4A	4.128	0.7408	2,064,162	8,519,831
High	AAH12131.1	Chromosome 14 open reading frame 149 [Homo sapiens]	1	38.1	L3HYPDH	4.124	0.75784	535,713	2,209,060
High	NP_852664.1	phosphatidylinositol 3-kinase regulatory subunit alpha isoform 1 [Homo sapiens]	2	83.5	PIK3R1	4.109	0.77182	352,146	1,481,271
High	NP_006355.2	protein transport protein Sec23A [Homo sapiens]	25	86.1	SEC23A	4.091	0.73791	19,208,826	78,575,950
High	BAG63306.1	unnamed protein product [Homo sapiens]	6	62.9	PGM3	4.085	0.73728	3,000,548	12,258,277
High	XP_011522506.1	PREDICTED: septin-9 isoform X1 [Homo sapiens]	22	68.6	SEPT9	4.072	0.73523	29,198,248	118,886,133
High	AAH13630.1	JTV1 gene [Homo sapiens]	1	35.3	AIMP2	4.06	0.77112	246,592	999,690
High	XP_005273547.1	PREDICTED: inhibitor of nuclear factor kappa-B kinase subunit beta isoform X1 [Hor	5	91.8	IKBKB	4.058	0.73475	3,899,948	15,825,123
High	XP_011523129.2	PREDICTED: MAGUK p55 subfamily member 2 isoform X1 [Homo sapiens]	3	70.6	MPP2	4.047	0.74822	1,207,554	4,887,258
High	AAF04523.1	F-box protein Fbx22, partial [Homo sapiens]	3	8.8	FBXO22	4.044	0.77645	283,016	1,203,882
High	EAW84659.1	phosphoinositide-3-kinase, regulatory subunit 2 (p85 beta), isoform CRA_a [Homo s	1	81.5	PIK3R2	4.041	0.77219	237,415	958,502
High	NP_056937.2	UBX domain-containing protein 1 isoform 1 [Homo sapiens]	2	35.1	UBXN1	4.004	0.75784	505,885	2,025,673
High	NP_060289.2	mitochondrial ribonuclease P protein 1 precursor [Homo sapiens]	2	47.3	TRMT10C	3.986	0.7722	344,420	1,372,700
High	BAG65428.1	unnamed protein product [Homo sapiens]	29	63.9	HSPA1A	3.983	0.72436	2,882,463	11,479,642
High	NP_055112.2	persulfide dioxygenase ETHE1, mitochondrial isoform 1 [Homo sapiens]	4	27.9	ETHE1	3.972	0.72388	1,815,866	7,212,067
High	NP_005498.1	cofilin-1 [Homo sapiens]	4	18.5	CFL1	3.966	0.72068	2,950,787	11,703,719
High	XP_011538682.1	PREDICTED: protein transport protein Sec24C isoform X1 [Homo sapiens]	22	120.9	SEC24C	3.961	0.72053	18,433,612	73,014,621
High	EAW82197.1	scribbled homolog (Drosophila), isoform CRA_b [Homo sapiens]	5	177.8	SCRIB	3.96	0.7539	500,541	2,007,239
High	NP_064710.4	UPF0505 protein C16orf62 isoform 1 [Homo sapiens]	1	118.5	C16orf62	3.95	0.77182	92,363	359,378
High	XP_016859290.1	PREDICTED: glutamine--fructose-6-phosphate aminotransferase [isomerizing] 1 isof	18	81.5	GFPT1	3.932	0.71604	9,888,659	38,883,878
High	EAX10092.1	phosphorylase, glycogen; brain, isoform CRA_b [Homo sapiens]	12	98.8	PYGB	3.89	0.70913	3,468,802	13,492,114
High	NP_000010.1	acetyl-CoA acetyltransferase, mitochondrial precursor [Homo sapiens]	9	45.2	ACAT1	3.879	0.70774	6,031,393	23,394,973
High	XP_005251037.1	PREDICTED: plectin isoform X5 [Homo sapiens]	250	516.6	PLEC	3.866	0.73475	1,582,997	6,119,567
High	NP_005023.2	plastin-3 isoform 1 [Homo sapiens]	37	70.8	PLS3	3.863	0.70494	86,805,098	335,368,537
High	AAC25560.1	citrate synthase [Homo sapiens]	4	51.7	CS	3.862	0.70311	2,159,346	8,339,375
High	BAA07267.1	hRlf beta subunit (p102 protein) [Homo sapiens]	15	90.9	MCM3	3.852	0.70311	9,707,400	37,389,896
High	AAH93030.1	Methionine adenosyltransferase II, beta [Homo sapiens]	4	37.5	MAT2B	3.85	0.70311	5,566,708	21,429,803
High	BAG62290.1	unnamed protein product [Homo sapiens]	6	33.2	MTAP	3.85	0.71182	2,025,410	7,796,920
High	NP_001303303.1	malate dehydrogenase, peroxisomal isoform MDH1x [Homo sapiens]	3	38.6	MDH1	3.803	0.732	566,897	2,156,046
High	AAH12604.1	RTCD1 protein [Homo sapiens]	1	40.7	RTCA	3.757	0.74135	366,708	1,377,725
High	XP_005264900.1	PREDICTED: oxysterol-binding protein-related protein 10 isoform X1 [Homo sapiens	1	84.3	OSBPL10	3.756	0.73329	505,367	1,897,967
High	NP_036220.1	6-phosphogluconolactonase [Homo sapiens]	1	27.5	PGLS	3.752	0.72991	545,661	2,047,387
High	AAO13879.1	septin SEPT8_v2, partial [Homo sapiens]	4	63.9	SEPT8	3.749	0.74135	251,925	946,308
High	NP_000128.1	fumarylacetoacetase [Homo sapiens]	3	46.3	FAH	3.747	0.72849	1,041,210	3,901,175
High	BAF83082.1	unnamed protein product [Homo sapiens]	15	59.7	CCT5	3.734	0.68943	7,457,011	27,847,516
High	EAW77664.1	sorting nexin 1, isoform CRA_b, partial [Homo sapiens]	14	59.7	SNX1	3.726	0.68943	8,997,219	33,521,226
High	CAG33614.1	REC14 [Homo sapiens]	2	33.6	WDR61	3.724	0.74167	333,991	1,243,668
High	CAD89917.1	hypothetical protein [Homo sapiens]	1	109.1	USO1	3.718	0.72053	660,247	2,406,505
High	EAW60681.1	keratin 10 (epidermolytic hyperkeratosis; keratosis palmaris et plantaris), isoform Cl	35	63.3	KRT10	3.712	0.68943	450,478,984	1,672,345,003
High	NP_001245367.1	threonine--tRNA ligase, cytoplasmic isoform 2 [Homo sapiens]	3	86.8	TARS	3.687	0.74167	298,286	1,099,855
High	BAB14194.1	unnamed protein product [Homo sapiens]	7	35.6	CPPED1	3.599	0.67147	4,732,538	17,031,431
High	XP_006710589.1	PREDICTED: microtubule-actin cross-linking factor 1 isoform X15 [Homo sapiens]	3	860.9	MACF1	3.573	0.69725	812,773	2,905,460
High	BAF82741.1	unnamed protein product [Homo sapiens]	1	169.6	ARAP3	3.57	0.72991	314,813	1,331,843
High	NP_001304310.1	heat shock 70 kDa protein 4L isoform 2 [Homo sapiens]	3	97.6	HSPA4L	3.564	0.69031	610,298	2,174,899
High	EAX11205.1	dynein, cytoplasmic 1, intermediate chain 2, isoform CRA_g [Homo sapiens]	4	71.5	DYNC1I2	3.557	0.68943	1,413,494	5,027,742
High	NP_954592.1	histone-arginine methyltransferase CARM1 [Homo sapiens]	11	65.8	CARM1	3.511	0.65122	8,886,843	31,205,281
High	NP_071440.1	ras-related GTP-binding protein C isoform 1 [Homo sapiens]	6	44.2	RRAGC	3.509	0.6465	2,537,966	8,905,718
High	AAA36534.1	prolyl 4-hydroxylase alpha subunit (EC 1.14.11.2) [Homo sapiens]	4	60.8	P4HA1	3.462	0.68852	1,735,058	6,006,532
High	NP_068810.3	transcription factor p65 isoform 1 [Homo sapiens]	4	60.2	RELA	3.438	0.6476	2,103,745	7,232,617
High	AAC51518.1	ER-60 protein [Homo sapiens]	24	56.7	PDIA3	3.42	0.63622	43,955,584	150,315,743
High	AAL30772.1	endofin [Homo sapiens]	1	168.7	ZFYVE16	3.402	0.70311	344,677	1,172,492
High	CAH18683.1	hypothetical protein [Homo sapiens]	8	47.9	PAICS	3.381	0.62649	3,409,737	11,529,387
High	BAG58182.1	unnamed protein product [Homo sapiens]	2	48.8	SCRN1	3.374	0.69562	231,127	779,866
High	AAB50217.1	protein disulfide isomerase-related protein 5, partial [Homo sapiens]	20	46.2	PDIA6	3.346	0.62138	111,905,045	374,445,487
High	XP_003823383.1	PREDICTED: eukaryotic translation initiation factor 3 subunit H [Pan paniscus]	1	41.6	EIF3H	3.311	0.66778	801,377	2,653,352
High	AAH68561.1	Solute carrier family 25 (mitochondrial carrier; phosphate carrier), member 24 [Hor	1	53.3	SLC25A24	3.287	0.69084	104,792	327,865
High	NP_002757.2	phosphoribosyl pyrophosphate synthase-associated protein 1 isoform 1 [Homo sap	3	42.4	PRPSAP1	3.28	0.66189	830,661	2,724,623
High	BAG35456.1	unnamed protein product [Homo sapiens]	3	54.2	STK38	3.262	0.66005	1,871,598	6,105,278
High	BAD92170.1	ribosomal protein S6 kinase, 90kDa, polypeptide 3 variant, partial [Homo sapiens]	1	89.3	RPS6KA3	3.255	0.68943	97,231	325,980
High	EAW65686.1	phosphorylase, glycogen; liver (Hers disease, glycogen storage disease type VI), isofo	5	97.5	PYGL	3.25	0.69562	143,080	465,078
High	NP_060037.3	N-acetyl-D-glucosamine kinase isoform 1 [Homo sapiens]	1	42	NAGK	3.235	0.64765	649,113	2,115,126

High	AAH48980.1	Protein kinase, AMP-activated, alpha 1 catalytic subunit [Homo sapiens]	1	65.5	PRKAA1	3.23	0.68943	418,809	1,352,938
High	BAA91754.1	unnamed protein product [Homo sapiens]	1	84.3	DHX32	3.221	0.69031	94,080	305,179
High	AAH07979.1	Serine hydroxymethyltransferase 1 (soluble) [Homo sapiens]	5	53.1	SHMT1	3.215	0.64763	1,661,872	5,342,171
High	NP_068817.1	protein transport protein Sec24A isoform 1 [Homo sapiens]	4	119.7	SEC24A	3.213	0.6489	1,049,347	3,371,829
High	NP_005892.1	mothers against decapentaplegic homolog 2 isoform 1 [Homo sapiens]	2	52.3	SMAD2	3.204	0.64021	1,356,499	4,346,285
High	AAH02690.1	Keratin 14 [Homo sapiens]	30	51.6	KRT14	3.201	0.5985	18,577,238	59,469,716
High	BAA28219.1	prostacyclin synthase, partial [Homo sapiens]	1	57.8	PTGIS	3.161	0.68901	134,170	422,802
High	NP_071415.1	methylcrotonoyl-CoA carboxylase beta chain, mitochondrial [Homo sapiens]	7	61.3	MCCC2	3.158	0.60251	2,366,366	7,472,596
High	NP_003312.3	elongation factor Tu, mitochondrial precursor [Homo sapiens]	15	49.8	TUFM	3.147	0.58406	18,965,789	59,680,761
High	NP_001075.1	procollagen-lysine,2-oxoglutarate 5-dioxygenase 3 precursor [Homo sapiens]	8	84.7	PLOD3	3.109	0.57551	2,861,899	8,896,999
High	NP_003290.1	endoplasmin precursor [Homo sapiens]	13	92.4	HSP90B1	3.076	0.56594	8,285,944	25,489,906
High	NP_061740.2	protocadherin gamma-A4 isoform 1 precursor [Homo sapiens]	1	103.9	PCDHGA4	3.076	0.61942	700,023	2,153,089
High	NP_005800.3	peroxiredoxin-2 [Homo sapiens]	6	21.9	PRDX2	3.067	0.564	3,192,059	9,791,174
High	NP_001269374.1	EH domain-containing protein 1 isoform 2 [Homo sapiens]	6	61.9	EHD1	3.05	0.61445	1,982,230	6,045,053
High	AAA60261.1	DNA helicase [Homo sapiens]	9	74.8	RECQL	3.037	0.55938	5,202,271	15,800,482
High	NP_817124.1	tubulin beta-8 chain [Homo sapiens]	13	49.7	TUBB8	2.959	0.54173	3,973,773	11,756,414
High	BAG50851.1	unnamed protein product [Homo sapiens]	1	42.9	ILKAP	2.956	0.61911	531,321	1,570,803
High	XP_005246274.1	PREDICTED: abl interactor 2 isoform X1 [Homo sapiens]	3	58.7	ABI2	2.927	0.62138	275,682	806,938
High	XP_006716248.1	PREDICTED: protein disulfide-isomerase A4 isoform X1 [Homo sapiens]	8	73	PDIA4	2.924	0.53439	4,551,691	13,311,391
High	AAH99905.1	Mitogen-activated protein kinase 1 [Homo sapiens]	8	41.3	MAPK1	2.904	0.52927	6,263,319	18,187,649
High	EAX05906.1	SEC31-like 1 (S. cerevisiae), isoform CRA_a [Homo sapiens]	2	134.9	SEC31A	2.903	0.60583	811,099	2,354,899
High	AAN85571.1	class II beta tubulin isotype [Homo sapiens]	29	49.9	TUBB2A	2.9	0.60583	547,435	1,587,334
High	AAF17198.1	heat shock protein hsp70-related protein [Homo sapiens]	1	54.7	HSPA14	2.892	0.62755	361,434	1,044,304
High	CAA40748.1	alpha 1(VIII) collagen [Homo sapiens]	6	73.4	COL8A1	2.891	0.5284	10,512,679	30,395,674
High	CAA84279.1	Dsc1b precursor [Homo sapiens]	3	100	DSC1	2.861	0.55907	2,102,821	6,015,785
High	NP_036252.1	CD2-associated protein [Homo sapiens]	11	71.4	CD2AP	2.793	0.50263	10,469,121	29,244,526
High	BAD97348.1	Protein KIAA0196 variant, partial [Homo sapiens]	3	134.8	KIAA0196;	2.77	0.6079	459,812	1,273,708
High	EAW63034.1	thioredoxin-like 1, isoform CRA_a [Homo sapiens]	5	36.7	TXNL1	2.68	0.47903	2,719,695	7,288,956
High	NP_006358.1	adenylyl cyclase-associated protein 1 [Homo sapiens]	9	51.6	CAP1	2.672	0.46246	2,966,065	7,926,128
High	ABB90543.1	TRIM5alpha [Homo sapiens]	4	56.3	TRIM5	2.659	0.54475	764,956	2,034,124
High	AAH24292.1	Keratin 5 [Homo sapiens]	37	62.3	KRT5	2.633	0.46142	42,210,280	111,133,938
High	BAG37782.1	unnamed protein product [Homo sapiens]	1	24.6	PSMD9	2.632	0.4613	4,404,876	11,593,350
High	BAD20938.1	2'-phosphodiesterase [Homo sapiens]	3	67.3	PDE12	2.616	0.53145	1,567,678	4,101,585
High	AAA79948.1	fus-like protein, partial [Homo sapiens]	5	53.3	FUS	2.58	0.45672	2,484,629	6,409,336
High	AAS94255.1	PIG48 [Homo sapiens]	12	60.5	CCT3	2.571	0.44554	26,352,547	67,755,333
High	AAB38416.1	mitochondrial aconitase [Homo sapiens]	2	85.6	ACO2	2.571	0.53529	870,403	2,237,636
High	AAF04034.1	mutant cytokeratin 14, partial [Homo sapiens]	4	8.6	KRT14	2.569	0.53529	1,141,206	2,931,529
Medium	NP_001275897.1	acyl-CoA synthetase family member 2, mitochondrial isoform 1 [Homo sapiens]	3	70.6	ACSF2	2.552	0.53867	552,048	1,408,798
Medium	AFA52005.1	keratin 1 [Homo sapiens]	45	66.1	KRT1	2.544	0.43896	1,180,220,188	3,002,856,720
Medium	EAW48851.1	aldehyde dehydrogenase 7 family, member A1, partial [Homo sapiens]	7	59	ALDH7A1	2.539	0.43876	3,325,933	8,445,168
Medium	AAF01333.1	serum albumin precursor [Homo sapiens]	17	69.2	ALB	2.536	0.43789	8,388,075	21,271,126
Medium	EAW79936.1	aldolase A, fructose-bisphosphate, isoform CRA_b [Homo sapiens]	13	39.8	ALDOA	2.514	0.43347	29,056,242	73,037,546
Medium	ACF34430.1	immunoglobulin light chain, partial [Homo sapiens]	2	24.3		2.456	0.52828	235,993	579,584
Medium	NP_002565.1	peroxiredoxin-1 [Homo sapiens]	13	22.1	PRDX1	2.412	0.41021	30,618,405	73,850,581
Medium	DAA00377.1	TPA_exp: keratin 5b [Homo sapiens]	7	56.9	KRT78	2.398	0.45672	1,896,325	4,548,235
Medium	NP_001287783.1	dermcidin isoform 2 preproprotein [Homo sapiens]	1	12.4	DCD	2.38	0.50176	701,016	1,668,705
Medium	AIU95779.1	immunoglobulin kappa light chain variable region, partial [Homo sapiens]	1	10.7		2.373	0.50628	485,915	732,925
Medium	CAE45922.1	hypothetical protein [Homo sapiens]	4	61.6	ARCN1	2.321	0.48806	1,607,519	3,730,344
Medium	BAD96798.1	lactate dehydrogenase A variant, partial [Homo sapiens]	13	36.7	LDHA	2.304	0.38007	13,642,662	31,430,719
Medium	BAB55264.1	unnamed protein product [Homo sapiens]	1	90.5	LEPRE1; P	2.301	0.48993	238,075	548,801
Medium	AAK29181.1	catalase [Homo sapiens]	7	59.7	CAT	2.297	0.37839	4,073,570	9,356,446
Medium	AAC18044.1	antigen NY-CO-25, partial [Homo sapiens]	9	98.4	HSPH1	2.273	0.37095	10,423,740	23,698,182
Medium	AAB61638.1	beta-3A-adaptin subunit of the AP-3 complex [Homo sapiens]	4	121.3	AP3B1	2.225	0.43643	1,795,218	3,993,545
Medium	AAH08435.1	Peroxiredoxin 3 [Homo sapiens]	6	27.7	PRDX3	2.186	0.34903	15,504,230	33,891,343
Medium	AAA60231.1	glycogen phosphorylase [Homo sapiens]	7	97.1	PYGM	2.176	0.43923	1,449,383	3,154,413
Medium	NP_003906.2	copine-1 isoform b [Homo sapiens]	1	59.7	CPNE1	2.173	0.44991	392,854	853,626
Medium	NP_004452.1	phenylalanine--tRNA ligase alpha subunit [Homo sapiens]	10	57.5	FARSA	2.156	0.34028	7,605,209	16,394,387
Medium	AAC41939.1	cytoplasmic antiproteinase 2 [Homo sapiens]	9	42.8	SERPINB8	2.154	0.33504	3,619,781	7,797,846
Medium	BAF84993.1	unnamed protein product [Homo sapiens]	25	59.3	G6PD	2.116	0.3307	43,646,039	92,367,683
Medium	CAD97647.1	hypothetical protein, partial [Homo sapiens]	4	125	RANBP6	2.083	0.44694	88,455	199,680
Medium	BAG61476.1	unnamed protein product [Homo sapiens]	3	83.7		2.035	0.41072	1,212,179	2,466,439
Medium	AAG47842.1	glutaminase [Homo sapiens]	8	73.4	GLS	2.022	0.30548	5,325,206	10,768,862
Medium	NP_004896.1	peroxiredoxin-6 [Homo sapiens]	7	25	PRDX6	2.019	0.30472	6,528,795	13,178,719
Medium	BAG35356.1	unnamed protein product [Homo sapiens]	14	79.6	HSD17B4	1.986	0.29507	10,248,480	20,349,619
Medium	NP_005712.1	actin-related protein 3 isoform 1 [Homo sapiens]	11	47.3	ACTR3	1.957	0.28755	7,613,879	14,901,210
Medium	AAH07909.1	FASN protein [Homo sapiens]	20	48.3	FASN	1.935	0.3807	1,025,093	1,983,733
Medium	NP_001304995.1	protein transport protein Sec24D isoform 2 [Homo sapiens]	2	113	SEC24D	1.926	0.39574	409,072	803,122
Medium	3L4G_B	Chain B, Crystal Structure Of Homo Sapiens Cytoplasmic Phenylalanyl-T Synthetase	14	66.1		1.892	0.27053	24,087,647	45,584,092
Medium	AAH11567.1	Tripartite motif-containing 7 [Homo sapiens]	1	23.7	TRIM7	1.855	0.36415	936,725	1,737,997
Medium	XP_006712994.1	PREDICTED: ubiquitin-like modifier-activating enzyme ATG7 isoform X1 [Homo sapie	12	81.7	ATG7	1.848	0.25782	8,640,714	15,972,245
Medium	NP_005262.1	glutamate dehydrogenase 1, mitochondrial isoform a precursor [Homo sapiens]	15	61.4	GLUD1	1.83	0.25296	5,650,443	10,340,558
Medium	NP_005737.1	nicotinamide phosphoribosyltransferase precursor [Homo sapiens]	1	55.5	NAMPT	1.805	0.36061	394,389	728,432
Medium	EAW92982.1	ubiquitin carboxyl-terminal esterase L1 (ubiquitin thiolesterase), isoform CRA_d, pa	1	42.7	UCHL1	1.795	0.38463	824,008	1,479,474
Medium	NP_659489.1	uracil phosphoribosyltransferase homolog isoform 1 [Homo sapiens]	1	33.8	UPRT	1.771	0.27431	2,971,191	5,262,971
Medium	AAF75261.1	N-acetylneuraminic acid phosphate synthase [Homo sapiens]	1	40.3	NANS	1.743	0.35855	606,996	1,067,271
Medium	CAA25833.1	glyceraldehyde-3-phosphate dehydrogenase [Homo sapiens]	20	36	GAPDH	1.742	0.22931	31,111,931	54,205,074
Medium	AAH07104.1	Peptidylprolyl isomerase A (cyclophilin A) [Homo sapiens]	3	18	PPIA	1.735	0.27796	2,370,861	4,114,369
Medium	CAA39976.1	desmoglein type 1 [Homo sapiens]	5	113.6	DSG1	1.734	0.32258	1,095,829	1,900,074
Medium	NP_000414.2	keratin, type II cytoskeletal 2 epidermal [Homo sapiens]	49	65.4	KRT2	1.721	0.22355	9,232,444	15,890,832
Medium	AAA80581.1	succinate dehydrogenase iron-protein subunit B [Homo sapiens]	5	31.8	SDHB	1.712	0.30548	2,145,279	3,672,612
Medium	NP_443730.1	guanine nucleotide-binding protein subunit beta-like protein 1 [Homo sapiens]	2	35.6	GNB1L	1.664	0.32418	233,822	389,023
Medium	NP_001302466.1	L-lactate dehydrogenase B chain isoform LDHBx [Homo sapiens]	10	37.4	LDHB	1.633	0.20328	9,952,063	16,255,266
Medium	XP_005266835.2	PREDICTED: serpin B12 isoform X1 [Homo sapiens]	4	49.8	SERPINB1	1.592	0.27359	2,207,425	3,514,886
Medium	AAG36781.1	inorganic pyrophosphatase 2 [Homo sapiens]	4	37.9	PPA2	1.585	0.19011	6,333,870	10,036,675
Medium	NP_065908.1	vacuolar protein sorting-associated protein 18 homolog [Homo sapiens]	3	110.1	VPS18	1.583	0.29407	1,644,412	2,603,404
Medium	XP_011528297.1	PREDICTED: RNA-binding protein EWS isoform X1 [Homo sapiens]	5	70	EWSR1	1.529	0.17728	7,308,095	11,173,577
Medium	NP_000687.3	4-trimethylaminobutyraldehyde dehydrogenase [Homo sapiens]	9	56.3	ALDH9A1	1.525	0.17728	5,959,004	9,089,289
Medium	BAD96912.1	enolase 1 variant, partial [Homo sapiens]	24	47.2	ENO1	1.463	0.16289	89,488,699	130,916,720
Medium	CCN27103.1	MHC class I antigen, partial [Homo sapiens]	11	31.6	HLA-A	1.463	0.17104	4,024,504	5,886,218
Medium	EAW97717.1	5'-nucleotidase domain containing 3, isoform CRA_c [Homo sapiens]	1	63.4	NT5DC3	1.395	0.24767	301,775	463,685
Medium	BAG60162.1	unnamed protein product [Homo sapiens]	9	47.4	C22orf28;	1.391	0.25376	940,450	1,321,443
Medium	XP_006720633.1	PREDICTED: pyruvate kinase PKM isoform X1 [Homo sapiens]	34	65.8	PKM	1.37	0.14169	111,941,344	153,376,907
Medium	NP_001014364.1	filaggrin-2 [Homo sapiens]	1	247.9	FLG2	1.355	0.24088	397,947	539,150
Medium	AAA99353.1	immunoglobulin kappa chain, partial [Homo sapiens]	1	10.9		1.307	0.21203	1,192,947	1,559,079
Medium	NP_003680.2	aflatoxin B1 aldehyde reductase member 2 isoform 1 [Homo sapiens]	1	39.6	AKR7A2	1.304	0.213	339,036	440,890

Medium	NP_001254479.2	titin isoform IC [Homo sapiens]	1	3992.2	TTN	1.297	0.21267	299,751	388,900
Medium	EAW96627.1	keratin 6B, isoform CRA_b [Homo sapiens]	35	60	KRT6B	1.279	0.14185	3,683,581	4,720,461
Medium	XP_011524051.1	PREDICTED: mRNA cap guanine-N7 methyltransferase isoform X1 [Homo sapiens]	2	58.5	RNMT	1.262	0.20492	1,190,543	1,502,390
Medium	NP_060692.2	alpha-parvin [Homo sapiens]	1	46.6	PARVA	1.248	0.20599	243,122	423,458
Medium	NP_000413.1	keratin, type I cytoskeletal 17 [Homo sapiens]	20	48.1	KRT17	1.182	0.18388	2,089,689	2,470,442
Medium	AAH17210.1	Coproporphyrinogen oxidase [Homo sapiens]	3	50.1	CPOX	1.177	0.18195	2,388,518	2,810,295
Medium	BAD97334.1	transketolase variant, partial [Homo sapiens]	33	67.9	TKT	1.155	0.09474	198,064,473	228,673,409
Medium	NP_001311045.1	UDP-N-acetylhexosamine pyrophosphorylase isoform b [Homo sapiens]	1	58.7	UAP1	1.114	0.17027	133,673	155,117
Medium	NP_001276718.1	glucose-6-phosphate isomerase isoform 3 [Homo sapiens]	11	67.2	GPI	1.075	0.07941	8,338,181	8,959,828
Medium	XP_005258776.1	PREDICTED: protein LSM14 homolog A isoform X1 [Homo sapiens]	1	53.4	LSM14A	1.069	0.14837	954,163	1,027,058
Medium	AAC03787.1	malate dehydrogenase precursor [Homo sapiens]	3	35.5	MDH2	1.051	0.15367	1,633,628	1,716,241
Medium	XP_011527784.1	PREDICTED: dual specificity tyrosine-phosphorylation-regulated kinase 1A isoform X	1	86.2	DYRK1A	1.049	0.14511	2,636,364	2,766,295
Medium	NP_004159.2	succinate dehydrogenase [ubiquinone] flavoprotein subunit, mitochondrial isoform	29	72.6	SDHA	1.015	0.06843	163,684,098	166,126,389
Medium	AAH12509.1	EEF1A1 protein, partial [Homo sapiens]	4	17	EEF1A1	0.998	0.13646	2,306,473	2,301,598
Medium	ACH48232.1	uridine monophosphate synthetase isoform I [Homo sapiens]	3	52.2	UMPS	0.993	0.09697	3,239,107	3,215,785
Medium	AAB35421.1	type I keratin 16 [Homo sapiens]	28	51.2	KRT16	0.93	0.05405	13,999,437	13,024,391
Medium	NP_002622.2	6-phosphogluconate dehydrogenase, decarboxylating isoform 1 [Homo sapiens]	2	53.1	PGD	0.9	0.1107	1,276,229	1,159,637
Medium	AAC41769.1	keratin type II [Homo sapiens]	34	60.2	KRT6A	0.879	0.04579	11,574,563	10,175,357
Medium	BAD97123.1	bleomycin hydrolase variant, partial [Homo sapiens]	1	52.6	BLMH	0.863	0.12329	924,575	805,249
Medium	AAI17415.1	DIAPH2 protein [Homo sapiens]	1	125.4	DIAPH2	0.833	0.11473	1,245,987	1,037,421
Medium	XP_005262724.1	PREDICTED: UDP-glucose 6-dehydrogenase isoform X1 [Homo sapiens]	5	56.4	UGDH	0.813	0.09449	2,068,181	1,680,438
Medium	NP_001272985.1	calponin-3 isoform 3 [Homo sapiens]	10	31.7	CNN3	0.801	0.09412	1,375,016	1,101,403
Medium	NP_001290430.1	calponin-2 isoform d [Homo sapiens]	12	35.9	CNN2	0.782	0.10608	1,257,064	983,289
Medium	AFN01665.1	SND1-BRAF fusion [Homo sapiens]	10	109.1	SND1	0.746	0.02798	26,833,436	20,009,434
Medium	BAD96991.1	aspartate aminotransferase 2 precursor variant, partial [Homo sapiens]	5	47.5	GOT2	0.732	0.0643	3,517,368	2,575,124
Medium	CAH59756.1	glyceraldehyde-3-phosphate dehydrogenase, partial [Homo sapiens]	7	9.2	GAPDH	0.725	0.07239	493,922	357,973
Medium	EAW70091.1	fumarate hydratase, isoform CRA_b [Homo sapiens]	4	54.7	FH	0.664	0.06362	1,431,416	950,301
Medium	BAC03882.1	unnamed protein product [Homo sapiens]	2	48.6	FECH	0.558	0.04354	1,493,226	833,549
Medium	BAG62404.1	unnamed protein product [Homo sapiens]	3	57.8	UGP2	0.537	0.03697	628,828	337,833
Medium	NP_001093242.1	cytochrome P450 4B1 isoform a [Homo sapiens]	1	59	CYP4B1	0.442	0.02779	936,808	423,102
Medium	AAC26738.1	immunoglobulin light chain variable region, partial [Homo sapiens]	1	12.7		0.416	0.01943	1,886,334	784,294
Medium	BAC01721.1	immunoglobulin kappa light chain VLJ region, partial [Homo sapiens]	1	30.2		0.238	0.00451	1,714,045	408,209
Medium	EAX09396.1	ubiquitin-conjugating enzyme E2G 2 (UBC7 homolog, yeast), isoform CRA_b [Homo	1	12.8	UBE2G2	0.193	0.0035	1,136,301	219,654
Medium	NP_001830.1	calponin-3 isoform 1 [Homo sapiens]	13	36.4	CNN3	0.176	0.00147	2,485,758	437,024
Medium	ACR16295.1	immunoglobulin light chain variable region, partial [Homo sapiens]	1	10.1		0.157	0.00017	7,079,370	1,114,502
Medium	ABB69940.1	immunoglobulin light chain variable region, partial [Homo sapiens]	1	11.6		0.123	0.00056	3,669,440	450,092
Medium	ABA71437.1	immunoglobulin kappa chain variable region, partial [Homo sapiens]	2	12		0.01	4.4E-17	38,983,790	#NUM!
Medium	AAB28159.1	anti-colorectal carcinoma heavy chain [Homo sapiens]	7	50.6		0.01	4.4E-17	16,814,060	#NUM!
Medium	BAC01682.1	immunoglobulin kappa light chain VLJ region, partial [Homo sapiens]	1	28.2		0.01	4.4E-17	14,534,427	#NUM!
Medium		S40339 Ig kappa chain - human	1	13.7		0.01	4.4E-17	6,720,703	#NUM!
Medium	CAI56772.1	hypothetical protein [Homo sapiens]	1	25	IGLL5	0.01	4.4E-17	5,976,782	#NUM!
Medium		S40379 Ig kappa chain V-J region - human	1	14.2		0.01	4.4E-17	5,080,081	#NUM!
Medium	BAA36331.1	immunoglobulin heavy chain variable region (IgM), partial [Homo sapiens]	2	13.8		0.01	4.4E-17	4,224,838	#NUM!
Medium	AAA59048.1	This CDS feature is included to show the translation of the corresponding V_region.	2	13		0.01	4.4E-17	2,552,869	#NUM!
Medium	AAW69179.1	anti-tetanus toxoid immunoglobulin light chain variable region, partial [Homo sapie	1	11.6		0.01	4.4E-17	2,088,866	#NUM!
Medium	AAO22179.1	immunoglobulin kappa light chain variable and constant region, partial [Homo sapie	1	11.8		0.01	4.4E-17	1,417,576	#NUM!
Medium	AAB38289.1	immunoglobulin H23 heavy chain variable region, partial [Homo sapiens]	1	13.1		0.01	4.4E-17	1,153,420	#NUM!
Medium	AAQ99260.1	immunoglobulin light chain variable region, partial [Homo sapiens]	1	12.6		0.01	4.4E-17	1,074,724	#NUM!
Medium	AAD15787.1	immunoglobulin G Fd fragment, partial [Homo sapiens]	1	23.8		0.01	4.4E-17	844,120	#NUM!
Medium	AAC83410.1	epidermal cytokeratin 2 [Homo sapiens]	49	65.8	KRT2	0.01	4.4E-17	887,877	#NUM!
Medium	AAH36123.1	Small ArfGAP 1 [Homo sapiens]	2	50.4	SMAP1	0.01	4.4E-17	354,713	#NUM!
Medium	AIL30977.1	immunoglobulin heavy chain variable region, partial [Homo sapiens]	2	13.3		0.01	4.4E-17	1,002,227	#NUM!
Medium	AAB35009.1	antiidiotypic Ig 1F7 light chain variable region [human, 1F7 hybridoma cells, Peptide	1	13.3		0.01	4.4E-17	648,276	#NUM!
Medium	BAA91840.1	unnamed protein product [Homo sapiens]	1	52.7	CNDP2	0.01	4.4E-17	696,169	#NUM!
Medium	XP_011521681.1	PREDICTED: protein spire homolog 2 isoform X1 [Homo sapiens]	1	74.4	SPIRE2	0.01	4.4E-17	600,335	#NUM!
Medium	NP_006861.1	destrin isoform a [Homo sapiens]	1	18.5	DSTN	0.01	4.4E-17	628,398	#NUM!
Medium	NP_005722.1	actin-related protein 2/3 complex subunit 2 [Homo sapiens]	3	34.3	ARPC2	0.01	4.4E-17	609,225	#NUM!
Medium	AIZ70778.1	immunoglobulin heavy chain variable region, partial [Homo sapiens]	1	8.5		0.01	4.4E-17	518,981	#NUM!
Medium	AAC50500.1	pyrroline-5-carboxylate dehydrogenase [Homo sapiens]	2	61.7	ALDH4A1	0.01	4.4E-17	412,776	#NUM!
Medium	AAH88360.1	GRHPR protein, partial [Homo sapiens]	1	36.8	GRHPR	0.01	4.4E-17	382,226	#NUM!
Medium	BAF83214.1	unnamed protein product [Homo sapiens]	2	47.3	ENO2	0.01	4.4E-17	347,443	#NUM!
Medium	BAD92452.1	zinc finger protein 207 variant, partial [Homo sapiens]	1	52.8	ZNF207	0.01	4.4E-17	381,639	#NUM!
Medium	BAC87538.1	unnamed protein product [Homo sapiens]	1	58.3	IGHD	0.01	4.4E-17	360,582	#NUM!
Medium	XP_008970263.1	PREDICTED: mannose-1-phosphate guanyltransferase alpha isoform X1 [Pan panisci	1	52.5	GMPPA	0.01	4.4E-17	338,690	#NUM!
Medium	EAW66872.1	hCG19802, isoform CRA_a, partial [Homo sapiens]	7	41.3		0.01	4.4E-17	319,116	#NUM!
Medium	BAD92609.1	mitogen-activated protein kinase kinase kinase 7 interacting protein 1 isoform alpha	1	70.2		0.01	4.4E-17	254,336	#NUM!
Medium	CAD91892.1	keratin 1b [Homo sapiens]	5	61.8	KRT77	0.01	4.4E-17	202,172	#NUM!
Medium	AAI22559.1	Keratin 77 [Homo sapiens]	5	61.8	KRT77	0.01	4.4E-17	235,009	#NUM!
Medium	BAG70165.1	autoantigen La, partial [Homo sapiens]	1	46.8	SSB	0.01	4.4E-17	166,120	#NUM!
Medium	AAC17932.1	F17127_1 [Homo sapiens]	2	59	SMG9	0.01	4.4E-17	76,830	#NUM!
