# Supplementary material for: Clathrin adapters AP-1 and GGA2 support expression of epidermal growth factor receptor for cell growth
Source: Oncogenesis. 2021 Nov 19;10(11):80. doi: 10.1038/s41389-021-00367-2 (PMC8604998; doi:10.1038/s41389-021-00367-2)
Supplement: Supplementary file 3 — Supplementary Table 2 [file 41389_2021_367_MOESM3_ESM.pdf]

Supplementary Table S2 Detailed information for human samples (related to Materials and Methods, Fig. 8C-E, and Supplemantary Fig. S7E)

・Human tissue array

HCC						
Case	Age	Sex	matched control	Pathology diagnosis	SOURCE	IDENTIFIER
No 1	64	M	yes	Hepatocellular carcinoma	Shanghai Outdo Biotech Co	OD-CT-DgLiv02-005
No 2	59	F	yes			
No 3	57	F	yes			
No 4	45	M	yes			
No 5	61	M	yes			
No 6	19	F	yes			
No 7	71	M	yes			
No 8	53	M	yes			
No 9	63	F	yes			
No 10	50	M	yes			
No 11	32	M	yes			
No 12	38	M	yes			
No 13	71	M	yes			
No 14	64	M	-	Hepatocellular carcinoma	Shanghai Outdo Biotech Co	OD-CT-DgLiv02-004
No 15	59	F	-			
No 16	42	M	-			
No 17	48	M	-			
No 18	37	M	-			
No 19	56	M	-			
No 20	54	M	-			
No 21	57	F	-			
No 22	45	M	-			
No 23	43	M	-			
No 24	58	M	-			
No 25	61	M	-			
No 26	63	M	-			
No 27	19	F	-			
No 28	39	M	-			
No 29	77	M	-			
No 30	71	M	-			
No 31	53	M	-			
No 32	72	M	-			
No 33	50	M	-			
No 34	48	M	-			
No 35	52	M	-			
No 36	63	F	-			
No 37	50	M	-			
No 38	58	F	-			
No 39	39	M	-			
No 40	41	M	-			
No 41	38	F	-			
No 42	51	M	-			
No 43	49	M	-			
No 44	32	M	-			

No 45	55	M	-			
No 46	31	M	-			
No 47	60	M	-			
No 48	40	M	-			
No 49	38	M	-			
No 50	36	M	-			
No 51	71	M	-			

NSCLC						
Case	Age	Sex	matched control	Pathology diagnosis	SOURCE	IDENTIFIER
No 1	78	M	yes	Squamous cell carcinoma	Shanghai Outdo Biotech Co	OD-CT-RsLug01-007
No 2	64	M	yes			
No 3	65	M	yes			
No 4	70	M	yes			
No 5	61	M	yes			
No 6	55	M	yes	Adenocarcinoma		
No 7	56	F	yes			
No 8	49	M	yes			
No 9	73	M	yes			
No 10	46	M	yes			
No 11	74	M	yes	Adenosquamous carcinoma		
No 12	75	M	yes			
No 13	45	M	yes			
No 14	65	M	yes			
No 15	73	M	yes			
No 16	76	M	yes	Squamous cell carcinoma		
No 17	67	M	yes			
No 18	58	M	yes			
No 19	64	M	yes			
No 20	61	M	yes			
No 21	59	F	yes	Adenocarcinoma		
No 22	60	F	yes			
No 23	57	F	yes			
No 24	75	F	yes			
No 25	45	M	yes			
No 26	50	M	yes	Adenosquamous carcinoma		
No 27	55	M	yes			
No 28	58	M	yes			
No 29	65	F	yes			
No 30	56	F	yes			
No 31	76	F	yes	Bronchioalveolar carcinoma		
No 32	58	F	yes			
No 33	73	M	yes			
No 34	57	M	yes			
No 35	69	F	yes			
No 36	49	M	yes	Large cell carcinoma		
No 37	49	M	yes			
No 38	62	M	yes			
No 39	59	M	yes			
No 40	72	M	yes			

No 41	50	F	yes	Bronchioalveolar carcinoma		
No 42	62	F	yes			
No 43	52	M	yes			
No 44	71	F	yes			
No 45	59	F	yes			
No 46	71	M	yes	Large cell carcinoma		
No 47	54	M	yes			
No 48	75	M	yes			
No 49	75	M	yes			
No 50	56	F	-			

CRC						
Case	Age	Sex	matched control	Pathology diagnosis	SOURCE	IDENTIFIER
No 1	83	F	yes	Colon adenocarcinoma	Shanghai Outdo Biotech Co	HCol-Ade060PG-01
No 2	72	M	yes			
No 3	80	F	yes			
No 4	60	M	yes			
No 5	69	M	-			
No 6	74	F	yes			
No 7	52	M	yes			
No 8	41	M	yes			
No 9	64	F	yes			
No 10	70	F	yes			
No 11	41	F	yes			
No 12	49	M	yes			
No 13	43	M	yes			
No 14	66	M	yes			
No 15	64	M	yes			
No 16	42	F	yes			
No 17	33	M	yes			
No 18	39	F	yes			
No 19	53	F	yes			
No 20	83	F	-			
No 21	71	F	yes			
No 22	60	F	yes			
No 23	64	F	yes			
No 24	74	M	yes			
No 25	52	M	yes			
No 26	75	F	yes			
No 27	47	M	yes			
No 28	62	M	yes			
No 29	76	F	yes			
No 30	57	M	yes			
